# RNA viruses, M satellites, chromosomal killer genes, and killer/nonkiller phenotypes in the 100-genomes *S. cerevisiae* strains

**DOI:** 10.1093/g3journal/jkad167

**Published:** 2023-07-27

**Authors:** Sriram Vijayraghavan, Stanislav G Kozmin, Pooja K Strope, Daniel A Skelly, Paul M Magwene, Fred S Dietrich, John H McCusker

**Affiliations:** Department of Molecular Genetics and Microbiology, Duke University Medical Center, Durham, NC 27710, USA; Department of Molecular Genetics and Microbiology, Duke University Medical Center, Durham, NC 27710, USA; Department of Molecular Genetics and Microbiology, Duke University Medical Center, Durham, NC 27710, USA; Department of Biology, Duke University, Durham, NC 27708, USA; Department of Biology, Duke University, Durham, NC 27708, USA; Department of Molecular Genetics and Microbiology, Duke University Medical Center, Durham, NC 27710, USA; Department of Molecular Genetics and Microbiology, Duke University Medical Center, Durham, NC 27710, USA

**Keywords:** RNA virus, *Saccharomyces cerevisiae*, killer, phenotypes, associations, 100-genomes strains

## Abstract

We characterized previously identified RNA viruses (L-A, L-BC, 20S, and 23S), L-A–dependent M satellites (M1, M2, M28, and Mlus), and M satellite–dependent killer phenotypes in the *Saccharomyces cerevisiae* 100-genomes genetic resource population. L-BC was present in all strains, albeit in 2 distinct levels, L-BC^hi^ and L-BC^lo^; the L-BC level is associated with the L-BC genotype. L-BC^hi^, L-A, 20S, 23S, M1, M2, and Mlus (M28 was absent) were in fewer strains than the similarly inherited 2µ plasmid. Novel L-A–dependent phenotypes were identified. Ten M^+^ strains exhibited M satellite–dependent killing (K^+^) of at least 1 of the naturally M^0^ and cured M^0^ derivatives of the 100-genomes strains; in these M^0^ strains, sensitivities to K1^+^, K2^+^, and K28^+^ strains varied. Finally, to complement our M satellite–encoded killer toxin analysis, we assembled the chromosomal *KHS1* and *KHR1* killer genes and used naturally M^0^ and cured M^0^ derivatives of the 100-genomes strains to assess and characterize the chromosomal killer phenotypes.

## Introduction

Like many other species of yeasts and fungi (McCabe *et al*. 1999; Nuss 2005; Ghabrial and Suzuki 2009; Pearson *et al*. 2009; Roossinck 2011; Xie and Jiang 2014; Ghabrial *et al*. 2015), the yeast *Saccharomyces cerevisiae* can host symbiotic cytoplasmically transmitted RNA viruses, which include 2 narnaviruses, 20S (2,514 bases) and 23S (2,891 bases), and 2 totiviruses, L-A (4,579 bp) and L-BC (4,615 bp), reviewed in (Wickner 1992, 1996a, 1996b; Wickner *et al*. 2013; Rowley 2017). In addition, L-A–containing *S. cerevisiae* strains also may host any 1 of 4 satellite (that is, requiring L-A for replication) RNAs—M1 (1.6 kbp), M2 (1.5 kbp), M28 (1.75 kbp), and Mlus (2.1–2.3 kbp). Fully functional M1, M2, M28, and Mlus satellites encode secreted K1, K2, K28, and Klus killer toxins (K^+^), respectively, and corresponding killer toxin–specific resistance (R^+^) functions, resulting in *S. cerevisiae* strains with 4 distinct M satellite–dependent killer phenotypes (Magliani *et al*. 1997; Schmitt and Breinig 2002, 2006; Rodriguez-Cousiño *et al.* 2011).

Although the RNA viruses, M satellites, and M satellite–dependent killer phenotypes of *S. cerevisiae* have been extensively characterized (Wickner 1992, 1996a, 1996b; Magliani *et al*. 1997; Schmitt and Breinig 2002, 2006; Rodriguez-Cousiño *et al.* 2011; Wickner *et al*. 2013; Rodriguez-Cousino and Esteban 2017; Rowley 2017), most of this characterization appears to have been done in laboratory strains rather than population studies. Of the previous population studies, most focused on assessing the frequencies in *S. cerevisiae* strains of killer phenotypes (Philliskirk and Young 1975; Rosini 1983; Nakayashiki *et al*. 2005; Baeza *et al*. 2008; Rodriguez-Cousiño *et al.* 2011; Pieczynska *et al*. 2013; Chang *et al*. 2015) but did so using different techniques and to different extents. With respect to killer phenotypes, some of these previous population studies identified killer *S. cerevisiae* strains using only 1 killer toxin–sensitive strain, some identified the killer type(s), some determined the M satellite dependence of killer phenotype(s), and some assayed M satellite–independent resistance to known killer strain(s). With respect to the RNA viruses, previous population studies used northern analysis to assess the frequencies of 20S and 23S (Lopez *et al*. 2002) and PCR with 1 primer pair each to the L-A, L-BC, 20S, and 23S viruses to assess their frequencies (Nakayashiki *et al*. 2005). Nevertheless, no single study has used multiple, consistent techniques to explore RNA viruses, M satellites, and killer phenotypes across a broad set of genetically diverse, well-characterized strains.

Our *S. cerevisiae* 100-genomes population studies described their nuclear genome sequences, population structure, phenotypes, and genotype associations (Strope *et al*. 2015b), as well as their 2µ plasmid (Strope *et al*. 2015a) and mitochondrial genomes (Vijayraghavan *et al*. 2019). Unlike these DNA genome components, however, RNA viruses and M satellites are not assessed by genome sequencing. Thus, in this study, we assessed the presence in the 100-genomes strains of the abovementioned RNA viruses and M satellites, as well as their M satellite–dependent killer phenotypes. We cured L-A from most L-A^+^ strains and determined the phenotypes of isogenic L-A^+^ vs L-A^0^ strains, finding novel L-A–dependent phenotypes. In naturally M satellite–free and M satellite–cured 100-genomes strains, we determined M satellite–independent K1, K2, K28, and Klus killer toxin resistance phenotypes.

Finally, in contrast to the extensive work in *S. cerevisiae* on M satellites and their encoded killer toxins–antitoxins (Magliani *et al*. 1997; Schmitt and Breinig 2002, 2006; Rodriguez-Cousiño *et al.* 2011), there has been much less work on chromosomally encoded killer genes and their killer toxins, *KHR1* (killer toxin, heat resistant) (Goto *et al*. 1990b) and *KHS1* (killer toxin, heat sensitive) (Goto *et al*. 1991). Thus, we assembled and analyzed the *KHR1* and *KHS1* genes in the 100-genomes strains. In naturally M satellite–free and M satellite–cured 100-genomes strains, we determined chromosomal killer phenotypes and performed a detailed analysis of *KHS1*. We discuss the implications of our results on RNA virus, M satellite, and chromosomal killer effects on fitness, balancing selection on resistance to killer toxins, the analysis of host virus–satellite interactions, and the analysis of host phenotypes.

## Methods

### Strains

The *S. cerevisiae* strains listed in Supplementary Table 1 have been deposited in and should be requested from the Fungal Genetics Stock Center (McCluskey *et al*. 2010): http://www.fgsc.net. Unless explicitly stated otherwise, all strains were *MATa*/*MATα* diploids. For additional descriptions of the sequenced 100-genomes *S. cerevisiae* strains, or genetic backgrounds, listed in Supplementary Table 1, see (Strope *et al*. 2015a, 2015b; Vijayraghavan *et al*. 2019). The reference M28-containing K28 killer strain MS300c (Schmitt and Tipper 1990) and the reference Mlus-containing Klus killer strain EX229 (Rodriguez-Cousiño *et al.* 2011) were obtained and should be requested from Manfred Schmitt and Manuel Ramirez, respectively. The type strain of *Candida glabrata* (ATCC 2001 = IFO 0622 = CBS 138 = NRRL Y-65), which previously was used to test for and characterize *KHS1*-dependent (Goto *et al*. 1991) and *KHR1*-dependent (Goto *et al*. 1990a, 1990b) killing and to isolate the corresponding genes, should be requested from the ATCC, NBRC, CBS-KNAW, or NRRL culture collections. Isogenic derivatives of YJM189 (*HO*; self-diploidized) were constructed by deleting 1 copy of a gene (i.e. *KHS1*, *KHR1*, *KEX1*, and *KEX2*), sporulating the resulting +/Δ diploids and dissecting tetrads. From each of the +/Δ diploids, the segregants from 1 tetrad were phenotypically tested for Khr1- and/or Khs1-mediated killing.

### *KHS1* and *KHR1* assemblies

The chromosomal killer genes *KHS1* (Goto *et al*. 1991), as corrected by (Frank and Wolfe 2009), and *KHR1* (Goto *et al*. 1990a, 1990b) were assembled and annotated using 100-genomes strains sequence data (Strope *et al*. 2015b).

### Media and phenotypic analysis

YPD and YPE (1% yeast extract, 2% bacto peptone) contained 2% dextrose and 2% ethanol (added after autoclaving), respectively; plates contained 2% agar. To perform high-throughput nonkiller phenotypic analysis of *S. cerevisiae* strains, we used an S&P Robotics BM5-BC colony handling robot with imaging scanner/software for quantitative colony diameter phenotypes, as described previously (Strope *et al*. 2015b). We performed low-throughput nonkiller phenotypic analysis on subsets of strains by spot dilutions onto 100-mm diameter plates. That is, starting with cell suspensions of each tested strain (10^8^ cells/ml), we serially diluted 1:10 in sterile deionized H_2_O and spotted 5 µl from each dilution onto control and experimental media to assess phenotypes.

### Killer media and killer phenotypic analysis

We used MB medium [YPD: 1% yeast extract, 2% bacto peptone, 2% dextrose, and 2% agar, plus 0.003% methylene blue (methylene blue stains dead yeast cells; see citation in (Fink and Styles 1972))] (Fink and Styles 1972; Schmitt and Tipper 1990; Rodriguez-Cousiño *et al.* 2011) to determine killer phenotypes. When testing for K1, K2, Klus, and Khs1 killer phenotypes at pH 4.7 (final pH), both methylene blue (3 ml of 1% filter sterile solution) and 100 ml of 1 M citrate (sterile, adjusted to pH 4.5 with K_3_PO_4_) were added to 900 ml of medium after autoclaving. When testing for K28 and Khr1 killer phenotypes at pH6 (final pH), both methylene blue (3 ml of 1% filter sterile solution) and 100 ml of 1 M citrate (adjusted to pH6 with K_3_PO_4_; sterile) were added to 900 ml of medium after autoclaving. Like Goto *et al*. (Goto *et al*. 1990a, 1990b), we also used a synthetic defined medium (SD + MB) (final concentrations: 0.67% yeast nitrogen base, 2% dextrose, 2% agar, and 0.003% methylene blue) to test chromosomal *KHR1* killer phenotypes.

In addition to 1 reference L-A^+^ M28^+^ K28^+^ strain (MS300c (Schmitt and Tipper 1990)) and 1 reference L-A^+^ Mlus^+^ Klus^+^ strain (EX229 (Rodriguez-Cousiño *et al.* 2011)), we determined the killer phenotypes of L-A^+^ M^+^ 100-genomes strains (M1^+^: *n* = 5; M2^+^: *n* = 3; Mlus^+^: *n* = 7; unknown M^+^: *n* = 2), as well as their isogenic L-A^0^ M^0^ derivatives. Briefly, strains being tested for killer activity were patched onto MB plates that had been separately seeded with approximately 10^5^ cells/plate of each of the 100-genomes strains that were naturally M^0^, as well as isogenic L-A^0^ (M^0^) derivatives of L-A^+^ 100-genomes strains, or with *C. glabrata* (ATCC 2001), to create lawns. These plates were incubated in the dark at 20°C (K1, K2, Khs1, and Khr1) and 30°C (K28, Klus) for 1 to 3 days after which killing was scored by killing zone sizes (qualitative killing zone bin sizes in decreasing order of killing: 2, 1, 0.5, and 0) and killing staining [qualitative cell killing staining bins: 2 (clear killing zone), 1 (turbid killing zone), 0.5 (dark blue ring), and 0 (no staining)], around the patched strains on the lawns.

### Virus and M satellite presence/absence determination by PCR and gel electrophoresis

Total nucleic acids were extracted using a slight modification of the phenol-mediated method described by (Maqueda *et al*. 2010), as follows. Cells of 5-ml overnight YPD cultures were collected by centrifugation and washed once with 50 mM Na_2_EDTA (pH 7.5). Cell pellets were then resuspended in 1 ml of 50 mM Tris-H_2_SO_4_ (pH 9.3)/1% 2-mercaptoethanol solution and incubated for 15 minutes at room temperature. Cells were subsequently collected by centrifugation and resuspended in 1 ml of 0.1 M NaCl/10 mM Tris-HCl (pH 7.5)/10 mM Na_2_EDTA/0.2% sodium dodecyl sulfate solution. 0.7 ml of phenol (pH 8.0) was added, and samples were incubated on a shaking platform for 1 h at room temperature, after which 0.7 ml of the aqueous phase was recovered by 5-minute centrifugation. Nucleic acids were precipitated by the addition of 70 µl of 3 M potassium acetate and 0.7 ml of isopropanol, followed by incubation for 5 minutes at room temperature and centrifugation at 14,000 rpm for 10 minutes. The precipitated nucleic acids were washed with 70% ethanol, dried using a Speedvac (Eppendorf), and dissolved in 100 µl of water. These samples were stored at −80°C.

PCR analysis of previously described RNA viruses and M satellites was performed as follows. 15-µl aliquots of total nucleic acid samples were incubated for 2 minutes at 98°C and then placed on ice. 5-µl aliquots of these samples were used as a template for cDNA synthesis performed using the Maxima First-Strand cDNA Synthesis Kit (Thermo Fisher Scientific) in accordance with the manufacturer's protocol. One-tenth of the cDNA reaction volume was used in virus-diagnostic PCR performed with OneTaq DNA Polymerase (New England Biolabs) in accordance with the manufacturer's protocol. The primers used to detect the presence of previously described RNA viruses and M satellites are listed in Supplementary Table 2.

PCRs of each of the known RNA viruses and M satellites were performed with multiple primer pairs to determine the presence/absence (Supplementary Table 3 and Supplementary Fig. 1). In addition, for a given primer pair, strain-to-strain variation in PCR product formation (+ vs −), which may be influenced by the template level as well as template sequence variation, was observed, which is referred to as a genotype or PCR genotype (Supplementary Table 3). For all RNA viruses and M satellites (high abundance: L-A; low abundance: M satellites, 20S, 23S; high and low abundance: L-BC), PCR genotypes were reproducible.

Relative quantification of L-BC levels in YJM1463 and YJM1529 (L-A^+^ strains that could not be cured of L-A by any technique, see below) was determined using the Luna Universal One-Step RT-qPCR Kit (New England Biolabs) using SYBR chemistry according to the manufacturer's recommendations. Real-time PCR was performed using QuantStudio 6 (Applied Biosystems). L-BC quantification was performed relative to *UBC6* using primers described in Supplementary Table 2, with relative expression calculated as 2^−ΔΔEqCq^ using the QuantStudio Design and Analysis software (Applied Biosystems).

For gel electrophoresis, 5-μl aliquots of total nucleic acid samples (to detect abundant RNA viruses and satellites) or qPCR-derived nucleic acid samples (to detect PCR products of sequenced RNA viruses and M satellites) were mixed with 1× gel-loading buffer and loaded on 1.3% agarose gels prestained with Green Gene dye (Southern Biolabs) (and/or ethidium bromide; BioRad) according to the manufacturer's instructions. Electrophoresis was carried out in 1× TAE buffer at room temperature at a constant voltage of 6 V/cm for 45–60 minutes. Gels were subsequently imaged under UV light using the Alpha Innotech Red gel documentation system.

### Nuclease treatments

Nucleic acid samples were treated with DNaseI (New England Biolabs) at 37°C, and samples were analyzed on 1.2% TAE agarose gel stained with ethidium bromide. For subsequent analyses, treated samples were column purified using standard PCR purification kits (Qiagen), reverse transcribed with Maxima First-Strand cDNA Synthesis Kit (Thermo Fisher Scientific), and subjected to PCR to test for the presence/absence of various genomic and viral elements, as described above. PCR samples were analyzed on 2% TBE (Tris-borate EDTA) gels at 4–6 V/cm for 75–90 minutes in prechilled 0.5× TBE buffer.

### Curing L-A

We used previously described techniques (Valle and Wickner 1993; Dinman *et al*. 1997; Esteban *et al*. 2008; Drinnenberg *et al*. 2011) and a novel technique described below, to cure 28 of 30 L-A^+^ strains of L-A; the remaining 2 L-A^+^ strains, YJM1463 and YJM1529, were refractory to curing by all methods. In the case of L-A curing with plasmid-borne L-A cDNA, we made and used a nourseothricin resistance (Nat) containing derivative of pI2L2 (Wickner *et al*. 1991; Valle and Wickner 1993) (obtained from J. Dinman as pJD1223), pJD1223-NAT (Supplementary Table 1, Technique I). Because these previously described techniques failed to cure L-A from some strains, we also used the requirement of Mak3 and Mak10 (Ball *et al*. 1984), components of the NatC N-terminal acetyltransferase, for L-A propagation to cure L-A. We crossed isogenic haploid *mak3*Δ and *mak10*Δ derivatives and, after sporulation and tetrad dissection, isolated Mak^+^ segregants that were then diploidized using the HO-containing plasmid pHS3 (Supplementary Table 2) (Supplementary Table 1, Technique II). We also developed a novel technique to cure L-A that combines the requirement of Mak10p for L-A propagation (Ball *et al*. 1984) with the positive/negative selection *amdS* cassette (Solis-Escalante *et al*. 2013), which we obtained from EUROSCARF. Specifically, we cured L-A by introducing a recombinationally reversible *mak10* disruption mutation. That is, we integrated a (*amdS* + internal fragment of *mak10* ORF)–containing plasmid (pUG-amdSYM-mak10TR) into haploid *MAK10* strains, selecting for acetamide utilization and subsequently for recombinational plasmid pop outs, selecting for fluoroacetamide resistance; that is, *MAK10* L-A^+^ → *mak10*::amdS L-A^0^ → *MAK10* L-A^0^; these Mak^+^ derivatives were then diploidized as described above. pUG-amdSYM-mak10TR is a pUG-amdSYM (Solis-Escalante *et al*. 2013) derivative. An *Nde*I-cloned 734-bp internal fragment of the S288C-derived *MAK10* open reading frame was amplified using primers mak10TR-Nde-F and mak10TR-Nde-R (Supplementary Table 2) (Supplementary Table 1, Technique III) and cloned into an *Nde*I site of pUG-amdSYM. In all cases, curing of L-A (and cocuring of M, if present) was determined by gel electrophoresis (in L-BC^lo^ strains) and/or by PCR. Plasmids described above (with the exception of pJD1223-NAT, the loss of which was not realized until after the shutdown of the McCusker laboratory) have been deposited with, and should be requested from, Addgene http://www.addgene.org/John_McCusker/.

### Curing M satellites

M satellites from L-A^+^ M^+^ strains were cured essentially as described (Fink and Styles 1972; Carroll and Wickner 1995; Rodriguez-Cousiño *et al.* 2011). Briefly, overnight log-phase cultures of L-A^+^ M^+^ strains were obtained by growth in liquid YPD at 30°C. Cultures were subsequently serially passaged twice for 12 hours each in fresh YPD containing 0.05 mg/L cycloheximide at 30°C. Cells from cycloheximide-treated cultures were streaked on YPD plates without cycloheximide to isolate single colonies, which were tested by PCR for retention of L-A and for curing of M.

### Testing correlations between RNA viruses, M satellites, and narnaviruses

Fisher's exact test was used to test for correlation between the L-A, L-BC, 20S, and 23S viruses, as well as the M1 (*n* = 5) and Mlus (*n* = 7) satellites (Supplementary Table 4), with previously determined (Strope *et al*. 2015b) population ancestry and clinical/nonclinical origin using Prism (GraphPad).

### M satellite–independent K1, K2, K28, and Klus toxin sensitivity/resistance phenotypes

To avoid the confounding effects of M-dependent killer toxin resistance, we determined the killer toxin sensitivity/resistance phenotypes of 100-genomes strains that were naturally M^0^ and of L-A^0^ M^0^ derivatives of the 100-genomes strains. We tested for M satellite–independent killer toxin sensitivity/resistance phenotypes with M1^+^ (K1^+^: YJM1077, YJM1290, and YJM1307; K1^±^: YJM1387; and K^−^: YJM1419), M2^+^ (K2^+^: YJM1341, YJM453, and YJM1574 that have different levels of K2 activity), M28^+^ (the reference K28^+^ strain, MS300c), and Mlus^+^ Klus^+^ (the reference Klus^+^ EX229 strain) strains (Supplementary Table 5).

### Genotype associations

Based on the genome sequences of the 100 yeast genome strains (Strope *et al*. 2015b), we identified 142,313 variable sites with MAF ≥ 0.05. The “–indep” option of PLINK (1.90b6.26) (Chang *et al.* 2015) was used to prune sites in linkage disequilibrium (*R*^2^ > 0.5), resulting in a final set of 19,061 biallelic sites. These pruned sites were used to estimate a genetic relationship matrix using the GTCA software package (1.94.1) (Yang *et al.* 2011). Association analysis was carried out using the mixed linear model GWAS command in GCTA (fastGWA-MLM) (Jiang *et al.* 2019), with account for population structure using a sparse genetic relationship matrix derived from the full relationship matrix using a threshold of 0.05. The ordinal scores for each phenotype of interest (Supplementary Tables 5 and 6) were normalized to a standard scale (0, 1, 2, and 3) before carrying out GWAS. Thresholds for significance were established by generating 1,000 permutations of the original phenotypes and refitting the fastGWA model to each permutation. The 0.05 quantile of the distribution of minimum *P*-values from each permutation was used to estimate *P*-value thresholds of 2.96 × 10^−6^ and 1.44 × 10^−9^ for *C. glabrata* killing and killing by YJM189, respectively. To account for the possibility that small differences in the rank ordering of phenotypes might lead to false positive associations, we fit the GWAS model to “jittered” replicates of each phenotypic data set in which the phenotypes were adjusted by adding normally distributed random values (mean = 0, SD = 0.25) to each phenotypic score. This has the effect of randomizing rank-order within each phenotypic class, while maintaining rank-order between classes. We generated 100 such jittered data sets and here report only those peaks that exceed the significance threshold in at least 70% of the jittered replicates.

Analyses of statistical significance were performed using chi square analysis or Fisher's exact test, where applicable.

## Results and discussion

### L-A, L-BC, 20S, and 23S RNA viruses in the *S. cerevisiae* 100-genomes strains

Integrated RNA virus and satellite cDNAs, for which there are precedents in yeast nuclear genomes (Frank and Wolfe 2009), might confound PCR detection of RNA viruses and M satellites. We found no sequences with high homology to L-A, L-BC, 20S, 23S, M1, M2, or Mlus in our previous analysis of the nuclear genomes of the 100-genomes strains (Strope *et al*. 2015b). However, we identified a 713-bp sequence with 82% similarity to the *S. cerevisiae* M28 1.75-kbp dsRNA (Schmitt and Tipper 1995) in the nuclear genome of 1 strain, YJM195 (Strope *et al*. 2015b). Therefore, except for the partial M28 cDNA in the nuclear genome of YJM195, RNA virus and M satellite cDNAs did not confound PCR detection of RNA viruses or M satellites in the 100-genomes strains.

We used PCR to test the 100-genomes strain population (Strope *et al*. 2015b) for the presence/absence and, where present, the PCR genotypes of the L-A and L-BC totiviruses (Table 1 and Supplementary Tables 2 and 3). Because PCR would not detect novel totiviruses and might not detect sequence variants of the L-A and L-BC totiviruses, we also used gel electrophoresis to test for the presence/absence of bands corresponding in size to the abundant L-A and L-BC totiviruses both before and after curing L-A.

**Table 1.** Summary of PCR analysis of the 100-genomes strains for RNA viruses and M satellites.

Virus/M	Strains	Primer pairs	PCR genotypes
L-A	30	7	6
L-BC	100	3	6
20S	26	2	3
23S	14	2	3
M1	5	2	1
M2	3	2	1
M28	0	2	NA
Mlus	7	5	1

Virus/M: previously described RNA viruses (L-A, L-BC, 20S, and 23S) and L-A–dependent M satellites (M1, M2, M28, and Mlus); strains: number of 100-genomes strains containing a specific RNA virus/M satellite as determined by PCR product(s); primer pairs: number of virus- or M satellite–specific primer pair(s) (Supplementary Table 2); PCR genotypes: for ≥2 primer pairs, virus genotype–specific combinations of PCR products. One strain, YJM195, which contains a partial M28 cDNA (Strope *et al*. 2015b), yielded a PCR product with 1 of 2 M28-specific primer pairs. NA, not applicable. Individual strain data (Supplementary Table 3).

By PCR with multiple primer pairs, 30 of the 100-genomes strains were L-A^+^ and all were L-BC^+^ (Table 1). However, despite all the strains being L-BC^+^, gel electrophoresis of total RNA revealed a 4.6-kb band in only 47 strains. (There were no gel bands greater than 4.6 kb, the approximate size of L-A and L-BC, in any of the 100-genomes strains.) L-BC abundance in the naturally L-A^0^ strains (*n* = 70) and L-A–cured (L-A^+^ → L-A^0^; *n* = 28) strains fell into 2 categories—L-BC^hi^ (readily discernable 4.6-kb gel band: *n* = 31) and L-BC^lo^ (no discernable 4.6-kb gel band: *n* = 67) (Supplementary Table 3). For the 2 L-A^+^ L-BC^+^ strains where L-A could not be cured, real-time PCR was performed, from which it was determined that YJM1529 was L-BC^hi^ and YJM1463 was L-BC^lo^ (Supplementary Table 3 and Supplementary Fig. 5). We found that the L-BC PCR genotype and L-BC level are associated (Table 2; Supplementary Table 4), consistent with the L-BC genotype having a major effect on the L-BC level. The combined results of gel electrophoresis and PCR with multiple primer pairs suggest that we identified all L-A and L-BC totiviruses and that there were no novel, abundant totiviruses in the 100-genomes strains.

**Table 2.** Summary of L-BC PCR genotypes and L-BC levels.

L-BC PCR genotype	L-BC^hi^	L-BC^lo^
0,0,1	1	2
0,1,0	0	49
0,1,1	13	3
1,0,0	0	0
1,0,1	5	2
1,1,0	0	1
1,1,1	14	10
Total:	33	67

L-BC PCR genotype: for each of the 3 L-BC primer pairs (Supplementary Tables 2 and 3)—1, PCR product; 0, no PCR product; 6 of the 7 possible L-BC PCR genotypes were observed. L-BC^hi^ and L-BC^lo^: gel analysis of naturally L-A^0^ (*n* = 70) and L-A^+^ → L-A^0^ (cured: *n* = 28) strains assessed the presence (L-BC^hi^: *n* = 32) vs absence (L-BC^lo^: *n* = 66) of an abundant 4.6-kb gel band. L-BC levels in the 2 remaining L-A^+^ L-BC^+^ strains were assessed by real-time PCR. L-BC level associated with the PCR genotype (Supplementary Table 4). Individual strain data (Supplementary Table 3).

L-BC, like L-A and its variants (Sommer and Wickner 1982; El-Sherbeini *et al*. 1984; Rodriguez-Cousino *et al*. 2013; Rodriguez-Cousino and Esteban 2017), appears to have been presumed to exist only at high copy numbers sufficient to be readily observable on gels. However, a previous study described low-level L-BC in some strains and hypothesized this as being due to a complex chromosomal defect [chromosomal L-0 (Clo^−^)] (Wesolowski and Wickner 1984). Consistent with such complexity, we suggest that both the L-BC genotype and the host genotype contribute to the L-BC level.

In a previous work, we identified multiple examples of introgression from other Saccharomyces species in the *S. cerevisiae* 100-genomes strains (Strope *et al*. 2015b). Because species-specific *XRN1*-L-A interactions have been identified (Rowley *et al*. 2016), we considered the hypothesis that *XRN1* introgression, or loss of function polymorphisms, might contribute to L-A presence/absence or, possibly, L-BC levels in the 100-genomes strains. However, we did not identify any cases of introgression, LTR insertions, or ORF length polymorphisms (indels/frameshifts, premature stop codons) in the *XRN1* genes, nor in any of the *SKI* genes, in the 100-genomes strains (Strope *et al*. 2015b).

Strains were grown under nonstress conditions (YPD at 30°C) where 20S and 23S were expected to be present in low abundance and hence not detectable by gel. Thus, as above, PCR on total nucleic acids isolated from the 100-genomes strains was used to assess the presence of the 20S and 23S narnaviruses. Twenty-six strains were 20S^+^ and 14 were 23S^+^, with both narnaviruses having multiple PCR genotypes (Table 1; Supplementary Table 3).

In *S. cerevisiae*, RNA viruses and 2µ plasmid are inherited in a similar fashion. Specifically, after the mating of haploid “+” and “−” strains followed by meiosis, there is 4+:0− segregation. The 2µ plasmid has low loss rates and deleterious effects on fitness, either alone or in combination with specific nuclear genome mutations (Holm 1982a, 1982b; Futcher and Cox 1983; Mead *et al*. 1986; Dobson *et al*. 2005; Harrison *et al*. 2012). However, we found 2µ plasmid in more of the 100-genomes strains (*n* = 72) (Strope *et al*. 2015a) than the L-A (*n* = 30), L-BC^hi^ (*n* = 33), 20S (*n* = 26), and 23S (*n* = 14) RNA viruses. The higher frequency of 2µ plasmid relative to the RNA viruses may be influenced by population structure and/or by time(s) of entry into the *S. cerevisiae* population. Alternatively, the higher frequency of 2µ plasmid may be due to these *S. cerevisiae* RNA viruses having higher loss rates, possibly due to the high mutation frequency characteristic of RNA viruses (Sanjuan *et al*. 2010), and/or higher fitness costs.

### L-A–dependent M satellites, M satellite–dependent killer phenotypes, and M satellite–independent killer resistance phenotypes in the *S. cerevisiae* 100-genomes strains

**Fig. 1.**
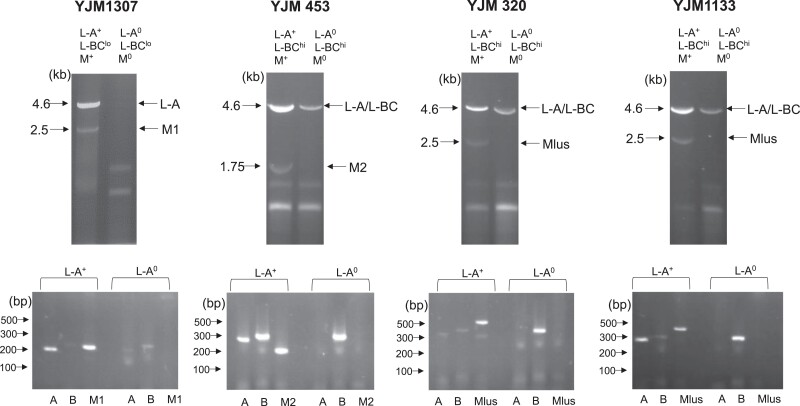
Representative gels (top) of strains carrying different species of the dsRNA totiviruses (L-A and L-BC) and satellites (M1, M2, and Mlus). Shown are isogenic strain pairs of native and L-A–cured strains. (Bottom) Corresponding PCRs from cDNAs of RNA derived from the indicated strains, probing for presence/absence of L-A (A), L-BC (B), or M (M1, M2, and Mlus). All samples were treated with DNase prior to gel and PCR analysis. Primers for PCR analysis LA-F4 + LA-R5 (L-A), LBC-F2 + LBC-R2 (L-BC), M1-F + M1-R (M1), M2-F + M2-R (M2), and Mlus-F2 + Mlus-R2 (Mlus).

Using multiple primer pairs, we used PCR to test the 100-genomes strains population for the presence/absence of the L-A–dependent satellites M1, M2, M28, and Mlus. Sixteen strains, all of which were L-A^+^, were M^+^ by PCR; 5 strains were M1^+^, 3 were M2^+^, 7 were Mlus^+^, and 1 was M28^+^ (Table 3). The 1 L-A^+^ M28^+^ strain, YJM195, contained a previously identified partial M28 cDNA in its nuclear genome (Strope *et al*. 2015b). In YJM195, curing L-A cocured an unknown L-A–dependent M satellite (see below) but not the chromosomally encoded M28 cDNA-dependent PCR product (Supplementary Fig. 2). In contrast, in the remaining 15 L-A^+^ M^+^ strains, curing L-A cocured the known species of M, as determined by PCR; that is, L-A^+^ M^+^ → L-A^0^ M^0^ (Table 1; Supplementary Table 3; Fig. 1. Each of these 15 M^+^ strains contained a single species of M satellite, consistent with M exclusion (Schmitt and Tipper 1992). In contrast to L-A and L-BC, no PCR genotype variation was observed for these M satellites.

**Table 3.** Summary of L-A–dependent M satellite–containing 100-genomes strains and M satellite–dependent killer phenotypes.

Strain	M species	Gel band (<4.6 kb)	Killer
YJM1077	M1^+^	—	K1^+^
YJM1290	M1^+^	—	K1^+^
YJM1307	M1^+^	2.5 kb	K1^+^
YJM1387	M1^+^	—	K1^+/−^
YJM1419	M1^+^	—	K1^−^
YJM453	M2^+^	1.7 kb	K2^+^
YJM1341	M2^+^	—	K2^+^
YJM1574	M2^+^	—	K2^+^
YJM145	Mlus^+^	2.5 kb	Klus^−^
YJM320	Mlus^+^	2.5 kb	Klus^+^
YJM975	Mlus^+^	2.5 kb	Klus^−^
YJM978	Mlus^+^	2.5 kb	Klus^−^
YJM993	Mlus^+^	2.5 kb	Klus^−^
YJM1133	Mlus^+^	2.5 kb	Klus^+^
YJM1526	Mlus^+^	2 kb	Klus^+^
YJM195	0	3 kb	K^−^
YJM681	0	2.5 kb	K^−^

All listed strains were L-A^+^. M species (+): the presence of a previously described species of M as determined by PCR; by PCR, each M^+^ strain contained 1 M species. 0, no PCR products with any M primers (Supplementary Table 2). Gel band: the presence of a <4.6-kb dsRNA gel band. Upon curing of L-A, all listed L-A^+^ M^+^ strains were L-A^0^ M^0^ by gel and/or PCR and all K^+^ strains were K^−^, consistent with an L-A–dependent M satellite. YJM195, which contains a partial M28 cDNA (Strope *et al*. 2015b), yielded a PCR product with 1 of 2 M28-specific primer pairs. In an isogenic L-A^0^ derivative, this M28-specific PCR product was produced while the 3.0-kb gel band was absent. Killer: killer phenotype of a strain. Individual strain data (Supplementary Tables 3 and 5).

Gel electrophoresis also was used to determine the presence, if at sufficiently high levels, of smaller gel bands, such as M satellites. Eleven L-A^+^ strains had 1 small (1.75–3 kb) gel band (Table 3; Supplementary Table 3; Fig. 1). Seven of these 11 L-A^+^ strains, all of which were Mlus^+^ by PCR, had gel band sizes (2–2.5 kbp) approximately consistent with the previously reported 2.1–2.3 kbp size range of Mlus (Rodriguez-Cousiño *et al.* 2011). One of these 11 L-A^+^ strains (YJM453; PCR: M2^+^) had a small gel band size of 1.75 kbp approximately consistent with M2 (1.5 kbp). The 3 remaining *S. cerevisiae* L-A^+^ strains with a small gel band were YJM1307 (PCR: M1^+^), YJM681 (PCR: M^0^), and YJM195 (PCR: M^0^). The 2.5-kbp gel band in YJM1307 was larger than the 1.6 kb of the canonical M1. The sizes of the 2.5-kbp gel band in YJM681 and the 3.0-kbp gel band in YJM195 were inconsistent with M1, M2, and M28. For these 15 L-A^+^ M^+^ strains, curing L-A cocured M, consistent with their being L-A–dependent M satellites (Table 3; Supplementary Table 3; Fig. 1).

The reference M28-containing K28 killer (MS300c) (Schmitt and Tipper 1990) and the reference Mlus-containing Klus killer (EX229) (Rodriguez-Cousino *et al*. 2011) strains as well as the L-A^+^ M^+^ 100-genomes strains [M1^+^ (*n* = 5); M2^+^ (*n* = 3); Mlus^+^ (*n* = 7); unknown M^+^ (*n* = 2)]; also, for L-A^+^ M1^+^ and L-A^+^ M2^+^, their isogenic L-A^0^ M^0^ derivatives were tested for their abilities to kill 100-genomes strains that were naturally M^0^ (*n* = 83) and isogenic L-A^0^ (M^0^) derivatives (*n* = 28). None of the naturally M^0^ and isogenic L-A^0^ (M^0^) derivatives of the 100-genomes strains were killed by the partial M28 genomic cDNA-containing strain YJM195 (Strope *et al*. 2015b) or by YJM681, the 2 L-A^+^ strains with L-A–dependent, unknown M satellites, or by their isogenic L-A^0^ M^0^ derivatives (Supplementary Table 5 and Supplementary Fig. 3).

**Fig. 2.**
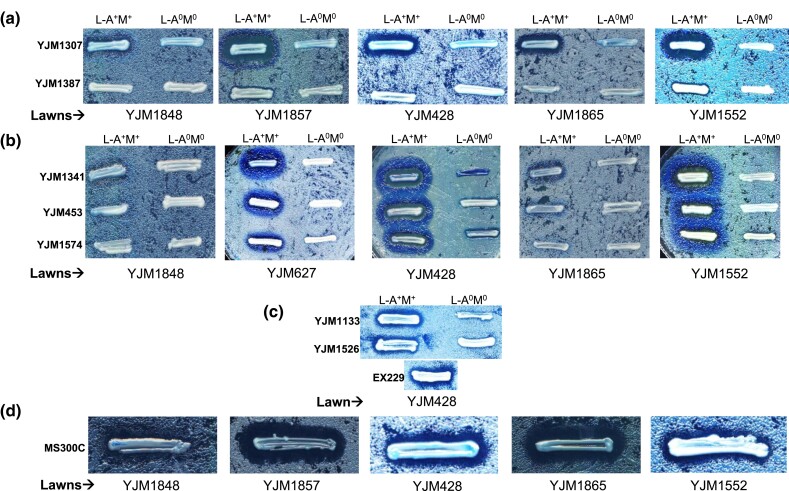
Killer activity in M-containing strains. Isogenic strain pairs of native and L-A–cured strains were tested on lawns of M^0^ strains. Representative strains displaying a) K1, b) K2, c) Klus, and d) K28 killer activity are shown. For Klus, only 1 strain background (YJM428) showed sensitivity to the Mlus toxin. For K28, a corresponding M^0^ strain was not tested.

Of the 100-genomes strains that were naturally M^0^ and isogenic L-A^0^ (M^0^) derivatives, only 1 strain, YJM428 (L-BC^hi^ 20S^+^), was killed by EX229, the reference L-A^+^ Mlus^+^ Klus^+^ strain (Rodriguez-Cousiño *et al.* 2011) (Supplementary Table 5). Of the 100-genomes strains that were L-A^+^ Mlus^+^ (*n* = 7), 4 strains (YJM145, YJM975, YJM978, and YJM993) did not kill YJM428; that is, they were Klus^−^. The 3 remaining L-A^+^ Mlus^+^ strains (YJM320, YJM1133, and YJM1526) killed YJM428; that is, they were Klus^+^ since isogenic L-A^0^ M^0^ derivatives of these 3 strains did not kill YJM428 (Supplementary Table 5; Fig. 2).

In contrast to Klus, many of the 100-genomes strains that were naturally M^0^ and isogenic L-A^0^ (M^0^) derivatives were killed by at least 1 of the 3 L-A^+^ M2^+^ strains (YJM1341, YJM453, and YJM1574); cocuring L-A and M2 eliminated killing. The 3 L-A^+^ M2^+^ K2^+^ strains differed in their K2 killing activity: YJM1341 >YJM453 > YJM1574 (Supplementary Table 5). Like K2, many of the 100-genomes strains that were naturally M^0^ and isogenic L-A^0^ (M^0^) derivatives were killed by at least 3 of the 5 L-A^+^ M1^+^ strains. The 5 L-A^+^ M1^+^ strains differed in their K1 killing activity [YJM1077 = YJM1290 = YJM1307 > YJM1387 (K1^±^) > YJM1419 (K1^−^)]; for the 4 L-A^+^ M1^+^ K1^+^ (or K1^±^) strains, cocuring L-A and M1 eliminated killing (Supplementary Table 5). Finally, many of the 100-genomes strains that were naturally M^0^ and isogenic L-A^0^ (M^0^) derivatives were killed by the reference L-A^+^ M28^+^ K28^+^ strain MS300c (Schmitt and Tipper 1990), including the partial M28 cDNA-containing strain YJM195 (Supplementary Table 5).

The rarity of M satellite–dependent killer strains in the 100-genomes *S. cerevisiae* strains, which is consistent with previous studies (Nakayashiki *et al*. 2005; Pieczynska *et al*. 2013; Chang *et al*. 2015), suggests that some and perhaps all the M satellites have significant fitness costs. Indeed, M1 has a fitness cost (reduced growth rate) in wild-type strains that is alleviated by [Kil-d]-mediated mutation of M1 from K1^+^ to K1^−^ (Suzuki *et al*. 2015). In addition, when present in combination with specific nuclear genome mutations or polymorphisms, M1 and M2 have significant fitness costs (Ball *et al*. 1984; Ridley *et al*. 1984; Edwards *et al*. 2014).

Previous studies have identified strains with M satellite–independent resistance to some killer toxins (Pieczynska *et al*. 2013; Chang *et al*. 2015). Our results suggest that there may be selection for M satellite–independent resistance to killer toxins, with near-complete fixation for Mlus-independent Klus resistance. However, the lack of fixation for M satellite–independent resistance to K1, K2, and K28 toxins suggests that there also may be balancing selection; that is, M satellite–independent resistance to K1, K2, and/or K28 toxins may have fitness costs. M satellite–independent resistance to killer toxins would reduce selection for maintenance of killer toxin-/killer toxin resistance–encoding M satellites that, together with the M-dependent deleterious effects on host fitness and phenotypes referenced above, may contribute to the low frequencies of M satellites in *S. cerevisiae*.

Unlike fully functional K^+^ R^+^ M satellites, mutant M satellites may encode partially functional or nonfunctional killer toxin (K^±^ or K^−^, respectively) and functional killer toxin–specific resistance (R^+^) functions (Talloczy *et al*. 1998, 2000; Suzuki *et al*. 2015). Thus, for YJM1387 (L-A^+^ M1^+^ K1^±^) and YJM1419 (L-A^+^ M1^+^ K1^−^), we compared isogenic L-A^+^ M^+^ vs L-A^0^ M^0^ derivatives from both backgrounds to determine whether these M1 confer resistance to K1 toxin. While parental YJM1387 M1^+^ K1^±^ and YJM1419 M1^+^ K1^−^ strains were resistant to K1 toxin (R1^+^), isogenic L-A^0^ M^0^ strains were sensitive to K1 toxin (R1^−^), consistent with the R1^+^ phenotypes of both L-A^+^ M1^+^ parent strains being M1 dependent.

Like the L-A^+^ M1^+^ K1^−^ R1^+^ strain YJM1419, neither YJM195 nor YJM681, L-A^+^ strains with L-A–dependent, unknown M satellites, killed any of the naturally M^0^ and isogenic L-A^0^ (M^0^) derivatives of the 100-genomes strains (Supplementary Table 5). To test the hypothesis that 1 or both M satellites might be K^−^ R^+^ sequence variants (that is, not detectable by PCR with multiple primer pairs) of known M satellites, we compared the K1, K2, and K28 toxin resistance phenotypes of isogenic L-A^+^ M^+^ vs L-A^0^ M^0^ derivatives of the YJM195 and YJM681 backgrounds. (Neither L-A^+^ M^+^ nor L-A^0^ M^0^ derivatives of the YJM195 and YJM681 backgrounds were killed by Klus^+^ strains; thus, the corresponding Klus experiment was not possible.) In the YJM195 background, the unknown M satellite and/or L-A may confer slight resistance to K1 and K2 killer toxins; however, neither the unknown M satellite nor L-A affected resistance to K28 killer toxin (Supplementary Fig. 2). In the YJM681 background, neither the unknown M satellite nor L-A affected resistance to K1, K2, or K28 killer toxins (Supplementary Fig. 2).

If these L-A–dependent unknown M satellites, like all known M satellites, encode a killer toxin–antitoxin, there are 3 hypotheses for the K^−^ phenotypes of the L-A^+^ M^+^ YJM195 and YJM681 backgrounds. First, the K^−^ phenotypes of 1 or both backgrounds may be [Kil-d]/M satellite genotype ([Bibr jkad167-B73][Bibr jkad167-B72]; Suzuki *et al*. 2015) or nuclear genotype dependent (e.g. *kex1* and *kex2* (Wickner and Leibowitz 1976)). Second, the K^−^ phenotypes of 1 or both backgrounds may be due to the activity of their putative M satellite–encoded killer toxin(s) requiring environmental conditions, such as temperature and/or pH, different from the conditions used to test for the activities of K1, K2, K28, and Klus toxins. Finally, the K^−^ phenotypes of 1 or both parental backgrounds may be due to all the 100-genomes strains being resistant to their putative M satellite–encoded killer toxin(s), like 99 of the 100-genomes strains having Mlus-independent resistance to Klus toxin. Because the ability to detect killer strains depends on both the testing environment (e.g. pH and temperature) and the genotypes of the strains being tested for killing, novel potentially toxin-encoding M satellites may exist in *S. cerevisiae*.

There were significant associations of totiviruses with 2µ plasmid (Strope *et al*. 2015a), as well as with 2 site-specific, mobile mitochondrial introns, *SCE1* and *COX1-intron 1* (Vijayraghavan *et al*. 2019) (Supplementary Table 4). There were also significant associations between the different viruses (Supplementary Table 4). While there were no significant L-A associations with either population or clinical/nonclinical origin, there was significant association of the L-BC level with population (the large wine/European population and mosaic group), as well as significant associations of the L-BC level, 20S and 23S with clinical/nonclinical origin (Supplementary Table 4).

As assayed in M satellite–free 100-genomes strains, there were no significant genotype associations with killer (K1, K2, and K28) toxin resistance phenotypes. Similarly, there were no significant 100-genomes strains genotype associations with the virus (L-A, 20S, and 23S) presence or with the L-BC level (data not shown), with the exceptions of the abovementioned *SCE1* and *COX1-intron 1*. The lack of significant genotype associations may be due to small population size (*n* = 100), population structure, and/or complex genetic architecture. In addition, the lack of significant host genotype associations with the L-BC level also may be confounded by the significant L-BC genotype–L-BC level association (Table 2; Supplementary Table 3).

### Phenotypic effects of L-A

We recently described a novel narnavirus, N1199, that, when present in a high copy number, has major effects on *S. cerevisiae* host phenotypes (Vijayraghavan *et al*. 2023). However, despite extensive characterization (e.g. ([Bibr jkad167-B81][Bibr jkad167-B82], 1996b; Wickner *et al*. 2013)), to our knowledge, the L-BC, 20S, and 23S RNA viruses have no previously described effects on *S. cerevisiae* phenotypes and the L-A virus has few previously described effects on *S. cerevisiae* host phenotypes (Dihanich *et al*. 1987, 1989; Liu and Dieckmann 1989; Gao *et al*. 2019; Chau *et al*. 2023). The near complete absence of previously described RNA virus effects on *S. cerevisiae* host phenotypes stands in sharp contrast to the many effects of RNA viruses, including dsRNA totiviruses, on host phenotypes in other species of fungi (McCabe *et al*. 1999; Dawe and Nuss 2001; Nuss 2005; Ghabrial and Suzuki 2009; Bhatti *et al*. 2011; Xie and Jiang 2014; Ghabrial *et al*. 2015). However, in our analysis, there were no virus or M satellite associations with 100-genomes strain nonkiller phenotypes (listed in Supplementary Table 4); hypotheses for the lack of association include the population size, population structure, choice of tested phenotypes, host genotype–virus epistasis, and RNA virus genotype variation.

**Fig. 3.**
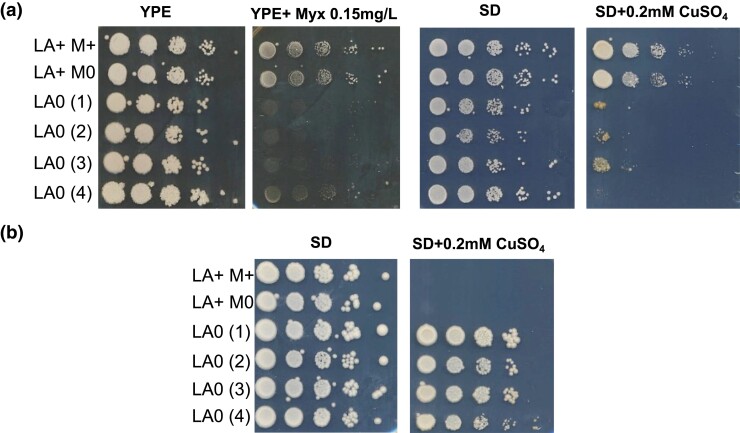
Phenotypic analysis of a) YJM1133 and b) YJM1419 L-A^+^ M^+^ parental as well as isogenic L-A^+^ M^0^ and L-A^0^ M^0^ derivatives. Ten-fold dilutions of isogenic strains differing only in the presence/absence of L-A and/or M were spotted on media with the indicated compounds and monitored for growth.

An independent method to identify virus-dependent phenotypes is to compare the phenotypes of isogenic virus-containing and virus-free strain sets, which we did for L-A. For the 28 L-A^+^ parental genetic backgrounds where L-A could be cured, we compared the phenotypes (Strope *et al*. 2015b; Vijayraghavan *et al*. 2019) of isogenic L-A^+^ and L-A^0^ strain sets. In 2 of these 28 genetic backgrounds [YJM1133 (parental): L-A^+^ Mlus^+^ Klus^+^; YJM1419 (parental): L-A^+^ M1^+^ K1^−^], there were differences in copper (YJM1133, YJM1419) and myxothiazol (a cytochrome b oxidase inhibitor; YJM1133) resistance phenotypes between the parental L-A^+^ M^+^ strains and multiple independently cured, isogenic L-A^0^ M^0^ derivatives (Fig. 3). Because these L-A^+^ M^+^ parent vs L-A^0^ M^0^ derivative comparisons could not distinguish between L-A– and M-dependent phenotypes, YJM1133 and YJM1419 background strains were cured of their M satellites to generate L-A^+^ M^0^ derivatives. (Gel and PCR analyses of parental and representative cured derivatives of both backgrounds are shown in Supplementary Fig. 4.) Upon testing, parental YJM1133 and YJM1419 background L-A^+^ M^+^ strains and isogenic L-A^+^ M^0^ strains had the same copper and myxothiazol resistance phenotypes, which differed from the phenotypes of isogenic L-A^0^ M^0^ strains (Fig. 3), consistent with these phenotypes being L-A dependent.

Our previous 100-genomes strains study identified the *CUP1* locus, where the copy number ranges from 1 to 18 tandem copies, as the major effect contributor to copper resistance (Strope *et al*. 2015b). The YJM1419 (*CUP1*: *n* = 4) and YJM1133 (*CUP1*: *n* = 2) backgrounds are at the low end of the *CUP1* copy number range, which may have facilitated the identification of L-A–dependent effects on copper resistance. Interestingly, while the copper resistance phenotypes in the YJM1133 and YJM1419 backgrounds were L-A dependent, the phenotypic effect of L-A was opposite in the 2 backgrounds. One hypothesis for the opposite effects of L-A on copper resistance in the YJM1419 and YJM1133 backgrounds is that they are L-A genotype dependent; however, while not definitive, the L-A in YJM1419 and YJM1133 have the same PCR genotypes (Supplementary Table 3). A second hypothesis for the opposite effects of L-A on copper resistance in the YJM1419 and YJM1133 backgrounds is that they are host genotype dependent. In either case, unless accounted for, L-A is a confounding factor for studies of copper resistance as well as, at a minimum, myxothiazol resistance. More generally, RNA viruses have the potential to be confounding factors for multiple host phenotypes.

### Chromosomal killer genes and killer phenotypes

Some yeast killer toxin–encoding genes are not in viruses, or virus satellites, but are instead chromosomally encoded (Magliani *et al*. 1997; Liu *et al*. 2015). Thus, we complemented our analysis of M satellite–encoded killer toxins/antitoxins and M satellite–independent resistance to M satellite–encoded killer toxins by analyzing chromosomal killer genes and killer phenotypes in the 100-genomes strains. In *S. cerevisiae*, although less studied than M satellite–dependent killers, 2 chromosomally encoded killer genes have been described, *KHR1* on chromosome 9 (Goto *et al*. 1990b) and *KHS1* on chromosome 5 (Goto *et al*. 1991). We screened the 100-genomes sequences for *KHR1* (Goto *et al*. 1990a, 1990b). The *KHR1* ORF was not present in 31 strains, including the canonical S288c genome, and was present in 69 of the 100-genomes strains, 1 of which (YJM1326) had a premature stop codon polymorphism (Supplementary Table 6). Similarly, we screened the 100-genomes sequences for *KHS1* (Goto *et al*. 1991), using the *KHS1* ORF sequence from YJM789 (Wei *et al*. 2007) because, as noted by Frank and Wolfe (Frank and Wolfe 2009), the previously determined *KHS1* sequence (Goto *et al*. 1991) was likely misassembled. While absent from 1 strain (YJM326), *KHS1* was present in 99 of the 100-genomes strains. Of the 99 *KHS1*-containing strains, 46 had *S. cerevisiae KHS1* and 53 had *KHS1* introgressed from *S. paradoxus*. Of the 46 strains with *S. cerevisiae KHS1*, 9 had full-length ORFs and 35 strains, including the canonical S288c, had a premature stop codon polymorphism (W66 to opal) with a further 2 strains also having a second premature stop codon polymorphism (Y88 to ochre) (Supplementary Table 6). Our *KHS1* premature stop codon polymorphism and introgression results are consistent with previous analysis of *KHS1* (Frank and Wolfe 2009), and both are consistent with *khs1* being present and misannotated in the canonical S288c genome.

As a first step in assessing chromosomal killers, we tested L-A^+^ M^+^ and isogenic L-A^0^ M^0^ derivatives of the 100-genomes strains for their abilities to kill *C. glabrata* ATCC 2001, the same strain used to isolate and characterize the *S. cerevisiae* chromosomal killer genes *KHR1* (Goto *et al*. 1990a, 1990b) and *KHS1* (Goto *et al*. 1991). In contrast to their loss of *S. cerevisiae* killing activity (Supplementary Table 5), many M^0^ derivatives retained at least some of their *C. glabrata* killing activity (Supplementary Table 6), consistent with the presence in many *S. cerevisiae* strains of functional chromosomal killer gene(s). Thus, to eliminate confounding by M satellite–dependent killing, we utilized M satellite–free 100-genomes *S. cerevisiae* strains to characterize chromosomal killer genes and phenotypes for all subsequent experiments.

None of the M satellite–free 100-genomes *S. cerevisiae* strains killed *C. glabrata* ATCC 2001 under conditions specific for Khr1-dependent killing (Goto *et al*. 1990a, 1990b) (Supplementary Table 6), an example of which is shown (Supplementary Fig. 6a). Thus, *KHR1* is not further described or discussed. In contrast, 70 of the M satellite–free and L-A– and M satellite–cocured derivatives of the 100-genomes strains killed *C. glabrata* ATCC 2001 to varying degrees under conditions specific for Khs1-dependent killing (Goto *et al*. 1991) (Supplementary Table 6). Testing for associations between killing of *C. glabrata* and *S. cerevisiae* genotypes showed the sole significant association to be a peak on the right arm of chromosome 5, including YER187w (Supplementary Fig. 7 and Supplementary Table 7) that corresponds to the 3′ region of the *KHS1* ORF, as identified by (Frank and Wolfe 2009).

Similarly, we used YJM189, which has the strongest Khs1*C. glabrata* killing phenotype (Supplementary Table 6), to test for killing of the M satellite–free 100-genomes strains and, subsequently, association with *S. cerevisiae* genotypes and again found the sole significant association corresponded to *KHS1* (Supplementary Fig. 8 and Supplementary Table 8). However, relative to the association between M satellite–free killing of *C. glabrata* and *S. cerevisiae* genotypes, the YJM189 *KHS1* killing of the M satellite–free 100-genomes strains association was not as clear-cut, for which we suggest a hypothesis. *S. cerevisiae* strains have an unavoidable ancestral history of exposure to *S. cerevisiae* killer toxins, with the resulting selective pressure likely affecting sensitivity to these toxins, possibly analogous to the M satellite–independent resistance to M satellite–encoded toxins described above. In contrast to *S. cerevisiae*, its close relative *C. glabrata* (Roetzer *et al*. 2011; Gabaldón *et al.* 2013) may be viewed as a relative blank slate with respect to ancestral history of exposure to *S. cerevisiae* killer toxins that may affect sensitivity to *S. cerevisiae* killer toxins; indeed, this may have been an unstated rationale for the previous use of *C. glabrata* ATCC 2001 to isolate and characterize the *S. cerevisiae* chromosomal killer genes *KHR1* (Goto *et al*. 1990a, 1990b) and *KHS1* (Goto *et al*. 1991). Thus, while unlikely to be the sole explanation, 1 hypothesis is that heterogeneity in the 100-genomes strains for Khs1 antitoxin–independent resistance to Khs1 toxin contributes to the less clear-cut *KHS1* association. Evidence consistent with Khs1 antitoxin–independent resistance to Khs1 toxin is described below.

We tested the *KHS1* genotype association by deleting *KHS1* in YJM189 and found that the isogenic *khs1*Δ strain YJM1896 did not kill *C. glabrata* (Supplementary Fig. 6a) (Supplementary Table 6) or Khs1-sensitive *S. cerevisiae* strains (Supplementary Table 6). Thus, both the killing of *C. glabrata* and Khs1-sensitive *S. cerevisiae* strains was entirely *KHS1* dependent, at least for the YJM189 *KHS1* genetic background. Because M satellite–derived killer toxin(s) require processing by host-encoded killer expression protease(s) for their activity (Wickner and Leibowitz 1976; Wickner 1992; Wickner *et al*. 2013), we tested *KEX1* and *KEX2* for their roles in Khs1 killer activity. While *kex1*Δ had no effect, *kex2*Δ eliminated Khs1 killer activity in the *KHS1* YJM189 background (Supplementary Fig. 6b). Because each of the M satellites encodes a toxin–antitoxin, we tested the sensitivity of the *khs1*Δ strain YJM1896 to killing by the isogenic *KHS1* strain YJM189; the *khs1*Δ strain YJM1896 was sensitive to exogenous Khs1 toxin from the isogenic *KHS1* parent strain (Supplementary Fig. 6c). That is, like the M satellite–encoded toxins–antitoxins, *KHS1* encodes both Khs1 toxin and Khs1 antitoxin, with resistance to exogenous Khs1 toxin being dependent on endogenous Khs1 antitoxin, at least in the YJM189 genetic background.

While the killing of *C. glabrata*/resistance to killing by YJM189 *KHS1* phenotypes of most of the 100-genomes strains were consistent with their *KHS1* genotypes, the phenotypes of some strains were inconsistent, including 17 of the 34 strains having the *khs1* single premature opal stop codon polymorphism. One hypothesis for the phenotypic inconsistencies of these 17 *khs1* opal strains would be translational read-through that should result in the production of both Khs1 toxin and antitoxin. However, these 17 *khs1* opal strains had phenotype(s) inconsistent with such translational read-through (see below).

The choice of YJM189 for phenotypic testing was serendipitous as this genetic background allowed both *KHS1*-dependent and *KHS1*-independent killer phenotypes, as well as *KHS1*-independent Khs1 toxin resistance phenotypes, to be identified, as described below. Based on *KHS1* genotypes and killing of *C. glabrata*/resistance to killing by YJM189 *KHS1* phenotypes, classes are described and hypotheses (other than opal translational read-through) for inconsistent phenotypes are discussed below.

Class 1 strains (*n* = 20), which did not kill *C. glabrata* and were killed by YJM189 *KHS1*, consisted of 17 strains with the *khs1* opal polymorphism, 2 strains with 2 *khs1* premature stop codons (66 opal, 88 ochre), and YJM326, the sole *khs1* null (not present in genome) strain. The phenotypes of all class 1 strains were consistent with their presumed inability to produce Khs1 toxin and Khs1 antitoxin.

Class 2 strains (*n* = 10), which did not kill *C. glabrata* but were resistant to killing by YJM189 *KHS1*, consisted of 10 strains with the *khs1* opal polymorphism. Like class 1 strains, the inability to kill the *C. glabrata* phenotype of class 2 strains was consistent with their presumed inability to produce Khs1 toxin. However, in contrast to class 1 strains, the resistance to killing by YJM189 *KHS1* of class 2 strains was inconsistent with their presumed inability to produce Khs1 antitoxin. The resistance of the *khs1* class 2 strains to killing by YJM189 *KHS1* is hypothesized to be due to *KHS1*-independent resistance to exogenous Khs1 toxin, like the M satellite–independent resistance to each of the M satellite–encoded killer toxins described above.

Class 3 strains (*n* = 60), which killed *C. glabrata* and were resistant to killing by YJM189 *KHS1*, consisted of 58 strains with full-length *KHS1* ORFs (introgressed *S. paradoxus KHS1*: *n* = 52; *S. cerevisiae KHS1*: *n* = 6) and 2 strains with the *khs1* opal polymorphism. Based on *KHS1* genotypes, class 3 strains were split into 2 subclasses. For class 3A strains with full-length *KHS1* ORFs (*n* = 58), both the *C. glabrata* and YJM189 phenotypes were consistent with their presumed production of functional Khs1 toxin and Khs1 antitoxin. For class 3B strains with the *khs1* opal polymorphism (*n* = 2), both the *C. glabrata* phenotypes and the YJM189 phenotypes were inconsistent with nonfunctional *khs1* genotypes. The resistance to killing by the YJM189 *KHS1* phenotype of the 2 *khs1* class 3B strains is hypothesized to be due to *KHS1*-independent resistance to exogenous Khs1 toxin, as in class 2 strains. The *C. glabrata* killing phenotype of the 2 *khs1* class 3B strains (YJM1383 and YJM1450) is hypothesized to be due to their production of a non-Khs1 toxin. This hypothesized non-Khs1 toxin is not Khr1 because, like all the 100-genomes strains, neither strain shows Khr1 killer activity; in addition, YJM1383 has no *KHR1* in its genome (Supplementary Table 6). The hypothesized non-Khs1 toxin may be antimicrobial glyceraldehyde-3-phosphate dehydrogenase–derived peptides that have been shown to kill some bacteria and some non-*Saccharomyces* yeasts (Branco *et al*. 2014, 2017, 2018, 2019).

Finally, class 4 strains (*n* = 10), which killed *C. glabrata* and were killed by YJM189 *KHS1*, consisted of 2 strains with the introgressed *S. paradoxus KHS1* ORF, 3 strains with the full-length *S. cerevisiae KHS1* ORF, and 5 strains with the *khs1* opal polymorphism. Based on *KHS1* genotypes, the class 4 strains were split into 2 subclasses. For class 4A strains with the *khs1* opal polymorphism (*n* = 5), while the *C. glabrata* phenotypes were inconsistent, the YJM189 phenotypes were consistent with nonfunctional *khs1* genotypes. The *C. glabrata* killing phenotype of class 4A *khs1* strains is hypothesized to be due to their production of a non-Khs1 toxin, as in the class 3B strains. For class 4B strains with full-length *KHS1* ORFs (*n* = 5), while the *C. glabrata* killing phenotypes were consistent, those killed by YJM189 phenotypes were inconsistent with those by functional *KHS1* genotypes. Those killed by the YJM189 phenotype of class 4B strains is hypothesized to be due to *KHS1* being functionally null, possibly due to *cis*-acting *KHS1* noncoding polymorphisms or *trans*-acting polymorphisms in transcription factor(s) and/or by silencing of the subtelomeric *KHS1* gene. The killing of the *C. glabrata* phenotype of class 4B strains is hypothesized to be due to their production of a non-Khs1 toxin, as in class 3B and class 4A strains.

Thus, *S. cerevisiae* can produce multiple toxins and antitoxins, both L-A–dependent M satellite–encoded and chromosomally encoded, as well as chromosomally encoded, presumably non–antitoxin-mediated toxin resistance mechanisms, each of which exhibits variation in the 100-genomes strains. Like the variation in M satellite–independent resistance to K1, K2, and K28 toxins described above, variation in Khs1 production and for Khs1 antitoxin–independent resistance to exogenous Khs1 toxin, as well as for variation for production of non-Khs1 toxin(s), suggests balancing selection.

## Conclusion

When present in an *S. cerevisiae* strain, a functional killer toxin–antitoxin–encoding gene, whether chromosomally or M satellite encoded, can be antimicrobial, killing strains that lack that gene, with the caveat that such killing is affected by genotype (i.e. antitoxin-independent effects on killer toxin resistance) and by the environment. Thus, secreted killer toxin–encoding genes may have fitness benefits via their antimicrobial activities in naturally occurring mixed genotype/heterogeneous cultures but may be confounding in mixed genotype/heterogeneous culture experiments. These same killer toxin–encoding genes also may have deleterious effects on fitness, as has been shown for the L-A–dependent M1 satellite that confers a severe growth defect or lethality when present in specific genetic backgrounds (Ridley *et al*. 1984; Sommer and Wickner 1987; Edwards *et al*. 2014). Indeed, even in backgrounds where it does not confer a severe growth defect or lethality, M1 confers a slight growth deficit (Suzuki *et al*. 2015). Notably, rather than mixed genotype/heterogeneous cultures, the cited cases are in clonal/uniform cultures, which may suggest an intracellular/cell autonomous phenotypic effect of M1/K1; other M satellites may have similarly deleterious effects on host fitness. Given the abundance of *khs1* alleles, *KHS1* also may have deleterious effects on fitness. We suggest that killer toxin–encoding M satellites and *KHS1* may confound the analysis of host phenotypes, including quantitative traits.

As with genotype variation in nuclear genes that often affects organismal phenotypes, genotype variation in viruses can affect viral phenotypes. For example, although the mechanism(s) and phenotypically relevant sequence difference(s) are not known, genotypically distinct L-A viruses differ in their abilities to compete with each other, to resist curing, and to support different M satellites (Wickner 1980; Wickner and Toh-e 1982; Wickner 1983, 1992; Rodriguez-Cousino *et al*. 2013, 2017). RNA viral genotype–dependent effects on *S. cerevisiae* host phenotypes, if such exist, could contribute to the lack of virus presence/absence associations with host phenotypes. Similarly, high-frequency RNA viral mutation that improves viral adaptation to host genotypes could also contribute to the lack of virus presence/absence associations with host phenotypes, as further discussed below.

Like M-dependent effects on host phenotypes that are only seen in *ski* mutant strains with elevated levels of M (Ridley *et al*. 1984; Sommer and Wickner 1987), RNA virus–dependent effects on host phenotypes may also depend on RNA virus levels. RNA virus levels may be environment dependent, as has been reported for 20S and 23S (Garvik and Haber 1978; Wesolowski and Wickner 1984; Matsumoto *et al*. 1990) and L-A (Oliver *et al*. 1977). RNA virus levels also may be host genotype dependent, as has been reported for L-A in *por1* (Dihanich *et al*. 1987; Dihanich *et al*. 1989), *nuc1* (Liu and Dieckmann 1989), *lcb2* (Garnepudi *et al*. 1997), and *ski* mutants (Wickner 1992, 1996a). Given the high mutation frequencies of RNA viruses in other species (Sanjuan *et al*. 2010), mutations in *S. cerevisiae* RNA viruses also may affect virus levels. Interestingly, the reported increased L-A level in *por1* mutants was accompanied, after a lag, by L-A–dependent suppression of the *por1* respiration defect (Dihanich *et al*. 1987, 1989). In principle, the increased L-A levels may be due to secondary host mutation(s) and/or to L-A mutation(s). We suggest that RNA virus levels may contribute to RNA virus–dependent effects on host phenotypes.

Of the 100-genomes strains, 67 were L-BC^lo^ and 70 were L-A^0^. Of the 30 L-A^+^ 100-genomes strains, while 17 were M^+^, only 10 were K^+^. In contrast, 5 *S. cerevisiae* genetic backgrounds commonly used in the laboratory are quite distinct: S288c (isogenic with YJM1552) is L-A^+^ L-BC^hi^ M^0^; RM11 (isogenic with YJM1293) is L-A^0^ L-BC^hi^ M^0^; Σ1278b (isogenic with YJM1290) is L-A^+^ L-BC^hi^ M1^+^ K1^+^; SK1 (isogenic with YJM1077) is L-A^+^ L-BC^hi^ M1^+^ K1^+^; and YJM789 (isogenic with YJM145) is L-A^+^ L-BC^hi^ Mlus^+^ Klus^−^. Clearly, these commonly used genetic backgrounds are not representative for L-BC level, L-A, L-A–dependent M satellites, or M satellite–dependent killer phenotypes in *S. cerevisiae*. To our knowledge, the only study that has examined interactions between the host and viral (or, in this case, M1 satellite) genomes found profound effects (Edwards *et al*. 2014). In addition to M satellites, we suggest that L-A and, possibly, L-BC may be confounding factors in the analysis of both qualitative and quantitative traits.

In numerous species, RNA viruses have very high mutation rates (Sanjuan *et al*. 2010). Thus, the identification of RNA virus and M satellite effects on *S. cerevisiae* host phenotypes may be confounded by high rates of RNA virus and M satellite mutation and loss that are likely to be far higher than *S. cerevisiae* host mutation. While the mutation rates for *S. cerevisiae* RNA viruses are not known, there is strong evidence that the mutation/loss rates for M satellites are high due, for example, to the prion-like [KIL-d] element (Talloczy *et al*. 1998, 2000; Suzuki *et al*. 2015). Mutation of RNA viruses and L-A–dependent M satellites may result in their loss, which would result in virus- and M satellite–dependent effects on host phenotypes also being lost. Alternatively, mutation of RNA viruses and L-A–dependent dsRNA M satellites may result in virus and M satellite adaptation to environment(s) and/or to *S. cerevisiae* host genotype(s). Such RNA viral and M satellite adaptation may result in the modification of viral- and M satellite–dependent effects on host phenotypes. In sum, the presence of RNA viruses and M satellites varied considerably across the 100-genomes population resource. Virus-dependent and M satellite–dependent phenotypes were characterized and extend our understanding of the phenotypic effects of these non–Mendelian-inherited factors. Given their effects on host phenotype and the potential for virus/satellite mutation and loss, the contributions of RNA viruses and L-A–dependent M satellites should be considered in *S. cerevisiae* studies.

## Supplementary Material

jkad167_Supplementary_Data

## Data Availability

The 100-genomes strains (and derivatives) and plasmids described in this work have been deposited in Fungal Genetics Stock Center (http://www.fgsc.net) and Addgene (http://www.addgene.org/John_McCusker/), respectively. Supplemental material available at G3 online.
