# Supplementary material for: RNA viruses, M satellites, chromosomal killer genes, and killer/nonkiller phenotypes in the 100-genomes S. cerevisiae strains
Source: G3 (Bethesda). 2023 Jul 27;13(10):jkad167. doi: 10.1093/g3journal/jkad167 (PMC10542562; doi:10.1093/g3journal/jkad167)
Supplement: jkad167_Supplementary_Data [file jkad167_supplementary_data.zip › Figure_S1_G3-2023-404116.pdf]

# Figure S1



***Start*** (0)

```
5′ GAAAAATTTTTAAATTCATATAACTCCCCATGCTAAGATTTGTTA
                                                  45
3′ CTTTTTAAAAATTTAAGTATATTGAGGGGTACGATTCTAAACAAT

   CTAAAAACTCTCAAGATAAATCGTCTGATCTATTCTCTATTTGTT
                                                  90
   GATTTTTGAGAGTTCTATTTAGCAGACTAGATAAGAGATAAACAA

   CTGATCGCGGAACTTTTGTTGCCCATAATAGAGTTCGGACTGATT
                                                  135
   GACTAGCGCCTTGAAAACAACGGGTATTATCTCAAGCCTGACTAA

   TCAAGTTTGACAACTTAGTATTCAACCGAGTTTATGGTGTTTCTC
                                                  180
   AGTTCAAACTGTTGAATCATAAGTTGGCTCAAATACCACAAAGAG

   AAAAATTTACGTTAGTCGGTAACCCGACAGTCTGCTTTAATGAAG
                                                  225
   TTTTTAAATGCAATCAGCCATTGGGCTGTCAGACGAAATTACTTC

   GCAGTTCTTACCTAGAAGGTATTGCTAAAAAGTACCTAACTTTGG
                                                  270
   CGTCAAGAATGGATCTTCCATAACGATTTTTCATGGATTGAAACC

   ATGGAGGACTTGCCATTGACAATGTCCTCAATGAGTTGAGATCCA
                                                  315
   TACCTCCTGAACGGTAACTGTTACAGGAGTTACTCAACTCTAGGT

   CTTGCGGTATCCCAGGTAATGCTGTTGCATCTCATGCGTATAATA
                                                  360
   GAACGCCATAGGGTCCATTACGACAACGTAGAGTACGCATATTAT

   TTACATCCTGGCGTTGGTATGACAATCACGTGGCACTGTTGATGA
                                                  405
   AATGTAGGACCGCAACCATACTGTTAGTGCACCGTGACAACTACT

   ACATGTTGCGTGCTTACCACTTACAAGTATTGACCGAACAGGGCC
                                                  450
   TGTACAACGCACGAATGGTGAATGTTCATAACTGGCTTGTCCCGG

   AATATAGCGCTGGAGATATCCCTATGTACCATGATGGACATGTCA
                                                  495
   TTATATCGCGACCTCTATAGGGATACATGGTACTACCTGTACAGT
```

```
AAATCAAGCTACCAGTGACTATCGATGACACGGCAGGCCCAACAC   540
TTTAGTTCGATGGTCACTGATAGCTACTGTGCCGTCCGGGTTGTG

AATTCGCTTGGCCTAGTGACAGGTCTACTGATTCGTATCCTGATT   585
TTAAGCGAACCGGATCACTGTCCAGATGACTAAGCATAGGACTAA

GGGCACAGTTTTCTGAATCATTTCCATCAATCGACGTCCCGTACC   630
CCCGTGTCAAAAGACTTAGTAAAGGTAGTTAGCTGCAGGGCATGG

TAGATGTTAGGCCATTGACCGTAACGGAAGTCAATTTCGTGCTTA   675
ATCTACAATCCGGTAACTGGCATTGCCTTCAGTTAAAGCACGAAT

TGATGATGAGTAAGTGGCATAGACGTACTAACTTAGCGATAGACT   720
ACTACTACTCATTCACCGTATCTGCATGATTGAATCGCTATCTGA

ACGAGGCACCCCAACTAGCTGATAAGTTCGCTTACCGCCATGCGC   765
TGCTCCGTGGGGTTGATCGACTATTCAAGCGAATGGCGGTACGCG
                                         CGCG
                                         LA-R3

TTACTGTTCAAGACGCTGACGAGTGGATAGAAGGCGATAGAACTG   810
AATGACAAGTTCTGCGACTGCTCACCTATCTTCCGCTATCTTGAC
AATGACAAGTTCTGCG
LA-R3

ATGACCAGTTCCGCCCCCCCTCGTCTAAAGTAATGTTATCGGCAC   855
TACTGGTCAAGGCGGGGGGGAGCAGATTTCATTACAATAGCCGTG

TTCGTAAGTACGTGAACCATAACAGGCTGTACAATCAGTTTTACA   900
AAGCATTCATGCACTTGGTATTGTCCGACATGTTAGTCAAAATGT

CTGCAGCACAACTGTTAGCTCAAATTATGATGAAACCTGTCCCTA   945
GACGTCGTGTTGACAATCGAGTTTAATACTACTTTGGACAGGGAT
```

```
ACTGCGCTGAGGGCTACGCTTGGCTGATGCATGACGCATTGGTCA
                                                    990
TGACGCGACTCCCGATGCGAACCGACTACGTACTGCGTAACCAGT

ATATACCAAAATTTGGGTCTATTCGAGGAAGGTACCCCTTTTTGT
                                                    1035
TATATGGTTTTAAACCCAGATAAGCTCCTTCCATGGGGAAAAACA

TATCAGGTGATGCAGCGTTGATTCAGGCTACAGCCCTAGAAGACT
                                                    1080
ATAGTCCACTACGTCGCAACTAAGTCCGATGTCGGGATCTTCTGA

GGTCTGCTATCATGGCGAAACCCGAGCTGGTGTTCACTTACGCGA
                                                    1125
CCAGACGATAGTACCGCTTTGGGCTCGACCACAAGTGAATGCGCT

TGCAGGTGTCAGTAGCGTTAAACACCGGACTATACTTACGTCGCG
                                                    1170
ACGTCCACAGTCATCGCAATTTGTGGCCTGATATGAATGCAGCGC

TTAAGAAAACAGGCTTCGGCACAACTATAGATGACAGCTATGAAG
                                                    1215
AATTCTTTTGTCCGAAGCCGTGTTGATATCTACTGTCGATACTTC

ATGGAGCGTTTTTGCAACCGGAGACGTTCGTTCAGGCCGCACTAG
                                                    1260
TACCTCGCAAAAACGTTGGCCTCTGCAAGCAAGTCCGGCGTGATC

CATGTTGTACCGGACAAGATGCGCCCCTAAATGGGATGTCAGATG
                                                    1305
GTACAACATGGCCTGTTCTACGCGGGGATTTACCCTACAGTCTAC

TGTATGTCACTTATCCAGATCTTCTAGAATTTGATGCTGTTACAC
                                                    1350
ACATACAGTGAATAGGTCTAGAAGATCTTAAACTACGACAATGTG

AAGTACCCATCACGGTCATTGAGCCCGCTGGCTATAACATTGTTG
                                                    1395
TTCATGGGTAGTGCCAGTAACTCGGGCGACCGATATTGTAACAAC

ATGATCATTTAGTGGTTGTGGGTGTACCTGTGGCATGTTCACCAT
                                                    1440
TACTAGTAAATCACCAACACCCACATGGACACCGTACAAGTGGTA
```

ACATGATATTTCCAGTAGCTGCGTTTGATACTGCAAATCCTTACT
TGTACTATAAAGGTCATCGACGCAAACTATGACGTTTAGGAATGA 1485

GTGGGAATTTTGTCATTAAGGCTGCTAACAAGTATCTCCGTAAGG
CACCCTTAAAACAGTAATTCCGACGATTGTTCATAGAGGCATTCC 1530

GTGCCGTGTATGATAAACTCGAAGCATGGAAGTTGGCCTGGGCAC
CACGGCACATACTATTTGAGCTTCGTACCTTCAACCGGACCCGTG 1575

TGAGGGTAGCCGGGTATGACACTCACTTCAAAGTGTATGGCGATA
ACTCCCATCGGCCCATACTGTGAGTGAAGTTTCACATACCGCTAT 1620

CACACGGCTTAACTAAGTTCTATGCTGACAACGGTGACACATGGA
GTGTGCCGAATTGATTCAAGATACGACTGTTGCCACTGTGTACCT 1665

CACACATACCTGAATTTGTCACTGACGGTGACGTGATGGAAGTAT
GTGTGTATGGACTTAAACAGTGACTGCCACTGCACTACCTTCATA 1710

TCGTTACTGCCATCGAACGCAGAGCTAGACATTTCGTTGAACTAC
AGCAATGACGGTAGCTTGCGTCTCGATCTGTAAAGCAACTTGATG 1755

CTAGACTGAATTCACCAGCATTCTTCAGATCTGTAGAAGTCAGCA
GATCTGACTTAAGTGGTCGTAAGAAGTCTAGACATCTTCAGTCGT 1800

CCACTATATATGATACTCATGTGCAGGCTGGTGCGCATGCGGTGT
GGTGATATATACTATGAGTACACGTCCGACCACGCGTACGCCACA 1845

ATCATGCTAGTCGAATCAATCTTGATTATGTTAAGCCTGTTTCGA
TAGTACGATCAGCTTAGTTAGAACTAATACAATTCGGACAAAGCT 1890

CCGGCATTCAGGTGATCAATGCGGGCGAACTTAAGAACTACTGGG
GGCCGTAAGTCCACTAGTTACGCCCGCTTGAATTCTTGATGACCC 1935

GTAGTGTGCGTCGTACTCAGCAGGGTTTAGGAGTGGTAGGTCTTA 1980
CATCACACGCAGCATGAGTCGTCCCAAATCCTCACCATCCAGAAT

CGATGCCAGCTGTAATGCCTACCGGAGAACCTACAGCTGGCGCTG 2025
GCTACGGTCGACATTACGGATGGCCTCTTGGATGTCGACCGCGAC

CCCACGAAGAGTTGATAGAACAGGCGGACAATGTTTTAGTAGAGT 2070
GGGTGCTTCTCAACTATCTTGTCCGCCTGTTACAAAATCATCTCA

AAACGTAATCGAACCCTCACACGGACCCCGCCCTACAAGGTACAT 2115
TTTGCATTAGCTTGGGAGTGTGCCTGGGGCGGGATGTTCCATGTA

ACTGCAGGAACCAGGTACGTACCCTGCGTGGATTAGATTCAGGAA 2160
TGACGTCCTTGGTCCATGCATGGGACGCACCTAATCTAAGTCCTT

CAGAGTACAAGCTGTATCGCGTCAGAAAGCCACTCACTTCTTGTT 2205
GTCTCATGTTCGACATAGCGCAGTCTTTCGGTGAGTGAAGAACAA

TGACATCGTACCTGCCGCAGTAATTAGTGATTTTACTACGTCTGA 2250
ACTGTAGCATGGACGGCGTCATTAATCACTAAAATGATGCAGACT

CACGTCTTCGTTTGCATACAAATCGCACACCTACGCTGTAAATGT 2295
GTGCAGAAGCAAACGTATGTTTAGCGTGTGGATGCGACATTTACA

AACAGCATTGAGGTTCAGTGACACTTATGCCTTGTACGTACAGAC 2340
TTGTCGTAACTCCAAGTCACTGTGAATACGGAACATGCATGTCTG

TGATACCAACATGACAATTTTAAGCCCAGCGGCGCGTCGCCAGGC 2385
ACTATGGTTGTACTGTTAAAATTCGGGTCGCCGCGCAGCGGTCCG

TTCTGCGACGTACTCACAGGTGGCAGGGTTTGTTATAACACACC 2430
AAGACGCTGCATGAGTGTCCACCGTCCCAAAACAATATTGTGTGG

TACCGTTATGGATTCGCTAGCGAATATCTTGGACGTAGACCGCAA 2475
ATGGCAATACCTAAGCGATCGCTTATAGAACCTGCATCTGGCGTT

TATACGACCCAAACACTTCAAGGGTTTACGGCTATACACAAGGTC 2520
ATATGCTGGGTTTGTGAAGTTCCCAAATGCCGATATGTGTTCCAG

TAAGGTCACTGCTCAACATCATACTCACTTGCGGCCAGACGAGCT 2565
ATTCCAGTGACGAGTTGTAGTATGAGTGAACGCCGGTCTGCTCGA

AGTGGAAGCGGCCGCAAAGGTCTCGCCTAGACGTAAATACTACTT 2610
TCACCTTCGCCGGCGTTTCCAGAGCGGATCTGCATTTATGATGAA

AATGTGTGTAGTTGAGCTGCTCGCGAACTTACAAGTAGATCTTGA 2655
TTACACACATCAACTCGACGAGCGCTTGAATGTTCATCTAGAACT

AGCAGCAGTAGCTACTATTCTCGCATATGTCCTGACACTAAGTGA 2700
TCGTCGTCATCGATGATAAGAGCGTATACAGGACTGTGATTCACT

AAAATTTGTACCAATTTTCTTGGATTCTAGAGCAATATGGGTCGG 2745
TTTTAAACATGGTTAAAAGAACCTAAGATCTCGTTATACCCAGCC

TGAGCCTGGGCCTGATGCTCTGACTGCACGTCTCAAGGCCAGTAG 2790
ACTCGGACCCGGACTACGAGACTGACGTGCAGAGTTCCGGTCATC

TGGGCAGATCAAGAGCATACACACGGCTGATTACGAACCACTCAC 2835
ACCCGTCTAGTTCTCGTATGTGTGCCGACTAATGCTTGGTGAGTG

TGAACTATTCGAGTTAGCAGTATTGATGAACCGAGGTGTTGGCCA 2880
ACTTGATAAGCTCAATCGTCATAACTACTTGGCTCCACAACCGGT

TGTCTCTTGGCAAGCTGAAAAGGATCATCGCTTGAATCCCGACGT 2925
ACAGAGAACCGTTCGACTTTTCCTAGTAGCGAACTTAGGGCTGCA

```
GGCTGTAGTTGATCAAGCACGGCTATATTCGTGTGTGCGCGACAT
                                                      2970
CCGACATCAACTAGTTCGTGCCGATATAAGCACACACGCGCTGTA

GTTCGAAGGATCAAAGCAGACGTATAAATATCCCTTTATGACGTG
                                                      3015
CAAGCTTCCTAGTTTCGTCTGCATATTTATAGGGAAATACTGCAC

GGATGACTACACTGCAAACAGATGGGAGTGGGTTCCAGGTGGCAG
                                                      3060
CCTACTGATGTGACGTTTGTCTACCCTCACCCAAGGTCCACCGTC

TGTCCACTCTCAATACGAAGAAGACAACGATTATATCTATCCTGG
                                                      3105
ACAGGTGAGAGTTATGCTTCTTCTGTTGCTAATATAGATAGGACC

TCAGTATACTAGGAACAAGTTCATAACTGTTAACAAAATGCCCAA
                                                      3150
AGTCATATGATCCTTGTTCAAGTATTGACAATTGTTTTACGGGTT

ACACAAAATATCTAGAATGATAGCATCACCGCCTGAGGTACGAGC
                                                      3195
TGTGTTTTATAGATCTTACTATCGTAGTGGCGGACTCCATGCTCG

LA-F2
TTGGACGTCGACGAAGTACG
TTGGACGTCGACGAAGTACGAATGGGGCAAGCAACGTGCTATCTA
                                                      3240
AACCTGCAGCTGCTTCATGCTTACCCCGTTCGTTGCACGATAGAT

CGGGACGGATCTACGAAGTACACTGATAACTAACTTTGCAATGTT
                                                      3285
GCCCTGCCTAGATGCTTCATGTGACTATTGATTGAAACGTTACAA

CAGGTGCGAGGATGTTCTCACTCACAAGTTCCCAGTAGGCGACCA
                                                      3330
GTCCACGCTCCTACAAGAGTGAGTGTTCAAGGGTCATCCGCTGGT

GGCAGAGGCAGCAAAGGTGCACAAACGGGTGAACATGATGCTGGA
                                                      3375
CCGTCTCCGTCGTTTCCACGTGTTTGCCCACTTGTACTACGACCT
```

```
CGGTGCCTCTAGTTTCTGCTTCGATTATGATGACTTCAATTCTCA
GCCACGGAGATCAAAGACGAAGCTAATACTACTGAAGTTAAGAGT    3420

GCATTCAATAGCTAGTATGTATACGGTTTTGTGCGCTTTCAGGGA
CGTAAGTTATCGATCATACATATGCCAAAACACGCGAAAGTCCCT    3465

CACATTTAGTCGCAACATGTCTGATGAACAAGCAGAGGCGATGAA
GTGTAAATCAGCGTTGTACAGACTACTTGTTCGTCTCCGCTACTT    3510
                                     CGCTACTT
                                     LA-R2

CTGGGTGTGTGAGTCCGTCAGACACATGTGGGTACTAGATCCTGA
GACCCACACACTCAGGCAGTCTGTGTACACCCATGATCTAGGACT    3555
GACCCACACACT
LA-R2

TACCAAGGAGTGGTACAGACTACAAGGTACATTACTGTCAGGATG
ATGGTTCCTCACCATGTCTGATGTTCCATGTAATGACAGTCCTAC    3600

GCGGTTAACCACATTTATGAACACTGTGCTAAACTGGGCGTATAT
CGCCAATTGGTGTAAATACTTGTGACACGATTTGACCCGCATATA    3645

GAAATTAGCTGGCGTATTTGATCTGGATGACGTTCAAGACTCGGT
CTTTAATCGACCGCATAAACTAGACCTACTGCAAGTTCTGAGCCA    3690

ACACAACGGTGATGATGTTATGATTAGTCTCAACCGCGTGAGCAC
TGTGTTGCCACTACTACAATACTAATCAGAGTTGGCGCACTCGTG    3735

AGCAGTAAGAATAATGGACGCTATGCACCGGATAAATGCGCGAGC
TCGTCATTCTTATTACCTGCGATACGTGGCCTATTTACGCGCTCG    3780

ACAGCCGGCGAAGTGTAACTTGTTTTCGATAAGTGAATTTCTGAG
TGTCGGCCGCTTCACATTGAACAAAAGCTATTCACTTAAAGACTC    3825
```

```
GGTAGAACACGGTATGAGCGGAGGCGATGGTCTTGGGGCTCAGTA
                                                  3870
CCATCTTGTGCCATACTCGCCTCCGCTACCAGAACCCCGAGTCAT

CTTAAGTAGGTCTTGTGCTACTCTTGTACACAGTAGGATTGAGTC
                                                  3915
GAATTCATCCAGAACACGATGAGAACATGTGTCATCCTAACTCAG

TAACGAACCACTGTCAGTAGTACGAGTTATGGAAGCAGACCAGGC
                                                  3960
ATTGCTTGGTGACAGTCATCATGCTCAATACCTTCGTCTGGTCCG

                    RW297
                    AACAGAACGCGGGTACAATC
TAGATTGCGCGACCTGGCAAACAGAACGCGGGTACAATCTGCGGT
                                                  4005
ATCTAACGCGCTGGACCGTTTGTCTTGCGCCCATGTTAGACGCCA

AACAGCGATAAAAGAACAACTCGACAAACGTGTCACTAAGATATT
                                                  4050
TTGTCGCTATTTTCTTGTTGAGCTGTTTGCACAGTGATTCTATAA

CGGAGTTGGTGATGACGTTGTGCGCGACATACACACAGCTCACAG
                                                  4095
GCCTCAACCACTACTGCAACACGCGCTGTATGTGTGTCGAGTGTC

GGTGTGTGGCGGTATCTCGACTGATACCTGGGCACCGGTTGAAAC
                                                  4140
CCACACACCGCCATAGAGCTGACTATGGACCCGTGGCCAACTTTG

                        LA-QF1                LA-F6
                        GCATATGAAATACCATACGA  T
TAAGATAATAACAGACAATGAAGCATATGAAATACCATACGAAAT
                                                  4185
ATTCTATTATTGTCTGTTACTTCGTATACTTTATGGTATGCTTTA
                                  GGTATGCTTTA
                                  LA-R5
```

```
LA-F6
AGATGATCCATCATTTTGG
AGATGATCCATCATTTTGGCCAGGGGTAAATGATTATGCTTATAA    4230
TCTACTAGGTAGTAAAACCGGTCCCCATTTACTAATACGAATATT
TCTACTAGG
LA-R5
CTACTAGGTAGTAAAACCGG
LA-R4
CTACTAGGTAGTAAAACCGG
LA-QR1

AGTCTGGAAAAATTTCGGAGAACGACTCGAATTTAATAAGATTAA    4275
TCAGACCTTTTTAAAGCCTCTTGCTGAGCTTAAATTATTCTAATT
ACCTTTTTAAAGCCTCTTGC
RW298

AGATGCCGTAGCTAGAGGGAGTAGGAGCACTATAGCTCTGAAACG    4320
TCTACGGCATCGATCTCCCTCATCCTCGTGATATCGAGACTTTGC

TAAGGCTAGGATAACATCTAAGAAGAATGAATTCGCTAACAAGTC    4365
ATTCCGATCCTATTGTAGATTCTTCTTACTTAAGCGATTGTTCAG

GGAATGGGAAAGGACAATGTACAAAGCCTATAAGGGTTTGGCAGT    4410
CCTTACCCTTTCCTGTTACATGTTTCGGATATTCCCAAACCGTCA
GTCA
LA-R6

CTCATACTATGCTAACCTGAGCAAATTCATGAGTATACCACCAAT    4455
GAGTATGATACGATTGGACTCGTTTAAGTACTCATATGGTGGTTA
GAGTATGATACGATTG
LA-R6
```

```
GGCGAACATTGAATTTGGGCAGGCTAGATATGCTATGCAAGCAGC
                                                    4500
CCGCTTGTAACTTAAACCCGTCCGATCTATACGATACGTTCGTCG

CCTTGATAGTTCTGATCCACTCCGGGCATTACAGGTCATACTGTA
                                                    4545
GGAACTATCAAGACTAGGTGAGGCCCGTAATGTCCAGTATGACAT

                                  End (4579)
ATTGCCAAAAAGATAATGGGAATTACCCATATGC    3′
                                          4579
TAACGGTTTTTCTATTACCCTTAATGGGTATACG    5′
```

***Start*** (0)

5′ GAATTTTTCGGTGAACCGGAATTATGTCGTCTCTGTTAAATTCAT 45
3′ CTTAAAAAGCCACTTGGCCTTAATACAGCAGAGACAATTTAAGTA

TACTACCAGAATATTTTAAACCTAAAACTAATTTGAATATCAACT 90
ATGATGGTCTTATAAAATTTGGATTTTGATTAAACTTATAGTTGA

CTTCTAGGGTCCAATATGGCTTTAATGCTCGCATTGATATGCAGT 135
GAAGATCCCAGGTTATACCGAAATTACGAGCGTAACTATACGTCA

ATGAAGACGATAGTGGGACTAGAAAAGGCTCAAGACCCAATGCAT 180
TACTTCTGCTATCACCCTGATCTTTTCCGAGTTCTGGGTTACGTA

TTATGTCTAACACAGTTGCTTTTATAGGAAACTATGAAGGTATTA 225
AATACAGATTGTGTCAACGAAAATATCCTTTGATACTTCCATAAT

TTGTTGATGACATTCCGATATTGGATGGTCTTAGGGCCGACATTT 270
AACAACTACTGTAAGGCTATAACCTACCAGAATCCCGGCTGTAAA

LBC-F3
TGGTGACTTAGACATGGGCC

TTGATACTCATGGTGACTTAGACATGGGCCTCGTTGAGGATGCAT 315
AACTATGAGTACCACTGAATCTGTACCCGGAGCAACTCCTACGTA

TGTCTAAGAGTACCATGATTAGAAGGAATGTACCAACTTATACTG 360
ACAGATTCTCATGGTACTAATCTTCCTTACATGGTTGAATATGAC

CTTACGCGAGTGAATTACTGTATAAGAGAAACCTTACATCTCTAT 405
GAATGCGCTCACTTAATGACATATTCTCTTTGGAATGTAGAGATA

TTTACAATATGCTCCGTTTATACTACATTAAAAAATGGGGCAGTA 450
AAATGTTATACGAGGCAAATATGATGTAATTTTTTACCCCGTCAT

TTAAGTATGAAAAAGATGCCATCTTTTATGATAATGGCCACGCCT 495
AATTCATACTTTTTCTACGGTAGAAAATACTATTACCGGTGCGGA

GTCTTTTAAACAGGCAATTGTTTCCAAAGTCTCGTGATGCTTCTT 540
CAGAAAATTTGTCCGTTAACAAAGGTTTCAGAGCACTACGAAGAA

TGGAATCAAGCCTCTCTTTGCCTGAGGCTGAAATTGCAATGCTTG 585
ACCTTAGTTCGGAGAGAAACGGACTCCGACTTTAACGTTACGAAC
CGGAGAGAAACGGACTCCGA
LBC-R3

ATCCTGGCCTGGAATTTCCAGAAGAGGATGTGCCTGCAATTTTAT 630
TAGGACCGGACCTTAAAGGTCTTCTCCTACACGGACGTTAAAATA

GGCACGGCAGAGTGTCATCCAGAGCAACGTGTATCTTAGGGCAAG 675
CCGTGCCGTCTCACAGTAGGTCTCGTTGCACATAGAATCCCGTTC

CTTGCTCAGAGTTCGCGCCTCTGGCCCCCTTTTCGATTGCGCATT 720
GAACGAGTCTCAAGCGCGGAGACCGGGGGAAAAGCTAACGCGTAA

ATTCACCACAATTGACGAGAAAACTATTTGTCAATGCGCCCGCTG 765
TAAGTGGTGTTAACTGCTCTTTTGATAAACAGTTACGCGGGCGAC

GGATTGAGCCTAGCTCCGGGCGGTATACTCACGAGGATGTAAAAG 810
CCTAACTCGGATCGAGGCCCGCCATATGAGTGCTCCTACATTTTC

ATGCGATTACGATCCTTGTGTCTGCAAACCAGGCTTATACTGACT 855
TACGCTAATGCTAGGAACACAGACGTTTGGTCCGAATATGACTGA

TTGAAGCAGCATACTTGATGCTTGCTCAAACGTTGGTCTCACCTG 900
AACTTCGTCGTATGAACTACGAACGAGTTTGCAACCAGAGTGGAC

```
TACCACGCACTGCCGAAGCAAGTGCATGGTTCATCAATGCTGGCA
                                                 945
ATGGTGCGTGACGGCTTCGTTCACGTACCAAGTAGTTACGACCGT

TGGTCAATATGCCAACTTTGTCATGTGCAAATGGTTATTATCCAG
                                                 990
ACCAGTTATACGGTTGAAACAGTACACGTTTACCAATAATAGGTC

CACTGACCAATGTCAATCCTTACCACCGGCTAGACACATGGAAAG
                                                 1035
GTGACTGGTTACAGTTAGGAATGGTGGCCGATCTGTGTACCTTTC

ATACGTTAAATCATTGGGTGGCTTATCCCGACATGCTGTTTTACC
                                                 1080
TATGCAATTTAGTAACCCACCGAATAGGGCTGTACGACAAAATGG

ATTCAGTGGCAATGATTGAGAGCTGCTATGTTGAACTCGGGAATG
                                                 1125
TAAGTCACCGTTACTAACTCTCGACGATACAACTTGAGCCCTTAC

TGGCTCGTGTGTCAGACAGTGATGCAATAAACAAATACACTTTCA
                                                 1170
ACCGAGCACACAGTCTGTCACTACGTTATTTGTTTATGTGAAAGT

CTGAGCTATCAGTGCAAGGACGGCCTGTTATGAATCGAGGAATTA
                                                 1215
GACTCGATAGTCACGTTCCTGCCGGACAATACTTAGCTCCTTAAT

TTGTAGATCTGACACTTGTGGCAATGCGTACTGGTAGGGAGATCT
                                                 1260
AACATCTAGACTGTGAACACCGTTACGCATGACCATCCCTCTAGA

CACTACCTTACCCGGTCAGCTGTGGCCTGACCCGTACAGACGCGT
                                                 1305
GTGATGGAATGGGCCAGTCGACACCGGACTGGGCATGTCTGCGCA

TATTGCAAGGTACTGAGATTCACGTTCCAGTTGTTGTCAAAGATA
                                                 1350
ATAACGTTCCATGACTCTAAGTGCAAGGTCAACAACAGTTTCTAT

TCGACATGCCCCAGTATTACAACGCGATTGATAAGGATGTTATTG
                                                 1395
AGCTGTACGGGGTCATAATGTTGCGCTAACTATTCCTACAATAAC
```

```
AGGGGCAGGAAACTGTGATTAAAGTGAAACAGCTGCCACCAGCTA
TCCCCGTCCTTTGACACTAATTTCACTTTGTCGACGGTGGTCGAT   1440

TGTATCCAATTTATACTTACGGGATCAACACTACTGAATTCTATT
ACATAGGTTAAATATGAATGCCCTAGTTGTGATGACTTAAGATAA   1485

CTGACCATTTTGAAGACCAGGTACAAGTTGAAATGGCACCAATCG
GACTGGTAAAACTTCTGGTCCATGTTCAACTTTACCGTGGTTAGC   1530

ATAATGGAAAAGCAGTTTTTAACGATGCAAGAAAGTTTTCGAAAT
TATTACCTTTTCGTCAAAAATTGCTACGTTCTTTCAAAAGCTTTA   1575

TTATGTCCATAATGCGCATGATGGGGAATGATGTTACTGCTACTG
AATACAGGTATTACGCGTACTACCCCTTACTACAATGACGATGAC   1620

ATTTAGTTACAGGTAGAAAAGTGTCGAATTGGGCCGACAACTCAT
TAAATCAATGTCCATCTTTTCACAGCTTAACCCGGCTGTTGAGTA   1665

CAGGGCGTTTCTTGTACACGGATGTGAAGTATGAAGGACAAACTG
GTCCCGCAAAGAACATGTGCCTACACTTCATACTTCCTGTTTGAC   1710

CCTTTTTGGTTGATATGGATACTGTCAAGGCGAGAGACCACTGTT
GGAAAAACCAACTATACCTATGACAGTTCCGCTCTCTGGTGACAA   1755

GGGTGTCAATTGTTGATCCTAATGGTACAATGAACTTGTCATATA
CCCACAGTTAACAACTAGGATTACCATGTTACTTGAACAGTATAT   1800

AGATGACCAATTTTAGAGCAGCAATGTTTTCTAGAAACAAGCCCT
TCTACTGGTTAAAATCTCGTCGTTACAAAAGATCTTTGTTCGGGA   1845

TGTATATGACAGGGGGGTCAGTCAGGACCATAGCTACTGGCAATT
ACATATACTGTCCCCCCAGTCAGTCCTGGTATCGATGACCGTTAA   1890
```

ATCGAGATGCTGCTGAAAGATTACGTGCAATGGATGAAACGCTCA 1935
TAGCTCTACGACGACTTTCTAATGCACGTTACCTACTTTGCGAGT

GATTAAAACCTTTTAAGATTACTGAGAAGTTGGATTTTCGTGTAG 1980
CTAATTTTGGAAAATTCTAATGACTCTTCAACCTAAAAGCACATC

CAGCTTACGCGATACCAAGTTTGTCGGGCAGCAATATGCCATCCT 2025
GTCGAATGCGCTATGGTTCAAACAGCCCGTCGTTATACGGTAGGA

TACACCATCAGGAACAACTACAGATATCAGAAGTGGACGCGGAAC 2070
ATGTGGTAGTCCTTGTTGATGTCTATAGTCTTCACCTGCGCCTTG

CAATCAATCCTATAGGAGAGGACGAACTTCCACCGGATATAGAAT 2115
GTTAGTTAGGATATCCTCTCCTGCTTGAAGGTGGCCTATATCTTA

AGGTGTCGAAGACGATGAGGACTTAGATATTGGTACGGTCAAATA 2160
TCCACAGCTTCTGCTACTCCTGAATCTATAACCATGCCAGTTTAT

CATTGTGCCATTGTATTTGAACGGTGATAATGTGGCACAAAATTG 2205
GTAACACGGTAACATAAACTTGCCACTATTACACCGTGTTTTAAC

TTTAGAAGCAACACACGTGCTTATCAAAGCTTGTAGTATTGCGAA 2250
AAATCTTCGTTGTGTGCACGAATAGTTTCGAACATCATAACGCTT

CCGGATTGTAGATGACGGAGAGGGTCACTGTTTCACACAGCAAGG 2295
GGCCTAACATCTACTGCCTCTCCCAGTGACAAAGTGTGTCGTTCC

GCTGGCGCAGCAGTGGATCTTCCATAGGGGGGAGATGATATTTGT 2340
CGACCGCGTCGTCACCTAGAAGGTATCCCCCCTCTACTATAAACA

GAAGGCGGTACGCATTGGTCAACTCAATGCATATTATGTAGACTA 2385
CTTCCGCCATGCGTAACCAGTTGAGTTACGTATAATACATCTGAT

```
TAAGAACGTCACAAATTATAGTCTTAAAACCGCTGCTCAAGTAGG  2430
ATTCTTGCAGTGTTTAATATCAGAATTTTGGCGACGAGTTCATCC

AGCGACGATATCAAATAACTTACGCCACGGATTTGTTGACAATCA  2475
TCGCTGCTATAGTTTATTGAATGCGGTGCCTAAACAACTGTTAGT

ACAAGACGCATACACGCGCTTGGTTGCCAACTACTCTGATACGCG  2520
TGTTCTGCGTATGTGCGCGAACCAACGGTTGATGAGACTATGCGC

GAAGTGGATACGTGACAATTTTACATATAATTATAATATGGAGAA  2565
CTTCACCTATGCACTGTTAAAATGTATATTAATATTATACCTCTT

AGAAAAGTATAGGATAACCCAATACCACCATACACATGTGAGGTT  2610
TCTTTTCATATCCTATTGGGTTATGGTGGTATGTGTACACTCCAA

GAAAGATTTGTTTCCATCCAGGAAAATAGTTAAACTAGAGGGATA  2655
CTTTCTAAACAAAGGTAGGTCCTTTTATCAATTTGATCTCCCTAT

TGAAGCCTTGTTGGCAATGATGCTAGACAGGTTTAACAACATAGA  2700
ACTTCGGAACAACCGTTACTACGATCTGTCCAAATTGTTGTATCT

GTCAACACATGTAACTTTCTTCACATATTTAAGAGCACTACCTGA  2745
CAGTTGTGTACATTGAAAGAAGTGTATAAATTCTCGTGATGGACT

CCGTGAAAAAGAAGTCTTTATTAGCTTAGTCTTAAACTATAATGG  2790
GGCACTTTTTCTTCAGAAATAATCGAATCAGAATTTGATATTACC

CCTTGGCAGAGAGTGGTTGAAGTCTGAAGGTGTTAGGGCTAAACA  2835
GGAACCGTCTCTCACCAACTTCAGACTTCCACAATCCCGATTTGT

AGCACAAGGTACTGTGAAATACGATATGAGTAAACTATTTGAACT  2880
TCGTGTTCCATGACACTTTATGCTATACTCATTTGATAAACTTGA
```

```
GAATGTACTAGAGAACGGAGTTGACGAAGAAGTTGACTGGGAGAA
                                                    2925
CTTACATGATCTCTTGCCTCAACTGCTTCTTCAACTGACCCTCTT

AGAGAAACGCAACAGGTCAGATATCAAGACTGTTAACATAAGTTA
                                                    2970
TCTCTTTGCGTTGTCCAGTCTATAGTTCTGACAATTGTATTCAAT

TGCAAAAGTTCTCGAACATTGTAGAGAGCTATTCATCATGGCGAG
                                                    3015
ACGTTTTCAAGAGCTTGTAACATCTCTCGATAAGTAGTACCGCTC

GGCCGAAGGGAAACGGCCAATGAGGATGAAATGGCAAGAGTACTG
                                                    3060
CCGGCTTCCCTTTGCCGGTTACTCCTACTTTACCGTTCTCATGAC

GAGGCAGAGAGCAGTTATCATGCCAGGTGGATCGGTCCACAGTCA
                                                    3105
CTCCGTCTCTCGTCAATAGTACGGTCCACCTAGCCAGGTGTCAGT

ACATCCAGTCGAACAGGACGTGATTAGAGTATTACCCAGAGAAAT
                                                    3150
TGTAGGTCAGCTTGTCCTGCACTAATCTCATAATGGGTCTCTTTA

CAGAAGTAAGAAGGGGGTGGCAAGTGTCATGCCATACAAAGAACA
                                                    3195
GTCTTCATTCTTCCCCCACCGTTCACAGTACGGTATGTTTCTTGT

GAAGTATTTCACGTCCAGAAGGCCGGAAATACACGCTTACACTTC
                                                    3240
CTTCATAAAGTGCAGGTCTTCCGGCCTTTATGTGCGAATGTGAAG

AACGAAATACGAGTGGGGAAAAGTGAGGGCACTATATGGGTGTGA
                                                    3285
TTGCTTTATGCTCACCCCTTTTCACTCCCGTGATATACCCACACT

TTTTTCATCACATACAATGGCTGATTTTGGATTGTTACAATGCGA
                                                    3330
AAAAAGTAGTGTATGTTACCGACTAAAACCTAACAATGTTACGCT

GGATACATTCCCGGGCTTTGTACCAACAGGGTCTTACGCCAATGA
                                                    3375
CCTATGTAAGGGCCCGAAACATGGTTGTCCCAGAATGCGGTTACT
```

GGATTATGTCAGGACCAGAATTGCTGGGACTCACTCATTGATCCC 3420
CCTAATACAGTCCTGGTCTTAACGACCCTGAGTGAGTAACTAGGG

TTTCTGTTACGATTTCGATGATTTCAACAGCCAACATTCAAAGGA 3465
AAAGACAATGCTAAAGCTACTAAAGTTGTCGGTTGTAAGTTTCCT

AGCCATGCAAGCAGTGATTGATGCATGGATATCTGTCTATCACGA 3510
TCGGTACGTTCGTCACTAACTACGTACCTATAGACAGATAGTGCT

TAAGTTAACAGATGACCAGATAGAGGCGGCAAAGTGGACACGAAA 3555
ATTCAATTGTCTACTGGTCTATCTCCGCCGTTTCACCTGTGCTTT

CTCGGTAGATAGAATGGTCGCTCACCAACCTAACACTGGTGAGAC 3600
GAGCCATCTATCTTACCAGCGAGTGGTTGGATTGTGACCACTCTG

LBC-QF2
AGTGGCTGGCGATTAACAAC

LBC-F2
ACACTGTTTAGTGGCTGGCG

TTATGATGTTAAAGGGACACTGTTTAGTGGCTGGCGATTAACAAC 3645
AATACTACAATTTCCCTGTGACAAATCACCGACCGCTAATTGTTG

ATTTTTCAATACGGCGTTGAACTATTGCTACCTGGCTAATGCAGG 3690
TAAAAAGTTATGCCGCAACTTGATAACGATGGACCGATTACGTCC

TATAAACTCACTAGTGCCAACGAGTCTCCATAATGGTGATGATGT 3735
ATATTTGAGTGATCACGGTTGCTCAGAGGTATTACCACTACTACA

ACTACTACA
LBC-QR2

TTTTGCAGGGATAAGGACAATAGCTGACGGTATTTCTTTGATCAA 3780
AAAACGTCCCTATTCCTGTTATCGACTGCCATAAAGAAACTAGTT

AAAACGTCCCT
LBC-QR2

AAACGCCGCAGCCACGGGAGTTCGCGCTAATACAACTAAAATGAA 3825
TTTGCGGCGTCGGTGCCCTCAAGCGCGATTATGTTGATTTTACTT

CATTGGTACGATAGCAGAGTTTTTGAGAGTTGATATGCGTGCAAA 3870
GTAACCATGCTATCGTCTCAAAAACTCTCAACTATACGCACGTTT

AAATAGTACTGGCAGTCAGTATTTAACAAGAGGGATTGCTACCTT 3915
TTTATCATGACCGTCAGTCATAAATTGTTCTCCCTAACGATGGAA

CCCTAACGATGGAA
LBC-R2

CACGCACAGTAGGGTTGAGTCTGATGCACCACTGACATTGCGCAA 3960
GTGCGTGTCATCCCAACTCAGACTACGTGGTGACTGTAACGCGTT

GTGCGT
LBC-R2

TCTAGTATCTGCTTACAAAACCAGATATGACGAGATTTTAGCTCG 4005
AGATCATAGACGAATGTTTTGGTCTATACTGCTCTAAAATCGAGC

TGGCGCAAGCATCGATAACATGAAGCCACTCTATCGTAAGCAATT 4050
ACCGCGTTCGTAGCTATTGTACTTCGGTGAGATAGCATTCGTTAA

ATTTTTTGCTAGAAAGTTGTTCAATGTCGAGAAGGACATTGTTGA 4095
TAAAAAACGATCTTTCAACAAGTTACAGCTCTTCCTGTAACAACT

CAATCTGATAACGATGGACATATCATGTGGCGGTTTGCAAGAAAA 4140
GTTAGACTATTGCTACCTGTATAGTACACCGCCAAACGTTCTTTT

```
GGGTAGGGTATCAGAGATGGTGTTACAGGAGGTTGACATTGAGAA
                                                        4185
CCCATCCCATAGTCTCTACCACAATGTCCTCCAACTGTAACTCTT

TATAGATAGTTATAGGAAGACAAGGATGATCGCCAAACTGATTGA
                                                        4230
ATATCTATCAATATCCTTCTGTTCCTACTAGCGGTTTGACTAACT

        RW301
 AAGGGGGTTGGCGATTATAC
CAAGGGGGTTGGCGATTATACTGCATTCCTGAAAACTAACTTTTC
                                                        4275
GTTCCCCCAACCGCTAATATGACGTAAGGACTTTTGATTGAAAAG

CGAGATAGCTGATGCTATCACAAGAGAGACACGTGTAGAGTCAGT
                                                        4320
GCTCTATCGACTACGATAGTGTTCTCTCTGTGCACATCTCAGTCA

GACCAAGGCTTATAATGTTAAGAAGAAAACGGTCGTACGCGCGTT
                                                        4365
CTGGTTCCGAATATTACAATTCTTCTTTTGCCAGCATGCGCGCAA

TAGGGACCTAAGCGCAGCATATCATGAAAGAGCGGTGAGACATGC
                                                        4410
ATCCCTGGATTCGCGTCGTATAGTACTTTCTCGCCACTCTGTACG
                           TTCTCGCCACTCTGTACG
                                   RW302

TTGGAAGGGGATGAGTGGACTACACATAGTCAACAGGATTCGTAT
                                                        4455
AACCTTCCCCTACTCACCTGATGTGTATCAGTTGTCCTAAGCATA
AA
RW302

GGGAGTGAGCAACTTAGTAATGGTTGTTAGCAAAATCAATCCTGC
                                                        4500
CCCTCACTCGTTGAATCATTACCAACAATCGTTTTAGTTAGGACG

AAAAGCTAATGTGCTAGCCAAATCAGGAGATCCTACAAAATGGCT
                                                        4545
TTTTCGATTACACGATCGGTTTAGTCCTCTAGGATGTTTTACCGA
```

```
TGCAGTCCTTACATGATATACAGGCAACCACATAAGACCTGAGAA
                                                  4590
ACGTCAGGAATGTACTATATGTCCGTTGGTGTATTCTGGACTCTT

                         End (4615)
CAAAGAGTACATACGATACTACGCA    3′
                               4615
GTTTCTCATGTATGCTATGATGCGT    5′
```

Sequence: S.cerevisiae 20S narnavirus.dna (Linear / 2514 bp)
Enzymes: Unique 6+ Cutters (24 of 678 total)
Primers: 4 total

Unique Cutters **Bold**

***Start*** (0)

```
5′  GGGGCTGATCCCATGAAGGAACCAGTAGACTGCCGTCTTTCGACG
                                                   45
3′  CCCCGACTAGGGTACTTCCTTGGTCATCTGACGGCAGAAAGCTGC

    CCAGCCGGTTTCTCGGGGACAGTCCCCCCTCCTGGTCGCACTAAG
                                                   90
    GGTCGGCCAAAGAGCCCCTGTCAGGGGGGAGGACCAGCGTGATTC

    GCGGCCAGGCCGGGAACCATCCCTGTGAGGCGTTCGCGTGGAAGC
                                                   135
    CGCCGGTCCGGCCCTTGGTAGGGACACTCCGCAAGCGCACCTTCG

    GCGTCTGCCTTACCGGGTAAAATCTACGGTTGGAGCCGTCGACAA
                                                   180
    CGCAGACGGAATGGCCCATTTTAGATGCCAACCTCGGCAGCTGTT

    CGGGATAGGTTCGCGATGTTGCTGTCGTCTTTCGACGCGGCTCTC
                                                   225
    GCCCTATCCAAGCGCTACAACGACAGCAGAAAGCTGCGCCGAGAG

    GCGGCCTACTCCGGCGTCGTCGTCTCCAGAGGTACACGCTCTCTA
                                                   270
    CGCCGGATGAGGCCGCAGCAGCAGAGGTCTCCATGTGCGAGAGAT

    CCGCCATCGCTCCGGTTATTCCGGGCGATGACGCGTAAGTGGCTT
                                                   315
    GGCGGTAGCGAGGCCAATAAGGCCCGCTACTGCGCATTCACCGAA

    TCAGTGACCGCCCGCGGGAACGGGGTCGAGTTCGCCATCGCTTCT
                                                   360
    AGTCACTGGCGGGCGCCCTTGCCCCAGCTCAAGCGGTAGCGAAGA

    GCGAAGGAGTTCTCAGCCGCGTGCCGCGCGGGTTGGATTTCGGGG
                                                   405
    CGCTTCCTCAAGAGTCGGCGCACGGCGCGCCCAACCTAAAGCCCC

    ACCGTTCCGGACCACTTCTTTATGAAGTGGCTTCCGGAACCCGTT
                                                   450
    TGGCAAGGCCTGGTGAAGAAATACTTCACCGAAGGCCTTGGGCAA

    CGCCGTAAATCCGGGTTGTGGGCCCAGCTTTCATTCATTGGACGT
                                                   495
    GCGGCATTTAGGCCCAACACCCGGGTCGAAAGTAAGTAACCTGCA
```

```
TCGCTCCCCGAGGGGGGCGACCGCCATGAAATCGAGGCGTTGGCC
                                                 540
AGCGAGGGGCTCCCCCCGCTGGCGGTACTTTAGCTCCGCAACCGG

AACCACAAGGCTGCGTTGTCCAGTTCCTTTGAGGTTCCTGCGGAC
                                                 585
TTGGTGTTCCGACGCAACAGGTCAAGGAAACTCCAAGGACGCCTG

GTACTTACGTCTCTAAGGAATTACTCAGAGGACTGGGCCCGCCGC
                                                 630
CATGAATGCAGAGATTCCTTAATGAGTCTCCTGACCCGGGCGGCG

CACCTCGCTGCGGATCCTGATCCTTCGCTGCTCTGTGAGCCCTGT
                                                 675
GTGGAGCGACGCCTAGGACTAGGAAGCGACGAGACACTCGGGACA

ACGGGTAACAGCGCAACGTTCGAACGGACTCGCCGCGAGGGTGGT
                                                 720
TGCCCATTGTCGCGTTGCAAGCTTGCCTGAGCGGCGCTCCCACCA

TTTGCACAATCGATCACTGACTTGGTTTCGTCCTCACCCACTGAC
                                                 765
AAACGTGTTAGCTAGTGACTGAACCAAAGCAGGAGTGGGTGACTG

AACCTCCCTCCCCTTGAGTCGATGCCCTTCGGGCCGACCCAAGGC
                                                 810
TTGGAGGGAGGGGAACTCAGCTACGGGAAGCCCGGCTGGGTTCCG

CAGGCGTTGCCAGTGCACGTGCTCGAGGTCTCTCTCTCTCGATAC
                                                 855
GTCCGCAACGGTCACGTGCACGAGCTCCAGAGAGAGAGAGCTATG

CACAATGGCTCAGACCCTAAGGGTAGAGTCTCTGTGGTGAGGGAG
                                                 900
GTGTTACCGAGTCTGGGATTCCCATCTCAGAGACACCACTCCCTC

AGAGGCCACAAGGTCCGTGTGGTCTCTGCAATGGAGACTCACGAA
                                                 945
TCTCCGGTGTTCCAGGCACACCAGAGACGTTACCTCTGAGTGCTT

CTTGTACTCGGTCACGCGGCTAGGCGCAGACTCTTTAAGGGACTG
                                                 990
GAACATGAGCCAGTGCGCCGATCCGCGTCTGAGAAATTCCCTGAC
```

CGTCGTGAGCGTCGTCTCAGGGACACCCTCAAGGGTGACTTCGAG
1035
GCAGCACTCGCAGCAGAGTCCCTGTGGGAGTTCCCACTGAAGCTC

GCGACAACCAAGGCCTTTGTGGGTTGTGCTGGTACCGTTATCTCA
1080
CGCTGTTGGTTCCGGAAACACCCAACACGACCATGGCAATAGAGT

20S-F2
tctgcctcggacctcatccc
TCAGATATGAAATCTGCCTCGGACCTCATCCCTCTATCGGTCGCT
1125
AGTCTATACTTTAGACGGAGCCTGGAGTAGGGAGATAGCCAGCGA

TCTGCGATCGTAGATGGTCTGGAGGCCTCTGGTAGACTCCTACCT
1170
AGACGCTAGCATCTACCAGACCTCCGGAGACCATCTGAGGATGGA

GTCGAGATAGCTGGTCTTCGGGCCTGTACTGGCCCTCAGCACTTA
1215
CAGCTCTATCGACCAGAAGCCCGGACATGACCGGGAGTCGTGAAT

RW307
TGACGGTTCTGAAATCACCA
GTCTACCCTGACGGTTCTGAAATCACCACACGGCGAGGGATCCTT
1260
CAGATGGGACTGCCAAGACTTTAGTGGTGTGCCGCTCCCTAGGAA

ATGGGACTCCCCACCACGTGGGCGATTCTGAATCTCATGCACCTA
1305
TACCCTGAGGGGTGGTGCACCCGCTAAGACTTAGAGTACGTGGAT

TGGTGCTGGGATTCTGCGGATCGTCAGTATAGATTAGAGGGACAT
1350
ACCACGACCCTAAGACGCCTAGCAGTCATATCTAATCTCCCTGTA

CCCTTCCGCGCCACGGTTAGATCGGATTGTCGCGTTTGCGGCGAC
1395
GGGAAGGCGCGGTGCCAATCTAGCCTAACAGCGCAAACGCCGCTG
cgcggtgccaatctagccta
20S-R2

```
GATCTAATCGGCGTGGGTCCGGACTCCTTACTACGGTCTTATGAC
                                                1440
CTAGATTAGCCGCACCCAGGCCTGAGGAATGATGCCAGAATACTG

CGCAATTTGGGTCTGGTTGGGATGATCCTCTCCCCTGGCAAGCAC
                                                1485
GCGTTAAACCCAGACCAACCCTACTAGGAGAGGGGACCGTTCGTG
                                      GTTCGTG
                                        RW308

TTCCGCAGTAACAGGAGGGGGGTCTTCCTCGAGCGTTTACTCGAG
                                                1530
AAGGCGTCATTGTCCTCCCCCCAGAAGGAGCTCGCAAATGAGCTC
AAGGCGTCATTGT
    RW308

TTCCAGACCCGTAAAACCGTGTACGAACACGCTGTGATTTACCGT
                                                1575
AAGGTCTGGGCATTTTGGCACATGCTTGTGCGACACTAAATGGCA

AAGGTAGGTCACCGTCGCGTGCCCGTGGATCGGTCTCACATTCCC
                                                1620
TTCCATCCAGTGGCAGCGCACGGGCACCTAGCCAGAGTGTAAGGG

GTCGTCACCCGAGTGACCGTCCTGAATACCATCCCACTTAAAGGG
                                                1665
CAGCAGTGGGCTCACTGGCAGGACTTATGGTAGGGTGAATTTCCC

TTGGTTCGGGCTTCGGTTCTCGGTCGTGACGATCCTCCCGTTTGG
                                                1710
AACCAAGCCCGAAGCCAAGAGCCAGCACTGCTAGGAGGGCAAACC

TGGGCTGCGGCCGTGGCGGAGTCTTCACTGCTCAGTGACTATCCT
                                                1755
ACCCGACGCCGGCACCGCCTCAGAAGTGACGAGTCACTGATAGGA

CGTAAGAAGATATTCGCTGCAGCACGGACTCTCCGCCCTGGCCTC
                                                1800
GCATTCTTCTATAAGCGACGTCGTGCCTGAGAGGCGGGACCGGAG

TCCCGCCAATTCAGAAGGTTGGGAATCCCACCATTCCTCCCCCGT
                                                1845
AGGGCGGTTAAGTCTTCCAACCCTTAGGGTGGTAAGGAGGGGGCA
```

```
GAACTCGGGGGCGCAGGCTTGGTCGGACCTTCCGATCGTGTCGAC
                                                 1890
CTTGAGCCCCCGCGTCCGAACCAGCCTGGAAGGCTAGCACAGCTG

GCCCCTGCGTTCCACAGGAAAGCCATTTCTTCCCTGGTGTGGGGC
                                                 1935
CGGGGACGCAAGGTGTCCTTTCGGTAAAGAAGGGACCACACCCCG

TCTGATGCCACTGCTGCATACAGTTTTATCCGTATGTGGCAGGGG
                                                 1980
AGACTACGGTGACGACGTATGTCAAAATAGGCATACACCGTCCCC

TTCGAGGGCCACCCTTGGAAGACGGCGGCCTCACAGGAGACGGAC
                                                 2025
AAGCTCCCGGTGGGAACCTTCTGCCGCCGGAGTGTCCTCTGCCTG

ACTTGGTTCGCCGACTATAAGGTCACCCGGCCGGGTAAGATGTAC
                                                 2070
TGAACCAAGCGGCTGATATTCCAGTGGGCCGGCCCATTCTACATG

CCAGACCGTTACGGCTTTCTTGATGGAGAGTCTCTTCGGACCAAG
                                                 2115
GGTCTGGCAATGCCGAAAGAACTACCTCTCAGAGAAGCCTGGTTC

TCAACTATGTTGAACTCGGCCGTCTATGAGACTTTTCTCGGACCT
                                                 2160
AGTTGATACAACTTGAGCCGGCAGATACTCTGAAAAGAGCCTGGA

GACCCTGACGCCACCCATTACCCTTCCTTGCGAATCGTCGCCAGT
                                                 2205
CTGGGACTGCGGTGGGTAATGGGAAGGAACGCTTAGCAGCGGTCA

AGACTGGCGAAGGTCCGGAAGGATTTGGTAAATCGGTGGCCATCG
                                                 2250
TCTGACCGCTTCCAGGCCTTCCTAAACCATTTAGCCACCGGTAGC

GTCAAGCCCGTGGGGAAGGATCTTGGTACCATCTTAGAAGCTTTC
                                                 2295
CAGTTCGGGCACCCCTTCCTAGAACCATGGTAGAATCTTCGAAAG

GAAGAGTCAAAGTTGTGCACCCTTTGGGTGACACCTTACGACGCT
                                                 2340
CTTCTCAGTTTCAACACGTGGGAAACCCACTGTGGAATGCTGCGA
```

```
TCGGGCTACTTTGATGATTCCTTGTTACTGATGGATGAGAGCGTG
                                                2385
AGCCCGATGAAACTACTAAGGAACAATGACTACCTACTCTCGCAC

TACCAACGTAGATTCCGGCAACTGGTCATTGCCGGCTTGATGCGT
                                                2430
ATGGTTGCATCTAAGGCCGTTGACCAGTAACGGCCGAACTACGCA

GAGGGCCGGATGGGCGACTTATTGTTTCCCAACTGGCTTCCACCA
                                                2475
CTCCCGGCCTACCCGCTGAATAACAAAGGGTTGACCGAAGGTGGT

                                         End (2514)
TCCACCGTGGTCTCGGGTTTCCCCTGAGGCCACGGCCCC     3'
                                              2514
AGGTGGCACCAGAGCCCAAAGGGGACTCCGGTGCCGGGG     5'
```

Sequence: Saccharomyces 23S RNA narnavirus, complete genome .dna (Linear / 2891 bp)
Enzymes: Unique 6+ Cutters (43 of 678 total) Unique Cutters **Bold**
Primers: 4 total

***Start*** (0)

5′ GGGGCCATGCATCACAAGGTTAATGTGAAAACACAGCGAGAAGTC
45
3′ CCCCGGTACGTAGTGTTCCAATTACACTTTTGTGTCGCTCTTCAG

CATTTCCCAATGGACCTCTTGCAAGCCTGTGGTGCATCTGCCCCT
90
GTAAAGGGTTACCTGGAGAACGTTCGGACACCACGTAGACGGGGA

AGGCCAGTTGCCCGTGTTTCACGTGCAACCGACCTAGACCGACGC
135
TCCGGTCAACGGGCACAAAGTGCACGTTGGCTGGATCTGGCTGCG

**BtsI**

TACAGGTGCGTCCTCAGTTTACCTGAGGAGCGTGCTCGCAGTGTT
180
ATGTCCACGCAGGAGTCAAATGGACTCCTCGCACGAGCGTCACAA

GGGTGTAAATGGTCGTCGACCCGAGCGGCGTTACGACGTGGACTC
225
CCCACATTTACCAGCAGCTGGGCTCGCCGCAATGCTGCACCTGAG

GAGGAGCTTGGCTCCCGCGAGTTCCGCCGTCGTCTCCGTTTGGCG
270
CTCCTCGAACCGAGGGCGCTCAAGGCGGCAGCAGAGGCAAACCGC

GACGATTGCTGGCGCGCGATCTGCGCGGCCGTCTGCACGGGTCGG
315
CTGCTAACGACCGCGCGCTAGACGCGCCGGCAGACGTGCCCAGCC

AAGTTTCCTTCCTTCTCGGTGACAGATCGGCCGGCAAGAGCTCGC
360
TTCAAAGGAAGGAAGAGCCACTGTCTAGCCGGCCGTTCTCGAGCG

CTTGCAAAAGTCTACCGTATGGGTCGTCGACTGCTAGTAGGTGTG
405
GAACGTTTTCAGATGGCATACCCAGCAGCTGACGATCATCCACAC

GTCTGCCGAGGCGAATCGGTCGTCTCCGACCTCAAGCAGGAGTGT
450
CAGACGGCTCCGCTTAGCCAGCAGAGGCTGGAGTTCGTCCTCACA

GCAGATTTGCGGCGTGTGATCTTCGAAGGGTCCACCCGGATCCCG 495
CGTCTAAACGCCGCACACTAGAAGCTTCCCAGGTGGGCCTAGGGC

TCCAGTTCCTTATGGGGACTGGTCGGGGTCCTAGGTTGGACCTCT 540
AGGTCAAGGAATACCCCTGACCAGCCCCAGGATCCAACCTGGAGA

CCGGAGAGGGCGATGCAGCTGACGTTCATCGGCCGGGCTCTCCCT 585
GGCCTCTCCCGCTACGTCGACTGCAAGTAGCCGGCCCGAGAGGGA

TATGGGAGTCCAGACGTTGAGCGTCGTGCTCTCGCCTCACATGCC 630
ATACCCTCAGGTCTGCAACTCGCAGCACGAGAGCGGAGTGTACGG

GCCACTCTGTCCATCCCCGCTGAGTGCCATCCTAACTACCTCGTA 675
CGGTGAGACAGGTAGGGGCGACTCACGGTAGGATTGATGGAGCAT

GCCGCGGAACAATTCGCGAAATCCTGGGCAGATGATAACCTGCCC 720
CGGCGCCTTGTTAAGCGCTTTAGGACCCGTCTACTATTGGACGGG

CGGAAATTCCGGATTTATCCCATTGCGGTGCAGGAGTCCTCCTGT 765
GCCTTTAAGGCCTAAATAGGGTAACGCCACGTCCTCAGGAGGACA

ATGGAGTACTCCCGTGCGCAAGGCGGTCTACTGCAGTCGTTTAGG 810
TACCTCATGAGGGCACGCGTTCCGCCAGATGACGTCAGCAAATCC

AAAGGCTTTGTCGGGTACGATCCCGCGGCGCCTTCGGCAGACCCT 855
TTTCCGAAACAGCCCATGCTAGGGCGCCGCGGAAGCCGTCTGGGA

GATGATCTCGAGTTGGCAAAGGAGCGTGGATTCTCTCGAATCCGC 900
CTACTAGAGCTCAACCGTTTCCTCGCACCTAAGAGAGCTTAGGCG

GCCTCTTGGTACTCCACTTTCCGCTATCGCGGAGAGTTGAAGTCC 945
CGGAGAACCATGAGGTGAAAGGCGATAGCGCCTCTCAACTTCAGG

```
ACCAATCAGTCTCTCGAGGCCCGGGTCGCTGTGGTCCCTGAGAGG
                                                990
TGGTTAGTCAGAGAGCTCCGGGCCCAGCGACACCAGGGACTCTCC

GGGTTCAAGGCGAGGATTGTGACCACGCACAGCGCATCGCGCGTG
                                                1035
CCCAAGTTCCGCTCCTAACACTGGTGCGTGTCGCGTAGCGCGCAC

ACGTTCGGTCATCAATTCCGCCGTTACCTCCTCCAGGGGATCAGG
                                                1080
TGCAAGCCAGTAGTTAAGGCGGCAATGGAGGAGGTCCCCTAGTCC

                        RW313
                        ATTGGAGGCGATCATAGACG
CGACACCCGGCTCTGGTTGACGTGATTGGAGGCGATCATAGACGA
                                                1125
GCTGTGGGCCGAGACCAACTGCACTAACCTCCGCTAGTATCTGCT

GCTGTCGAAACTATGGATGGCGACTTCGGCCTGTTAAGGCCAGAT
                                                1170
CGACAGCTTTGATACCTACCGCTGAAGCCGGACAATTCCGGTCTA

GGTCGTCTCCTTTCCGCGGACTTGACGTCAGCATCTGACCGGATA
                                                1215
CCAGCAGAGGAAAGGCGCCTGAACTGCAGTCGTAGACTGGCCTAT

          23S-F2
          TGGTGAAAGCCATTTTGCGGGGC
CCCCATGACTTGGTGAAAGCCATTTTGCGGGGCATCTTCTCGGAT
                                                1260
GGGGTACTGAACCACTTTCGGTAAAACGCCCCGTAGAAGAGCCTA

CCTGATCGACGTCCTCCGGGAACGTCCCTTGCGGACGTTTTCGAC
                                                1305
GGACTAGCTGCAGGAGGCCCTTGCAGGGAACGCCTGCAAAAGCTG

CTCGTCCTCGGACCCTATCACCTTCACTACCCTGACGGTAGTGAA
                                                1350
GAGCAGGAGCCTGGGATAGTGGAAGTGATGGGACTGCCATCACTT
```

```
GTGACCGTTCGTCAGGGAATCCTTATGGGACTCCCGACGACCTGG    1395
CACTGGCAAGCAGTCCCTTAGGAATACCCTGAGGGCTGCTGGACC
     GCAAGCAGTCCCTTAGGAAT
     RW314

CCTCTGCTATGTCTAATCCACCTGTTCTGGGTGGAACTCTCCGAT    1440
GGAGACGATACAGATTAGGTGGACAAGACCCACCTTGAGAGGCTA

TGGGCCCCCGCGCGTCCTAATCATTCGCGCGGGTTTGTCCTCGGG    1485
ACCCGGGGGCGCGCAGGATTAGTAAGCGCGCCCAAACAGGAGCCC

GAGTCCTTCAGAATTTGTGGCGACGACCTGATTGCTTGGTGGCGA    1530
CTCAGGAAGTCTTAAACACCGCTGCTGGACTAACGAACCACCGCT
                                 CGAACCACCGCT
                                 23S-R2

CCCGAAAGGATCGCCCTCTACAATCAGATTGCCGTAGACTGCGGA    1575
GGGCTTTCCTAGCGGGAGATGTTAGTCTAACGGCATCTGACGCCT
GGGCTTTCCTA
23S-R2

GCCCAGTTTTCAGCGGGGAAGCACCTCGAGTCGAAGACTTGGGGG    1620
CGGGTCAAAAGTCGCCCCTTCGTGGAGCTCAGCTTCTGAACCCCC

ATCTTCACTGAAAAGGTCTTCACGGTTAAACCCGTGAAGATGAAG    1665
TAGAAGTGACTTTTCCAGAAGTGCCAATTTGGGCACTTCTACTTC

GTCCGGGTTCGCTCTGAGCCATCTTTGAAGGGCTACGTGTTCTCC    1710
CAGGCCCAAGCGAGACTCGGTAGAAACTTCCCGATGCACAAGAGG

CGATCTAGTGCCTTCTCCTGCCGAATGGGAGGAAAGGGCATAACT    1755
GCTAGATCACGGAAGAGGACGGCTTACCCTCCTTTCCCGTATTGA
```

```
GGTATCCGGGCGGCCCGGCTATACACCATCGGAGCGATGCCTCGT
                                                1800
CCATAGGCCCGCCGGGCCGATATGTGGTAGCCTCGCTACGGAGCA

TGGAGTCGTCGCATCCGGGATGTCTATCCTGGTTCGCTCGAACAT
                                                1845
ACCTCAGCAGCGTAGGCCCTACAGATAGGACCAAGCGAGCTTGTA

CGTACTGCTTCTCAGCGGTACGGTGAACCGGTTACTGTGTACCGT
                                                1890
GCATGACGAAGAGTCGCCATGCCACTTGGCCAATGACACATGGCA

TTTGGTCGCTGGTCTTCCGCAATTCCACTTCGGTGGGCTGTTCGG
                                                1935
AAACCAGCGACCAGAAGGCGTTAAGGTGAAGCCACCCGACAAGCC

GCCCCCACCCGTACGGTGGGGAACCCTGTCCAGTCCTTACCGGAT
                                                1980
CGGGGGTGGGCATGCCACCCCTTGGGACAGGTCAGGAATGGCCTA

TGGTTCACGGTGGGACCAGCTGCTTCTTCGGTCGCTGCTGATTCA
                                                2025
ACCAAGTGCCACCCTGGTCGACGAAGAAGCCAGCGACGACTAAGT

AACGCCTTTGGGGCGGTATCGAGGGTCCTTCGACGGATGTTCCCG
                                                2070
TTGCGGAAACCCCGCCATAGCTCCCAGGAAGCTGCCTACAAGGGC

GGTTTACCCCGGAAACTTGCGTCAGCGGGCATTCCTCCGTACCTC
                                                2115
CCAAATGGGGCCTTTGAACGCAGTCGCCCGTAAGGAGGCATGGAG

CCTCGGGTGTTTGGCGGCGGCGGTCTGGTGAAATCCACCGGACTC
                                                2160
GGAGCCCACAAACCGCCGCCGCCAGACCACTTTAGGTGGCCTGAG

ACAACTAAGATCGGGGCCGTTGCTTCCCGCCGCTGGATGAGTCGG
                                                2205
TGTTGATTCTAGCCCCGGCAACGAAGGGCGGCGACCTACTCAGCC

ATCGGGCACGATCTTTATCGATCCCGTGAGAGGAAGTCGACGTTA
                                                2250
TAGCCCGTGCTAGAAATAGCTAGGGCACTCTCCTTCAGCTGCAAT
```

GGGAGGGTGTGGACACTGTCCACATCCCCGCTTACGCCGCCTCC 2295
CCCTCCCACACCTGTGACAGGTGTAGGGGGCGAATGCGGCGGAGG

BssSI

CTCCACGAGGTCGAGAAGTTCATGGACCGCCCCGACATCATCTTG 2340
GAGGTGCTCCAGCTCTTCAAGTACCTGGCGGGGCTGTAGTAGAAC

ACGAGGAAGTGTCGGAACCCCATGCTGAAGCATGCTCGGGAACTC 2385
TGCTCCTTCACAGCCTTGGGGTACGACTTCGTACGAGCCCTTGAG

GGTTTATTCGAGGAGGTCTTCGAGAGTCGGGTGGGAGGTGGAATC 2430
CCAAATAAGCTCCTCCAGAAGCTCTCAGCCCACCCTCCACCTTAG

CTATGGGCTTCCCTTAATGGAAAAGCCCTTGTGGAATCCCACTCC 2475
GATACCCGAAGGGAATTACCTTTTCGGGAACACCTTAGGGTGAGG

CCCTCGATCCTCCAAGTCTCCCGGAATCTCCGGCGATCCCTTGCG 2520
GGGAGCTAGGAGGTTCAGAGGGCCTTAGAGGCCGCTAGGGAACGC

TGCCCCAGTGGGGGTTTCCTCAGACCATCTGCCCCTATAGGGAAG 2565
ACGGGGTCACCCCCAAAGGAGTCTGGTAGACGGGGATATCCCTTC

CTGGTCCAACGACATACGTTGCCTCGGGGAACGGTCTGGTTCTTA 2610
GACCAGGTTGCTGTATGCAACGGAGCCCCTTGCCAGACCAAGAAT

GAAAGTTCCGCGACCGACTCGGCCCGTCAGGGAGGGATGGGTCTG 2655
CTTTCAAGGCGCTGGCTGAGCCGGGCAGTCCCTCCCTACCCAGAC

CCACCACCACCACCGCCGCCTCTAGGCGGAGGTGGGATGGCGGGT 2700
GGTGGTGGTGGTGGCGGCGGAGATCCGCCTCCACCCTACCGCCCA

CCGCCACCACCTCCTTTCATGGGCCTTCGCCCCGAATCATCAGTT 2745
GGCGGTGGTGGAGGAAAGTACCCGGAAGCGGGGCTTAGTAGTCAA

```
CCTACTAGTGTTCCATTTACACCTTCAATGTTCTCTGAACGCCTT
                                                 2790
GGATGATCACAAGGTAAATGTGGAAGTTACAAGAGACTTGCGGAA

GCTGCGTTAGAGTCATTGTTCGGCCGCCCGCCACCTTCATGATGG
                                                 2835
CGACGCAATCTCAGTAACAAGCCGGCGGGCGGTGGAAGTACTACC

CAGAACGGGGAAACTCGTTCCGCTCAGGCACGGTATAATTACCGG
                                                 2880
GTCTTGCCCCTTTGAGCAAGGCGAGTCCGTGCCATATTAATGGCC

           End (2891)
GCCTGAGCCCC    3′
                 2891
CGGACTCGGGG    5′
```

***Start*** (0)

```
5′  GAAAAATAAAGAAATGACGAAGCCAACCCAAGTATTAGTTAGATC   45
3′  CTTTTTATTTCTTTACTGCTTCGGTTGGGTTCATAATCAATCTAG

    CGTCAGTATATTATTTTTCATCACATTACTACACCTAGTCGTAGC   90
    GCAGTCATATAATAAAAAGTAGTGTAATGATGTGGATCAGCATCG

                                              M1-F
                                               ATT
    GCTGAACGATGTGGCCGGTCCTGCAGAAACAGCACCAGTGTCATT   135
    CGACTTGCTACACCGGCCAGGACGTCTTTGTCGTGGTCACAGTAA

          M1-F
    ACTACCTCGTGAAGCGC
    ACTACCTCGTGAAGCGCCGTGGTATGACAAGATCTGGGAAGTAAA   180
    TGATGGAGCACTTCGCGGCACCATACTGTTCTAGACCCTTCATTT

    AGATTGGCTATTACAGCGTGCCACAGATGGCAATTGGGGCAAGTC   225
    TCTAACCGATAATGTCGCACGGTGTCTACCGTTAACCCCGTTCAG

    GATCACCTGGGGTTCATTCGTAGCGAGCGATGCAGGTGTAGTAAT   270
    CTAGTGGACCCCAAGTAAGCATCGCTCGCTACGTCCACATCATTA

    CTTTGGTATCAATGTGTGTAAGAACTGCGTGGGTGAGCGTAAGGA   315
    GAAACCATAGTTACACACATTCTTGACGCACCCACTCGCATTCCT

                  M1-F2
           AGTACGGACTGCGGCAAGC
    TGATATCAGTACGGACTGCGGCAAGCAAACACTTGCTTTACTAGT   360
    ACTATAGTCATGCCTGACGCCGTTCGTTTGTGAACGAAATGATCA
```

CAGCATTTTTGTAGCAGTTACATCCGGCCATCATCTTATATGGGG 405
GTCGTAAAAACATCGTCAATGTAGGCCGGTAGTAGAATATACCCC

CGTCAATGTAGGCCGGTAGT
M1-R

TGGTAATAGGCCGGTGTCGCAGTCAGATCCTAATGGCGCTACCGT 450
ACCATTATCCGGCCACAGCGTCAGTCTAGGATTACCGCGATGGCA

TGCTCGTCGTGACATTTCTACTGTCGCAGACGGGGATATTCCACT 495
ACGAGCAGCACTGTAAAGATGACAGCGTCTGCCCCTATAAGGTGA

GGACTTTAGTGCGTTGAACGACATATTAAATGAACATGGTATTAG 540
CCTGAAATCACGCAACTTGCTGTATAATTTACTTGTACCATAATC

TATACTCCCAGCTAACGCATCACAATATGTCAAAAGATCAGACAC 585
ATATGAGGGTCGATTGCGTAGTGTTATACAGTTTTCTAGTCTGTG

AGCCGAACACACGACAAGTTTTGTAGTGACCAACAACTACACTTC 630
TCGGCTTGTGTGCTGTTCAAAACATCACTGGTTGTTGATGTGAAG

CGGCTTGTGTGCTGTTCAAAA
M1-R2

TTTGCATACCGACCTGATTCATCATGGTAATGGAACATATACCAC 675
AAACGTATGGCTGGACTAAGTAGTACCATTACCTTGTATATGGTG

GTTTACCACACCTCACATTCCAGCAGTGGCCAAGCGTTATGTTTA 720
CAAATGGTGTGGAGTGTAAGGTCGTCACCGGTTCGCAATACAAAT

TCCTATGTGCGAGCATGGTATCAAGGCCTCATACTGTATGGCCCT 765
AGGATACACGCTCGTACCATAGTTCCGGAGTATGACATACCGGGA

TAATGATGCCATGGTGTCGGCTAATGGTAACCTGTATGGACTAGC 810
ATTACTACGGTACCACAGCCGATTACCATTGGACATACCTGATCG

```
AGAAAAGCTGTTTAGTGAGGATGAGGGACAATGGGAGACGAATTA
                                                 855
TCTTTTCGACAAATCACTCCTACTCCCTGTTACCCTCTGCTTAAT

CTATAAATTGTATTGGAGTACTGGCCAGTGGATAATGTCGATGAA
                                                 900
GATATTTAACATAACCTCATGACCGGTCACCTATTACAGCTACTT

GTTTATTGAGGAAAGTATTGATAACGCCAATAATGACTTTGAAGG
                                                 945
CAAATAACTCCTTTCATAACTATTGCGGTTATTACTGAAACTTCC

CTGTGACACAGGCCACTAGGGCATCGTGTCTGACCTCTGATGCGA
                                                 990
GACACTGTGTCCGGTGATCCCGTAGCACAGACTGGAGACTACGCT

TAACTCGACCCTACAGAGCACCGGGCTATATATAATATAGTAGGC
                                                 1035
ATTGAGCTGGGATGTCTCGTGGCCCGATATATATTATATCATCCG

ACAAAATAAAATAAAAATTAAAAAAAAAAAAAAAAAAAAAAAAAA
                                                 1080
TGTTTTATTTTATTTTTAATTTTTTTTTTTTTTTTTTTTTTTTTT

AAAAAAAAAAAAAAAAAAAAAAAAAAAAAAAAAAAAAAAAAAAAA
                                                 1125
TTTTTTTTTTTTTTTTTTTTTTTTTTTTTTTTTTTTTTTTTTTTT

AAAAAAAAAAAAAAAAAAAAAAAAAAAAAAAAAAAAAAAGAAAAG
                                                 1170
TTTTTTTTTTTTTTTTTTTTTTTTTTTTTTTTTTTTTTTCTTTTC

AGAGAGAAGAAGAAGAAGAAAAAGAAAAAACAAAAGAAACAGAAA
                                                 1215
TCTCTCTTCTTCTTCTTCTTTTTCTTTTTTGTTTTCTTTGTCTTT

AAGAGAGAACAGGACAACAAACGCAACAAAACACAAACACAAGCA
                                                 1260
TTCTCTCTTGTCCTGTTGTTTGCGTTGTTTTGTGTTTGTGTTCGT

CACTCACCTTGAGTCTAACTGGTGGCACGCAGCATATCTCACCCT
                                                 1305
GTGAGTGGAACTCAGATTGACCACCGTGCGTCGTATAGAGTGGGA
```

```
GAGACTAACTGGCGGCAGGCGACCGTGAGCATACAGCATGCCCCA
                                                 1350
CTCTGATTGACCGCCGTCCGCTGGCACTCGTATGTCGTACGGGGT

CTCGATTCGAGACGCGATTCGCGCTCGTAGGTATCGAGCGGCTAC
                                                 1395
GAGCTAAGCTCTGCGCTAAGCGCGAGCATCCATAGCTCGCCGATG

GTTGAGCTATTATGGCAGTGACATGCGATTCGCGCACTGCCAAGA
                                                 1440
CAACTCGATAATACCGTCACTGTACGCTAAGCGCGTGACGGTTCT

TCAGCTCAGCAAAGTTAAGACCAGTATCGGATATGGTAGACTACT
                                                 1485
AGTCGAGTCGTTTCAATTCTGGTCATAGCCTATACCATCTGATGA

ACAATTCGCACAGGTATGAGATTCTCAGTCTAGTGTATGGATGAG
                                                 1530
TGTTAAGCGTGTCCATACTCTAAGAGTCAGATCACATACCTACTC

TAGTTGAGCCAATGAATCTAGGGTTTAAATTACTATGCATTGACA
                                                 1575
ATCAACTCGGTTACTTAGATCCCAAATTTAATGATACGTAACTGT

TATAGCAGGTACAAGCGTAGATAATACTTACTAGGCCCCAGCCGG
                                                 1620
ATATCGTCCATGTTCGCATCTATTATGAATGATCCGGGGTCGGCC

TACACCCTGTATTGAATAAATACGACTATTTGGCCAGGTCTGGAC
                                                 1665
ATGTGGGACATAACTTATTTATGCTGATAAACCGGTCCAGACCTG

GGGGCAGTCGAATTACTAGGTTGAGCACACACACGTGAATCACAC
                                                 1710
CCCCGTCAGCTTAATGATCCAACTCGTGTGTGTGCACTTAGTGTG

AACATAACAGTGTAGGAACATAATGTGCCATTCGTAGTCTGAGAC
                                                 1755
TTGTATTGTCACATCCTTGTATTACACGGTAAGCATCAGACTCTG

GCCGCTAGCCTGGTTTAATGCAACAGCATAGAAGAAACACACATC
                                                 1800
CGGCGATCGGACCAAATTACGTTGTCGTATCTTCTTTGTGTGTAG
```

***End*** (1801)

A 3′
1801
T 5′

Sequence: M2.dna (Linear / 1163 bp)
Enzymes: Unique 6+ Cutters (35 of 678 total)
Primers: 2 total

Unique Cutters **Bold**

***Start*** (0)

M2-F2
GAGCTG
5′ GAAAAAATGAAAGAGACTACCACCAGCCTGGTGCAAGACGAGCTG 45
3′ CTTTTTTACTTTCTCTGATGGTGGTCGGACCACGTTCTGCTCGAC

M2-F2
ACACTAGGTGAGCC
ACACTAGGTGAGCCGGCCACCCGAGCAAGGATGTGCGTACGTCTA 90
TGTGATCCACTCGGCCGGTGGGCTCGTTCCTACACGCATGCAGAT

TTACGTTTTTTCATAGGTCTGACTATAACCGCATTTATTATAGCA 135
AATGCAAAAAAGTATCCAGACTGATATTGGCGTAAATAATATCGT

GCCTGTATTATTAAAAGTGCGACAGGCGGTTCGGGATATTCTAAT 180
CGGACATAATAATTTTCACGCTGTCCGCCAAGCCCTATAAGATTA

GCAGTTGCTGTTCGGGGAGAAGCGGACACCCCTTCCACAATTGTG 225
CGTCAACGACAAGCCCCTCTTCGCCTGTGGGGAAGGTGTTAACAC
CAAGCCCCTCTTCGCCTGTG
M2-R

GGCCAGCTCGTCGAGCGTGGCGGCTTCCAAGCTTGGGCAGTGGGG 270
CCGGTCGAGCAGCTCGCACCGCCGAAGGTTCGAACCCGTCACCCC

GCTGGTATCTATTTGTTTGCCAAGATAGCATATGATACATCTAAG 315
CGACCATAGATAAACAAACGGTTCTATCGTATACTATGTAGATTC

GTTACCGCAGCTGTATGTAATCCGGAGGCGCTCATTGCTATCACA 360
CAATGGCGTCGACATACATTAGGCCTCCGCGAGTAACGATAGTGT

TCGTATGTGGCATATGCCCCTACACTGTGTGCTGGTGCATACGTT 405
AGCATACACCGTATACGGGGATGTGACACACGACCACGTATGCAA

```
ATTGGTGCCATGAGTGGGGCAATGTCGGCGGGCCTTGCTCTGTAT
                                               450
TAACCACGGTACTCACCCCGTTACAGCCGCCCGGAACGAGACATA

GCCGGTTACAAAGGATGGCAGTGGAGCGGCCCCGGGGGCATGGCA
                                               495
CGGCCAATGTTTCCTACCGTCACCTCGCCGGGGCCCCCGTACCGT

GAGAGAGAGGACGTGGCCTCTTTTTATTCACCACTCCTGAACAAC
                                               540
CTCTCTCTCCTGCACCGGAGAAAAATAAGTGGTGAGGACTTGTTG

ACTCTGTACGTGGGTGGGGACCACACTGCAGACTACGACAGTGAA
                                               585
TGAGACATGCACCCACCCCTGGTGTGACGTCTGATGCTGTCACTT

TTGGCTACTATATTAGGTAGCGTATATAATGATGTGGTCCACCTG
                                               630
AACCGATGATATAATCCATCGCATATATTACTACACCAGGTGGAC

GGGGTGTATTACGATAACAGCACTGGAATTGTCAAGAGGGATTCG
                                               675
CCCCACATAATGCTATTGTCGTGACCTTAACAGTTCTCCCTAAGC

AGACCTAGCATGACCTCATGGACGGTGTTGCATGACAACATGATG
                                               720
TCTGGATCGTACTGGAGTACCTGCCACAACGTACTGTTGTACTAC

ATAACATCATACCATAGGCCAGACCAGCTGGGCGCAGCCGCGACA
                                               765
TATTGTAGTATGGTATCCGGTCTGGTCGACCCGCGTCGGCGCTGT

GCCTACAAAGCTTATGCCACAAACACAACACGGGTCGGTAAGAGG
                                               810
CGGATGTTTCGAATACGGTGTTTGTGTTGTGCCCAGCCATTCTCC

CAGGACGGTGAGTGGGTGTCATACTCGGTCTACGGTGAGAATGTT
                                               855
GTCCTGCCACTCACCCACAGTATGAGCCAGATGCCACTCTTACAA

GACTATGAAAGATACCCTGTAGCACATCTGCAAGAGGAGGCCGAC
                                               900
CTGATACTTTCTATGGGACATCGTGTAGACGTTCTCCTCCGGCTG
```

```
GCGTGTTACGAGAGTTTAGGTAATATGATTACGAGCCAGGTACAG
CGCACAATGCTCTCAAATCCATTATACTAATGCTCGGTCCATGTC    945

CCCTGTACTCAGAGAGAATGTTATGCTATGGATCAGAAAGTATGC
GGGACATGAGTCTCTCTTACAATACGATACCTAGTCTTTCATACG    990

GCAGCTGTCGGCTTCTCATCAGATGCGGGTGTTAACTCCGCAATG
CGTCGACAGCCGAAGAGTAGTCTACGCCCACAATTGAGGCGTTAC    1035

GTCGGTGAGGCCTACTTCTATGCCTATGGTGGGGTTGATGGTGAA
CAGCCACTCCGGATGAAGATACGGATACCACCCCAACTACCACTT    1080

TGTGACAGCGGCTAGGATAGGATATAAATAATATATTAATAAAAC
ACACTGTCGCCGATCCTATCCTATATTTATTATATAATTATTTTG    1125

                                      End (1163)
AAAATAATAAAAATATAAAAAAAAAAAAAAAAAAAAAA    3′
TTTTATTATTTTTATATTTTTTTTTTTTTTTTTTTTTT    5′   1163
```

Sequence: m28.dna (Linear / 1090 bp)
Enzymes: Unique 6+ Cutters (38 of 678 total)
Primers: 4 total

Unique Cutters **Bold**

***Start*** (0)

```
5′ gaaaaaatttgaatggagagcgtttcctcattatttaacattttt   45
3′ cttttttaaacttacctctcgcaaaggagtaataaattgtaaaaa

   tcaacaatcatggttaactataaatcgttagttctagcactatta   90
   agttgttagtaccaattgatatttagcaatcaagatcgtgataat

                          M28-F
                    aatatgcacggggtatgccg
   agtgtttcaaatctcaaatatgcacggggtatgccgacatctgag   135
   tcacaaagtttagagtttatacgtgccccatacggctgtagactc

   agagaggagggcttagaagaacgtgacttcagtgctgctacttgc   180
   tctctcctcccgaatcttcttgcactgaagtcacgacgatgaacg

   gtactgatgggcgcagaagtaggctcatggggaatggtttatagt   225
   catgactacccgcgtcttcatccgagtaccccttaccaaatatca

   ggtcagaaggtcgagagttggatcctctacgttctgactggcatt   270
   ccagtcttccagctctcaacctaggagatgcaagactgaccgtaa

   actacgatgagcgcaatcgttgacgaaattgactattatgcgtca   315
   tgatgctactcgcgttagcaactgctttaactgataatacgcagt

                          M28-F2
                    ttgtgggtgagaactcaggg
   catatgccactgagtgttgtgggtgagaactcagggctacaaatc   360
   gtatacggtgactcacaacacccactcttgagtcccgatgtttag
```

```
gttcgtgataccatagtaaccttggttatggctggcctgacagca    405
caagcactatggtatcattggaaccaataccgaccggactgtcgt
                                 ccggactgtcgt
                                     M28-R

tcagctaacaaggtaatcagtaagactgaaaacgcagagaatata    450
agtcgattgttccattagtcattctgacttttgcgtctcttatat
agtcgatt
  M28-R

caatcgcgtagtcttataccgggtctgcttagtatggattataac    495
gttagcgcatcagaatatggcccagacgaatcatacctaatattg

agtactcatactatggcgattaatttggaagacgtattctcggag    540
tcatgagtatgataccgctaattaaaccttctgcataagagcctc

ctcggctgggacatcgatactagtgatagctctggtttatacaaa    585
gagccgaccctgtagctatgatcactatcgagaccaaatatgttt

cgtgacgataattctgtcactctgcacctaggggacgtacctgct    630
gcactgctattaagacagtgagacgtggatcccctgcatggacga
                         cgtggatcccctgcatggac
                                M28-R2

ctaggcaccagtaacactatcatacctaacgctgtcatgcaaata    675
gatccgtggtcattgtgatagtatggattgcgacagtacgtttat

tataataacgcatcatttgctttcggttttgcacctcatagcaac    720
atattattgcgtagtaaacgaaagccaaaacgtggagtatcgttg

ggtaattctacaggcttgcagaaacgagctagtattgatgatgcg    765
ccattaagatgtccgaacgtctttgctcgatcataactactacgc
```

```
gtgtggttacaatctgcatacggaatagcttatagtgcctggata
                                                  810
cacaccaatgttagacgtatgccttatcgaatatcacggacctat

ggctctgagaatgtgggttcctatgatcagcatctagctgaagct
                                                  855
ccgagactcttacacccaaggatactagtcgtagatcgacttcga

aacggtatggctaactactggacgtccgagtgttctaagtacaat
                                                  900
ttgccataccgattgatgacctgcaggctcacaagattcatgtta

ggtgtcatctggggtgacgaatcagacgcctgcggtaactggcta
                                                  945
ccacagtagaccccactgcttagtctgcggacgccattgaccgat

gcatcacagcgtttagacatagtgagtcactcaacaggcaattac
                                                  990
cgtagtgtcgcaaatctgtatcactcagtgagttgtccgttaatg

tacagagacgttaacctctgtggtgacgacgaggcaaggtgccac
                                                  1035
atgtctctgcaattggagacaccactgctgctccgttccacggtg

gatgagctacgctaatagtccagaccgacgcttcttagttatgat
                                                  1080
ctactcgatgcgattatcaggtctggctgcgaagaatcaatacta

          End (1090)
caggctgtga    3′
                 1090
gtccgacact    5′
```

***Start*** (0)

```
5′ gaaaaaatatataaacttgtatggtgcctcaagcagtactagtag
3′ ctttttatatatttgaacataccacggagttcgtcatgatcatc     45

   atttattttttcgctttctaacttcctattgtttaccaattacta
   taaataaaaaagcgaaagattgaaggataacaaatggttaatgat     90

                                      Mlus-F2
                                      gttctggactg
   tcacttacttagttacgggtcatgcatttaaaaagttctggactg
   agtgaatgaatcaatgcccagtacgtaaatttttcaagacctgac     135

  Mlus-F2
   tgtttgc
   tgtttgctatacttattaactaccgtcttatcactggcggccgct
   acaaacgatatgaataattgatggcagaatagtgaccgccggcga     180

   acaatagtaccaccaacagttgataaccatactgttaccattatt
   tgttatcatggtggttgtcaactattggtatgacaatggtaataa     225

                Mlus-F1
          ggcactgtcctgcctgttatag
   gatagcaatggcactgtcctgcctgttatagctgctagactcaat
   ctatcgttaccgtgacaggacggacaatatcgacgatctgagtta     270

   ctagattattttgaagaacagaacacctctatcatcaagcgggat
   gatctaataaaacttcttgtcttgtggagatagtagttcgcccta     315

                                      Mlus-F2-alt
                                     gtgttaccttg
   gctaccgttgatgactggctgactgaggtagtagtgttaccttg
   cgatggcaactactgaccgactgactccatcatcacaatggaaac     360
```

Mlus-F2-alt
gctgacgc
gctgacgctcaacaagttgatcctaatactaacttagcgaagcgc 405
cgactgcgagttgttcaactaggattatgattgaatcgcttcgcg

actaacgttgaaggtgctctatattggctagtcggttcaggtcaa 450
tgattgcaacttccacgagatataaccgatcagccaagtccagtt

tgtgtgtatgagtactgggatattgctgatggcgtgtggcaagcc 495
acacacatactcatgaccctataacgactaccgcacaccgttcgg
cgactaccgcacaccgtt
Mlus-R1

ggctgggacatctatcgagcaacttcaaccgataattgccaagta 540
ccgaccctgtagatagctcgttgaagttggctattaacggttcat
cggttcat
Mlus-R2

Mlus-F3
ggat
attatgggacataagaattccttctactataaatattatgcggat 585
taataccctgtattcttaaggaagatgatatttataatacgccta
taataccctg
Mlus-R2

Mlus-F3
gatgggaaatgtag
gatgggaaatgtagttctacagtcaaacaaaagacaatcgcaggt 630
ctaccctttacatcaagatgtcagtttgttttctgttagcgtcca

gctttgcaggccgcggtccgccagctagaagggaaccaactgtgt 675
cgaaacgtccggcgccaggcggtcgatcttcccttggttgacaca
gcggtcgatcttcccttggt
Mlus-R2alt

aacaactacctcttccatgtagatcaccacggcacttggcatggt 720
ttgttgatggagaaggtacatctagtggtgccgtgaaccgtacca

gacgttattatcggagcatcaatctcagcttggttcactaacgct 765
ctgcaataatagcctcgtagttagagtcgaaccaagtgattgcga

aaatggtccgactataaaggatgggttgatgctggatgtagtcaa 810
tttaccaggctgatatttcctacccaactacgacctacatcagtt

ctacagtcaccttacacatgctctagttagcacatttgtttaaga 855
gatgtcagtggaatgtgtacgagatcaatcgtgtaaacaaattct
gtggaatgtgtacgagatca
Mlus-R3

gacagaacactgagatagattaaccaattaaataaggataggtta 900
ctgtcttgtgactctatctaattggttaatttattcctatccaat

aacacgtaaacagatactctaaaaaaaaaaaaaaaaaaaaaaaaa 945
ttgtgcatttgtctatgagattttttttttttttttttttttttt

aaaaaaaaaaaaaaaaaaaaaaataaataaagaaagaaaagaaga 990
tttttttttttttttttttttttatttatttctttcttttcttct

aataaaagaagaaagaaaaggaagagaagaaagaaagcactttac 1035
ttattttcttctttcttttccttctcttctttctttcgtgaaatg

atgatcaatcatgcaatgtactactcaccttgagtataactggtg 1080
tactagttagtacgttacatgatgagtggaactcatattgaccac

```
gcgtcttaggactgaggctcaggccttgatgaaccgtcaaagtaa
                                               1125
cgcagaatcctgactccgagtccggaactacttggcagtttcatt

ctaattcagcctctgacgaattcatcatttattaatacgatgcga
                                               1170
gattaagtcggagactgcttaagtagtaaataattatgctacgct

atatattcttaaatataataaaaaaaaaaaaaaaaaaaaaaaaaa
                                               1215
tatataagaatttatattattttttttttttttttttttttttttt

aaaaaaaaaaaaaaaaataaatataaatataaataatataatgaa
                                               1260
tttttttttttttttttatttatatttatatttattatattactt

tacaacaaaacaactagacaaggagacaggcataggcaggaggca
                                               1305
atgttgttttgttgatctgttcctctgtccgtatccgtcctccgt

                                       Mlus-FW
                                       gctaaa
gagtggccggtacacgggtcaccaacagcatggtcattggctaaa
                                               1350
ctcaccggccatgtgcccagtggttgtcgtaccagtaaccgattt

   Mlus-FW
acgagttggttgggc
acgagttggttgggccaaaggacacttatatctgcacatgcggcg
                                               1395
tgctcaaccaacccggtttcctgtgaatatagacgtgtacgccgc

cctacacgctaataggaacagacgatccgtcgctccaccttagtg
                                               1440
ggatgtgcgattatccttgtctgctaggcagcgaggtggaatcac

gtagtaactgtgcgtgctgagcctacaacgatctccattatagtg
                                               1485
catcattgacacgcacgactcggatgttgctagaggtaatatcac

ccacgtcaccattgactaaatgagttactctagtccatgggacac
                                               1530
ggtgcagtggtaactgatttactcaatgagatcaggtaccctgtg
```

```
tcttatttgcacacgcggcatctacacgccaataggaacagacga 1575
agaataaacgtgtgcgccgtagatgtgcggttatccttgtctgct

ttcgtcgctccactctggcagtagcgactgtgcgtgcaaagcctg 1620
aagcagcgaggtgagaccgtcatcgctgacacgcacgtttcggac

taacaacctccactacaaccccacattaccagtaaatatgaatcc 1665
attgttggaggtgatgttggggtgtaatggtcatttatacttagg

tcccggttaactggtttccactatcactctgagagagtattagcc 1710
agggccaattgaccaaaggtgatagtgagactctctcataatcgg
                                            g  Mlus-RV

aaagtctggacacgccctgtagcccagatgagcgaagctgataca 1755
tttcagacctgtgcgggacatcgggtctactcgcttcgactatgt
tttcagacctgtgcgggaca
Mlus-RV

gcctgatgttgatgtaacactgctgagccgtgtcttaacgcgggg 1800
cggactacaactacattgtgacgactcggcacagaattgcgcccc

cccagccttgaggtaccattacgttgtgcgattagtgtgatagcc 1845
gggtcggaactccatggtaatgcaacacgctaatcacactatcgg

tgtccaggagtgtagtaacatacactagacctgaaccagtgtggt 1890
acaggtcctcacatcattgtatgtgatctggacttggtcacacca

atacatactgtgagtctcggcgtatactctcagcaaggtggccta 1935
tatgtatgacactcagagccgcatatgagagtcgttccaccggat

catcactatgtttagtacttcagcctaaggcgattagggattctg 1980
gtagtgatacaaatcatgaagtcggattccgctaatccctaagac
```

```
gtacaatgtatggttgaacatacggggcaagatgattggagcgat
                                                        2025
catgttacataccaacttgtatgccccgttctactaacctcgcta

             End (2033)
ccagatgc      3′
                 2033
ggtctacg      5′
```
