# Supplementary material for: RNA viruses, M satellites, chromosomal killer genes, and killer/nonkiller phenotypes in the 100-genomes S. cerevisiae strains
Source: G3 (Bethesda). 2023 Jul 27;13(10):jkad167. doi: 10.1093/g3journal/jkad167 (PMC10542562; doi:10.1093/g3journal/jkad167)
Supplement: jkad167_Supplementary_Data [file jkad167_supplementary_data.zip › Figure_S5_G3-2023-404116.pdf]

Fig S5

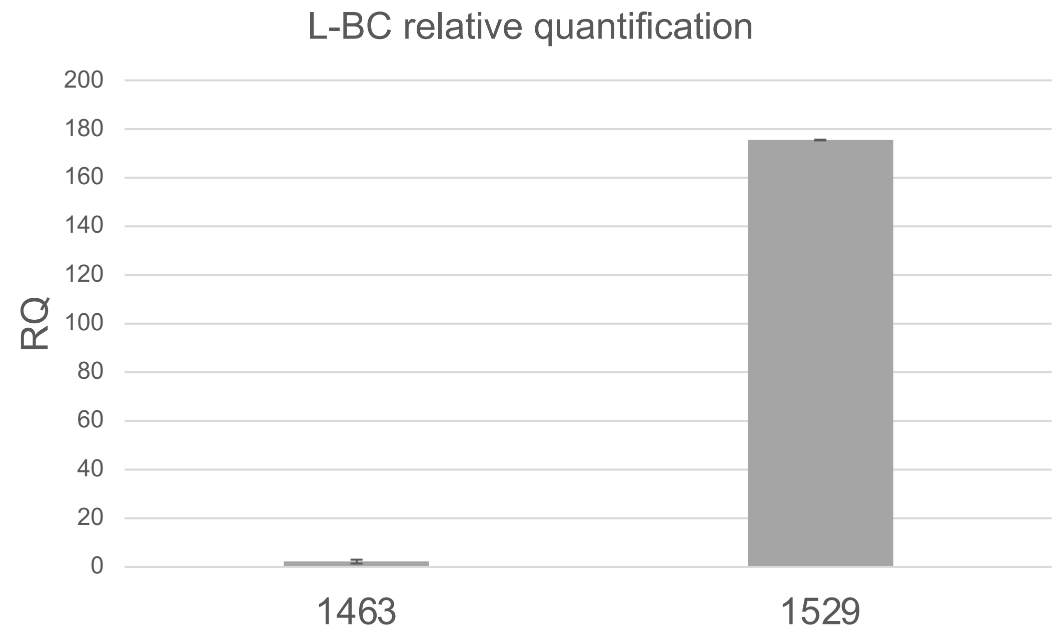

Sample	Target	Adjusted EqCq Mean	ΔEqCq Mean	Δ EqCq SD	ΔΔEqCq	RQ
996	L-BC	30.62568	10.64394	0.208972	0	1
996	UBC6	19.98174				
1463	L-BC	32.11738	9.526344	0.79957	-1.11759	2.169847
1463	UBC6	22.59104				
1529	L-BC	25.20431	3.188781	0.15369	-7.45516	175.4792
1529	UBC6	22.01553				
