# Supplementary material for: RNA viruses, M satellites, chromosomal killer genes, and killer/nonkiller phenotypes in the 100-genomes S. cerevisiae strains
Source: G3 (Bethesda). 2023 Jul 27;13(10):jkad167. doi: 10.1093/g3journal/jkad167 (PMC10542562; doi:10.1093/g3journal/jkad167)
Supplement: jkad167_Supplementary_Data [file jkad167_supplementary_data.zip › Supplemental_Material_Legends_G3-2023-404116.docx]

Supplemental Material legends:

Supplemental Figure legends:

Figure S1. Sequence maps (NCBI Accession numbers) of L-A (NC_003745), L-BC (NC_001641), M1 (NC_001641),M2 (X54154), M28 (KJ796682), Mlus (GU723494), 20S (NC_004051), and 23S (NC_004050) found in the 100-genome strains. Representative sequences are shown in map view, with primer binding sites highlighted.

Figure S2: YJM195 gel and PCR analysis. **(**A) Gel analysis of L-A^+^ and L-A cured (L-A^0^) RNA after treatment with DNase, showing L-A and L-BC (4.7kb) along with the 3kb band. Lower bands likely represent ribosomal RNA. (B) PCR analysis of the samples from panel A with 3 independent primer pairs (1-3) to confirm the presence/absence of L-A. (C) Treatment of samples from panel A with various nucleases to confirm identity of M28. PCRs were done to test for the presence/absence of L-BC (L), M28 (M) and genomic DNA (g). The M28 amplicon, which is present in both L-A^+^ and L-A^0^, originates from the genomic M28 cDNA. In both L-A^+^ and L-A^0^, the M28 amplicon appears to be reduced by DNase alone; is unaffected by ss-specific RNaseI_f_ alone; is unaffected by ds-specific RNaseIII alone; and is almost completely eliminated by both double digests (DNase + RNaseI_f_ and DNase + RNaseIII). The residual M28 amplicon in both double digests may originate from bi-directional transcription (possibly reverse transcriptase-mediated) of the Ty-embedded M28 cDNA. Primers for PCR analysis L-BC F2+R2 (L-BC), M28 F+R (M1), MAT+HML+HMR (genomic DNA)

Figure S3: Analysis of YJM195 and YJM681. (A) Shown are isogenic strain pairs of native and L-A-cured strains. (B) Test for killer activities of strains representing K1, K2 and K28 killers on YJM195 and YJM681 L-A^+^ M^+^ and L-A^0^ M^0^ lawns

Figure S4: Analysis of YJM1133 and YJM1419. (A) Representative gel showing isogenic sets of L-A^+^ M^+^, L-A^+^ M^0^ and L-A^0^ M^0^ strains. The greatly elevated level of L-A in the L-A^+^ M^0^ derivatives has been previously described (Ball *et al.* 1984). (B) Corresponding PCRs from cDNAs of RNA derived from the indicated strains, probing for presence/absence of L-A (A), L-BC (B) or M (using Mlus-specific primers for YJM1133 samples and M1-specific primers for YJM1419). All samples were treated with DNase prior to gel and PCR analysis. Primers for PCR analysis Primers for PCR analysis were LA-F4 + LA-R5 (L-A), LBC-F2 + LBC-R2 (L-BC), Mlus-F2 + Mlus-R2 (Mlus), M1-F + M1-R (M1).

Figure S5: Comparison of L-BC levels in YJM1463 and YJM1529 by real time PCR. L-BC amounts were measured relative to UBC6 mRNA amounts and quantification was performed using ΔΔCt method, using YJM996 as a calibrator sample. Primers for PCR L-BC qF2 + L-BC qR2 (L-BC), UBC6-qF + UBC6-qR (UBC6).

Adjusted EqCq mean: Arithmetic average of Cq means from technical replicates, adjusted to a maximum allowed Cq mean of 40.

ΔΔ*CqEq* Fold change between EqCq mean between experimental samples and calibrator sample

RQ Relative gene expression level calculated as 2^-ΔΔ^*^CqEq^*. For further details, refer to <https://assets.thermofisher.com/TFS-Assets/LSG/manuals/MAN0018747_RelativeQuantificatAnalysisModule_QSDASWv2_UG.pdf>

Figure S6: Chromosomal Killer phenotypic analysis:

A) Killing of *C. glabrata* is *KHS1*-dependent.

*C. glabrata* cells were plated onto MB (pH 4.7, pH 6.0) media. YJM1893 (*HO* *khr1*∆::hphMX4 *khs1*∆::natMX4), YJM1894 (*HO* *khr1*∆::hphMX4 *KHS1*), YJM1895 (*HO* *KHR1* *KHS1*), and YJM1896 (*HO* *KHR1* *khs1*∆::natMX4), which are isogenic with YJM189 (YJM189 *HO* (self-diploidized) *KHR1* *KHS1*), were patched onto the *C. glabrata* lawns. No strains killed at pH 6.0, the pH permissive for Khr1 toxin activity, and non-permissive for Khs1 toxin activity, consistent with the YJM189 background not producing functional Khr1 toxin. *KHS1* strains killed and *khs1*∆ strains did not kill *C. glabrata* at pH 4.7. In the YJM189 genetic background, killing of *C. glabrata* at pH 4.7 was entirely *KHS1*-dependent.

B) *KEX2*, but not *KEX1*, is required for Khs1-mediated killing of *C. glabrata*.

Strains isogenic with YJM189 *HO* (self-diploidized) *KEX1* *KEX2* *KHS1* were constructed by making *KEX1*/*kex1*∆ and *KEX2*/*kex2*∆ derivatives, which were sporulated and dissected. Four segregants (one tetrad) from each derivative were tested for their Khs1 killer activity. While *KEX1* is not required, *KEX2* is required for Khs1-mediated killing of *C. glabrata*, consistent with Kex2p being required for Khs1 toxin processing.

C) *KHS1* encodes both Khs1 toxin and Khs1 antitoxin.

YJM189 *KHS1* kills the isogenic strain YJM1896 *khs1*∆, consistent with *KHS1* encoding both Khs1 toxin and Khs1 antitoxin.

Figure S7: Manhattan plot of Fast GWAS of *C. glabrata* killing by M^0^ 100-genomes strains.

Derived from Table S7. The sole significant association corresponds to *KHS1*.

Figure S8: Manhattan plot of Fast GWAS of YJM189 *KHS1* killing of M^0^ 100-genomes strains.

Derived from Table S8.

Supplemental Table legends:

Table S1: List of strains used in this study.

Table S2: List of plasmids and primers used in this study.

Table S3: Summary of gel analysis and PCR genotypes for L-A, L-BC, 20S, 23S and M-satellites for the 100 genomes, as well as L-BC levels. L-BC level and PCR genotypes were determined as described in Methods. YJM1463 and YJM1529 could not be cured of L-A to assess L-BC level by gel. For these two strains, L-BC levels were determined by RT-qPCR, as described in Methods.

Table S4: Summary of correlations of virus genotypes with population, other viruses, L-BC levels, 2µ plasmid, as well as the mitochondrial introns SCE1 and COX1-intron 1. List of high throughput phenotypes tested for which there were no associations with viruses or M satellites.

Table S5: Summary of killer phenotype assays for the 100-genomes detailing effects of L-A and/or M-killing.

Killer phenotypes: separate zone size (qualitative score: 2, 1, 0.5, 0) and zone turbidity (qualitative score: 2 = clear; 1 = turbid; 0.5 = blue ring; 0) phenotype scores for each strain; yellow fill: M-independent killing of YJM1447 and YJM1573.

Column A: lists strain lawns; for lawns, M1- and M2-containing strains were excluded, using instead their isogenic cured derivatives. For other genetic backgrounds, both parental L-A^+^ (M^+^) and isogenic L-A^0^ (M^0^) derivatives were tested.

Row 1/Columns B - AK: lists L-A^+^ M^+^ strains and (except for EX229 and MS300c) control isogenic L-A^0^ M^0^ strains.

The strains listed in Row 1/Columns B - S were patched onto each of the lawns to determine the ability of the strains to kill the lawns.

For L-A^+^ (M^+^) vs. L-A^0^ (M^0^) derivatives of YJM1133, YJM1326 and YJM1399 there were slight differences in K1 sensitivity for the three strong K1 killer strains.

Table S6:

*S. cerevisiae* M^+^ vs. M^0^ killing of *C. glabrata*

column A (Strain): Isogenic L-A^+^ M^+^ and L-A^0^ M^0^ strain pairs

column B (Background): genetic backgrounds of each of the strain pairs

column C (L-A): 1 = L-A^+^; 0 = L-A^0^

column D (M): 1 = M1; 2 = M2; lus = Mlus; novel; 0 = M^0^

column E (pH 4.7): killing of *C. glabrata* at pH 4.7

column F (pH 6.0): killing of *C. glabrata* at pH 6.0

scoring: killing of *C. glabrata* LAWNS by strains; kill zone **SIZE**: 0, 0.5 (dk blue ring), 1, 2

M1: While the three K1^+^ strains (YJM1077, 1290, 1307) kill *C. glabrata*, the M^0^ derivatives show greatly reduced or no killing. Both the K1^±^ (YJM1387) strain and its M^0^ derivative show weak killing of *C. glabrata*. Neither the K1^-^ strain (YJM1419) nor its M^0^ derivative kill *C. glabrata*.

M2: While the three K2^+^ strains kill *C. glabrata*, their M^0^ derivatives show weaker but still substantial killing of *C. glabrata*.

Mlus: For six of the seven backgrounds, the Mlus^+^ parents and their M^0^ derivatives show equivalent killing of *C. glabrata* at pH 4.7. Klus^+^ YJM320 shows Mlus-dependent killing of *C. glabrata* at pH 4.7; its M^0^ derivative does not kill *C. glabrata*. Klus^+^ YJM1133 shows Mlus-dependent killing of *C. glabrata* at pH 6 but Mlus^+^ and M^0^ derivatives show equivalent killing of *C. glabrata* at pH 4.7.

novel M: in both the YJM195 and YJM681 backgrounds, M^+^ and M^0^ derivatives show no differential killing of *C. glabrata*.

*KHR1*

Column A: Strain (Background).

Column B: *KHR1* ORF: + = *KHR1* ORF present; 50 ochre = *khr1* ORF present but with premature stop polymorphism at codon 50; 0 = *khr1* ORF absent.

Column C: There was no killing of *C. glabrata* ATCC 2001 pH 6 (20º, 2 days) on both SD and YPD, both containing 0.1 M citrate (pH adjusted with K_3_PO_4_).

Rows 2 – 29: L-A^0^ M^0^ derivatives of L-A^+^ M^+^ 100-genomes strains (parental background).

Rows 31 and 32: YJM1463 and YJM1529, two L-A^+^ M^0^ strains where L-A could not be cured

Rows 30 and 33 - 101: Naturally L-A^0^ M^0^ 100-genomes strains.

*KHS1*

Column A: Strain (Background).

Column B: *KHS1* ORF species origin: *S. cerevisiae* or *S. paradoxus*; YJM326 has no *KHS1* sequences (*khs1*∆).

Column C: *KHS1* ORF premature stop codon polymorphism; codon # and type of stop.

Column D: killing of *C. glabrata* (pH 4.7), killing zone bins (0, 0.5, 1, 2).

Column E: killing by YJM189 *KHS1* (pH 4.7), killing zone bins (0, 0.5, 1).

Colum F: killing by YJM1896 *khs1*∆ (pH 4.7): all killing abolished.

Rows 2 - 29 = L-A^0^ (M^0^) derivatives of L-A^+^ (M^+^) 100-genomes strains (parental background).

Rows 31 and 32: YJM1463 and YJM1529, two L-A^+^ M^0^ strains where L-A could not be cured.

Rows 30 and 33 - 101: Naturally L-A^0^ M^0^ 100-genomes strains.

Table S7: Fast GWAS of *C. glabrata* killing by M^0^ 100-genomes strains phenotypes.

Table S8: Fast GWAS of YJM189 *KHS1* killing of M^0^ 100-genomes strains phenotypes.
