# Supplementary figures and images for: RNA viruses, M satellites, chromosomal killer genes, and killer/nonkiller phenotypes in the 100-genomes S. cerevisiae strains
Source: G3 (Bethesda). 2023 Jul 27;13(10):jkad167. doi: 10.1093/g3journal/jkad167 (PMC10542562; doi:10.1093/g3journal/jkad167)

A.

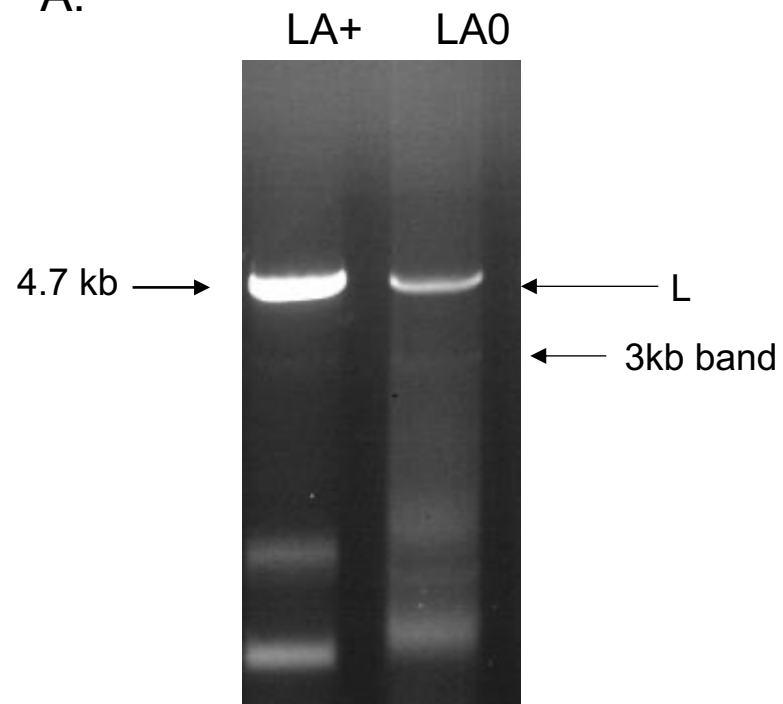

B.

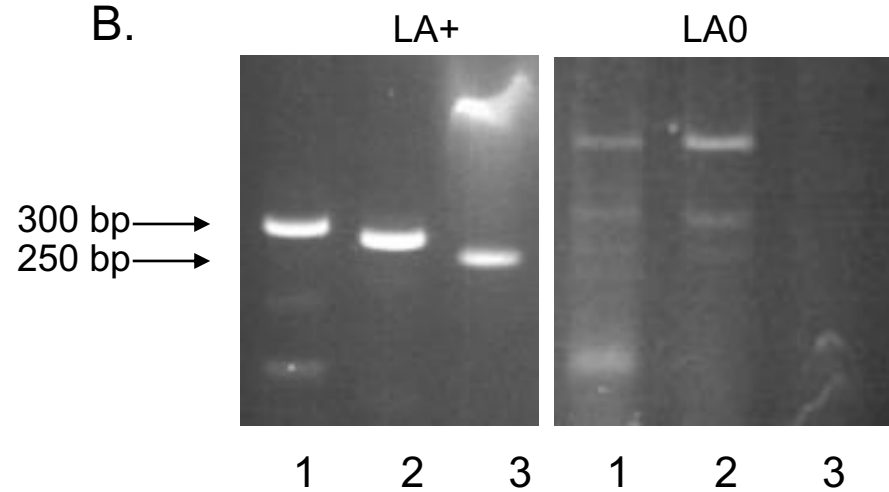

Fig S2

C.

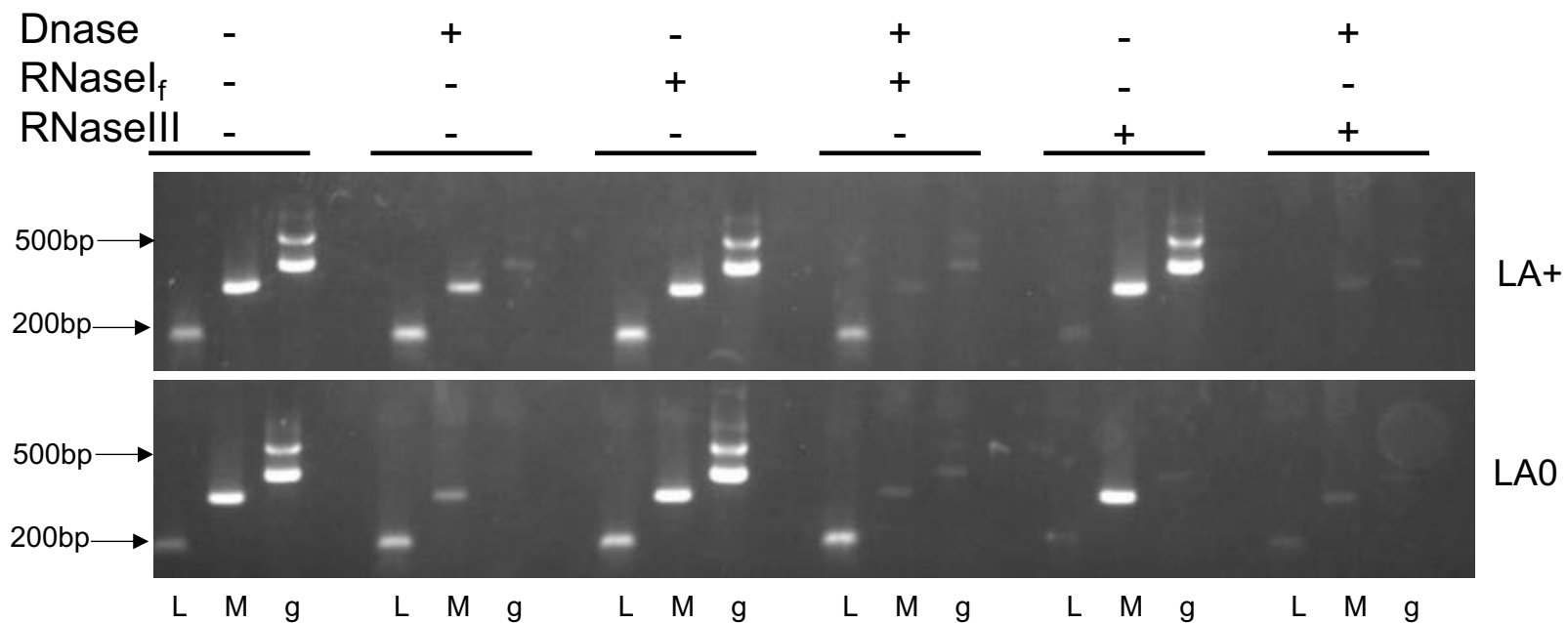

Supplement: jkad167_Supplementary_Data [file jkad167_supplementary_data.zip › Figure_S2_G3-2023-404116.pdf]

**Fig S3**

**A.**

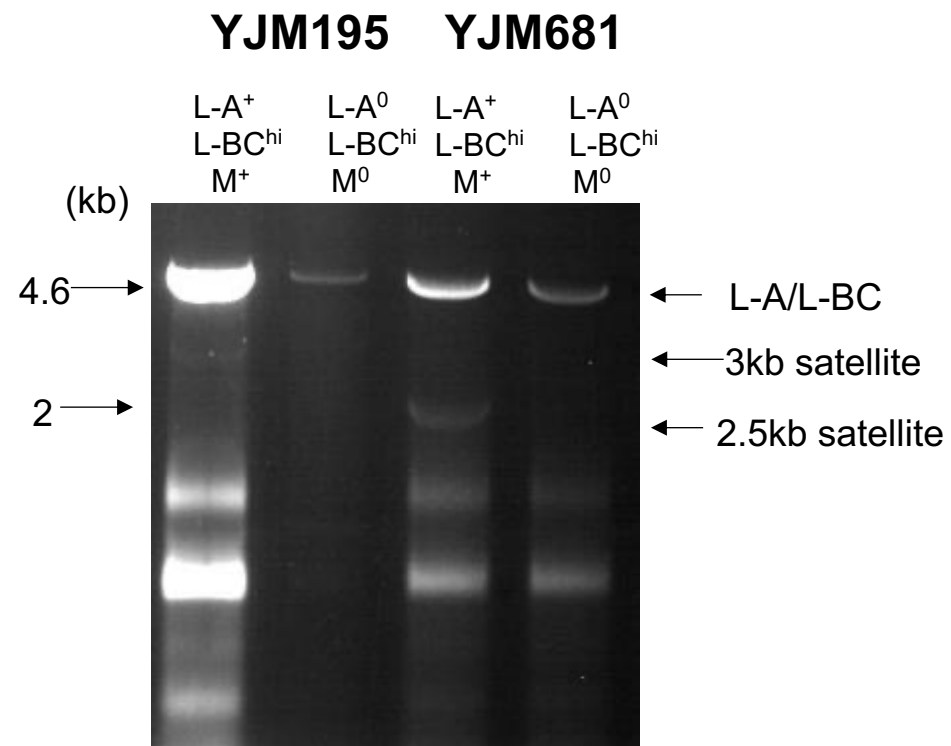

**B.**

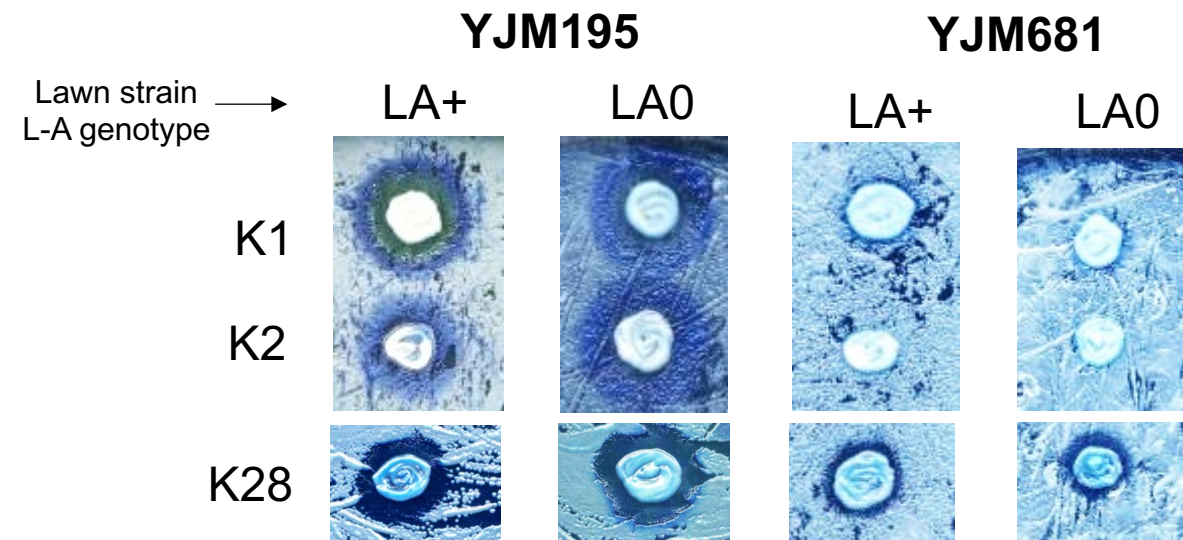

Supplement: jkad167_Supplementary_Data [file jkad167_supplementary_data.zip › Figure_S3_G3-2023-404116.pdf]

Fig S4

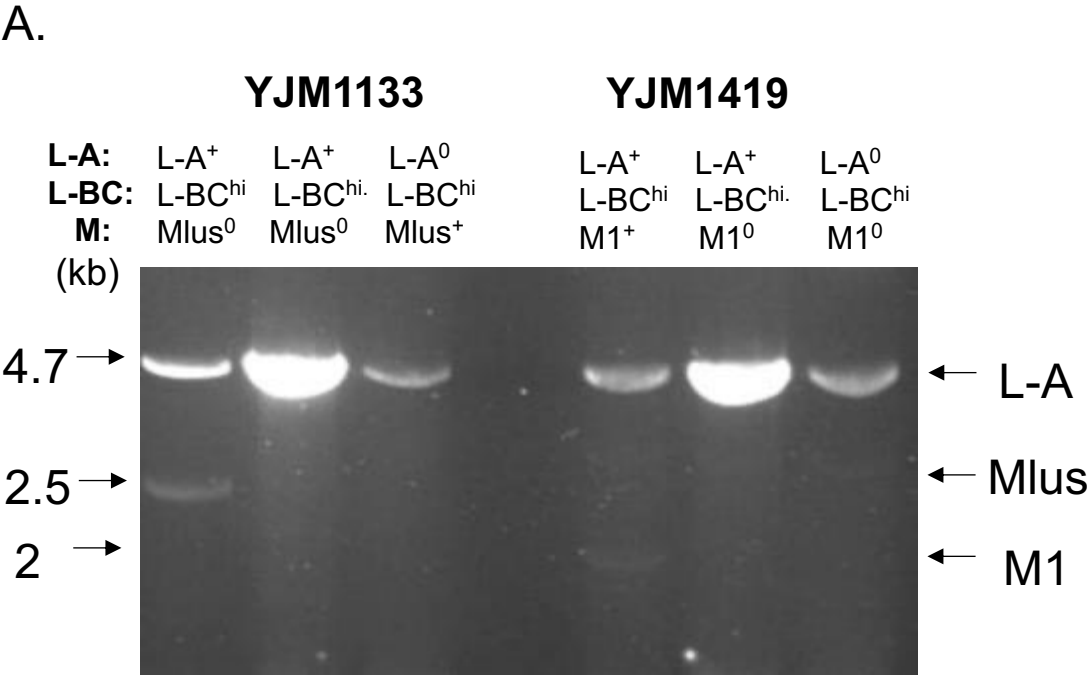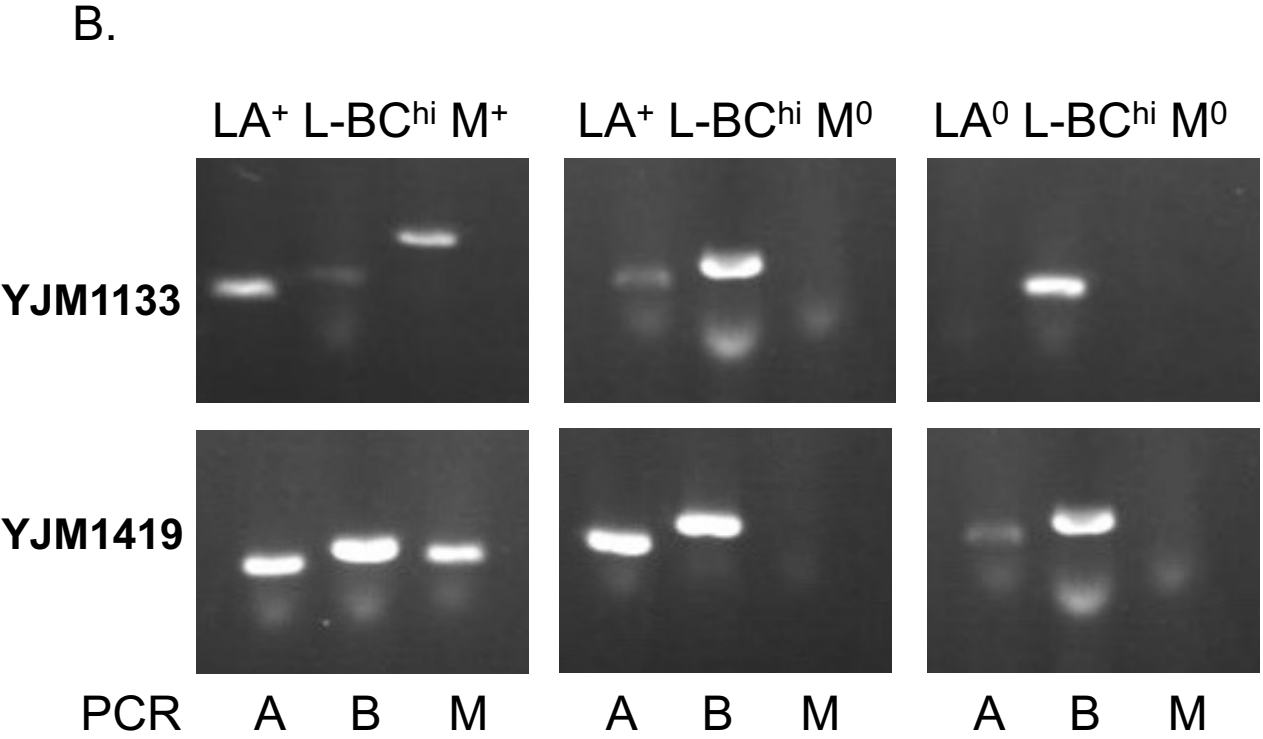

Supplement: jkad167_Supplementary_Data [file jkad167_supplementary_data.zip › Figure_S4_G3-2023-404116.pdf]

**Fig. S6**

**A**

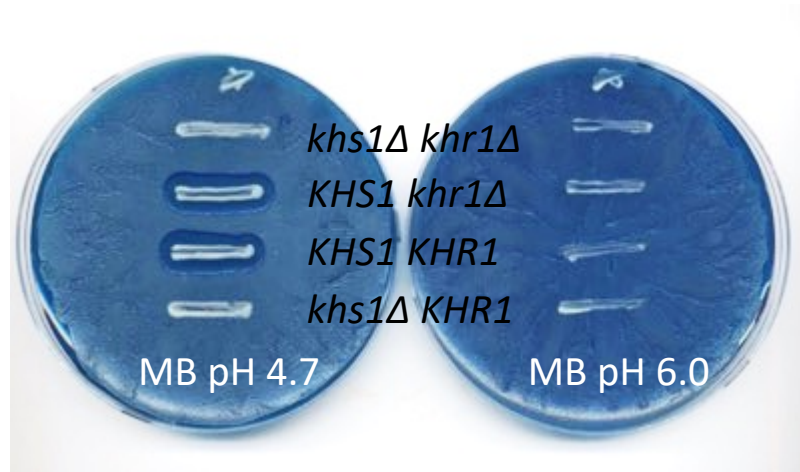

**B**

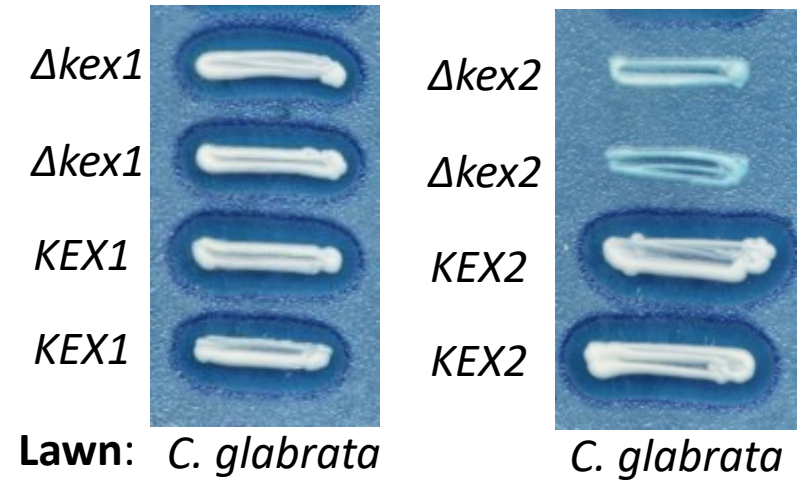

**C**

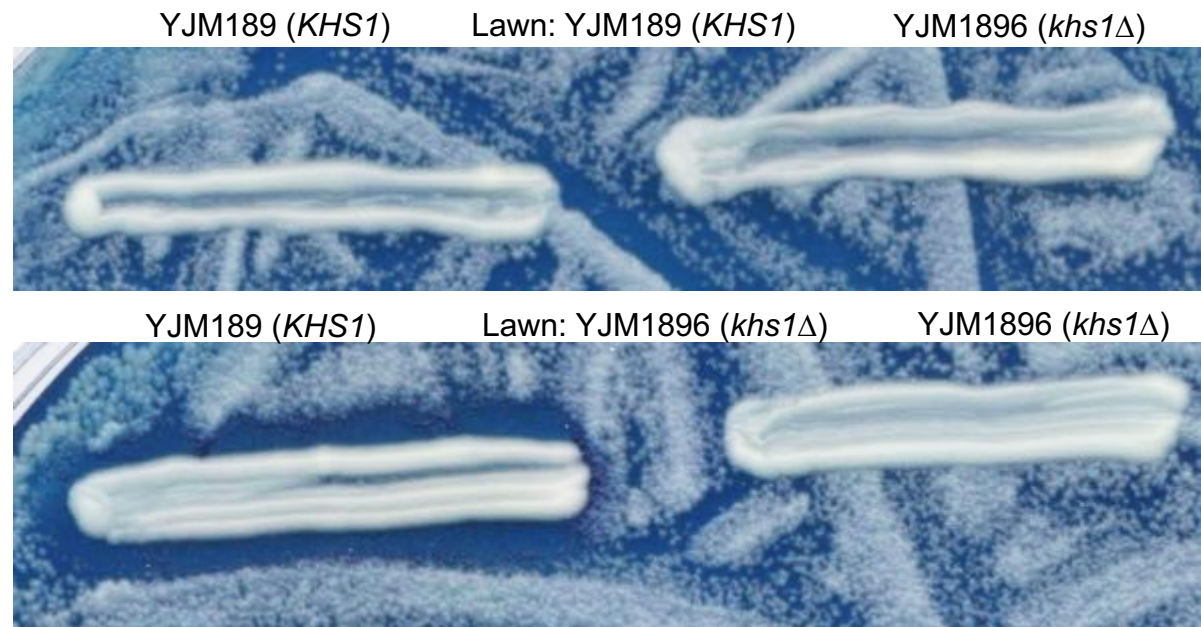

Supplement: jkad167_Supplementary_Data [file jkad167_supplementary_data.zip › Figure_S6_G3-2023-404116.pdf]

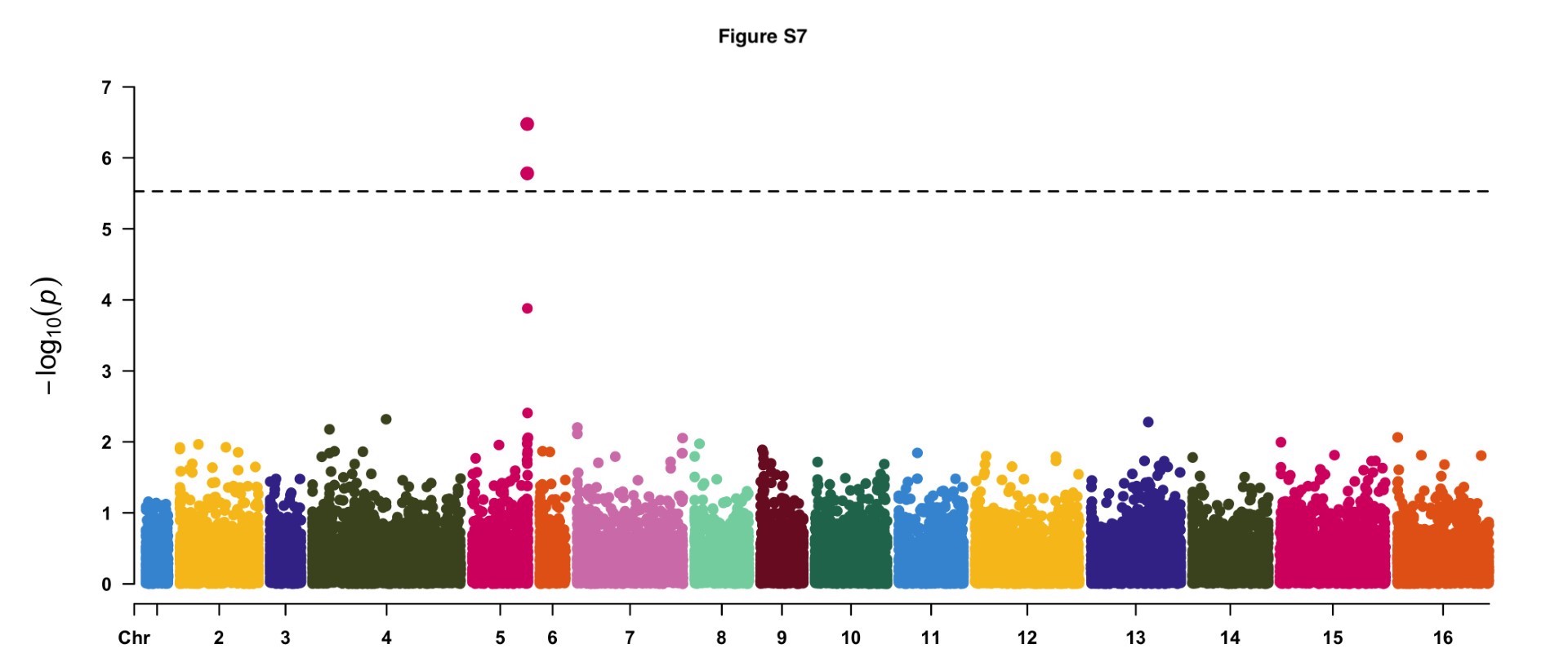

Supplement: jkad167_Supplementary_Data [file jkad167_supplementary_data.zip › Figure_S7_G3-2023-404116.jpg]

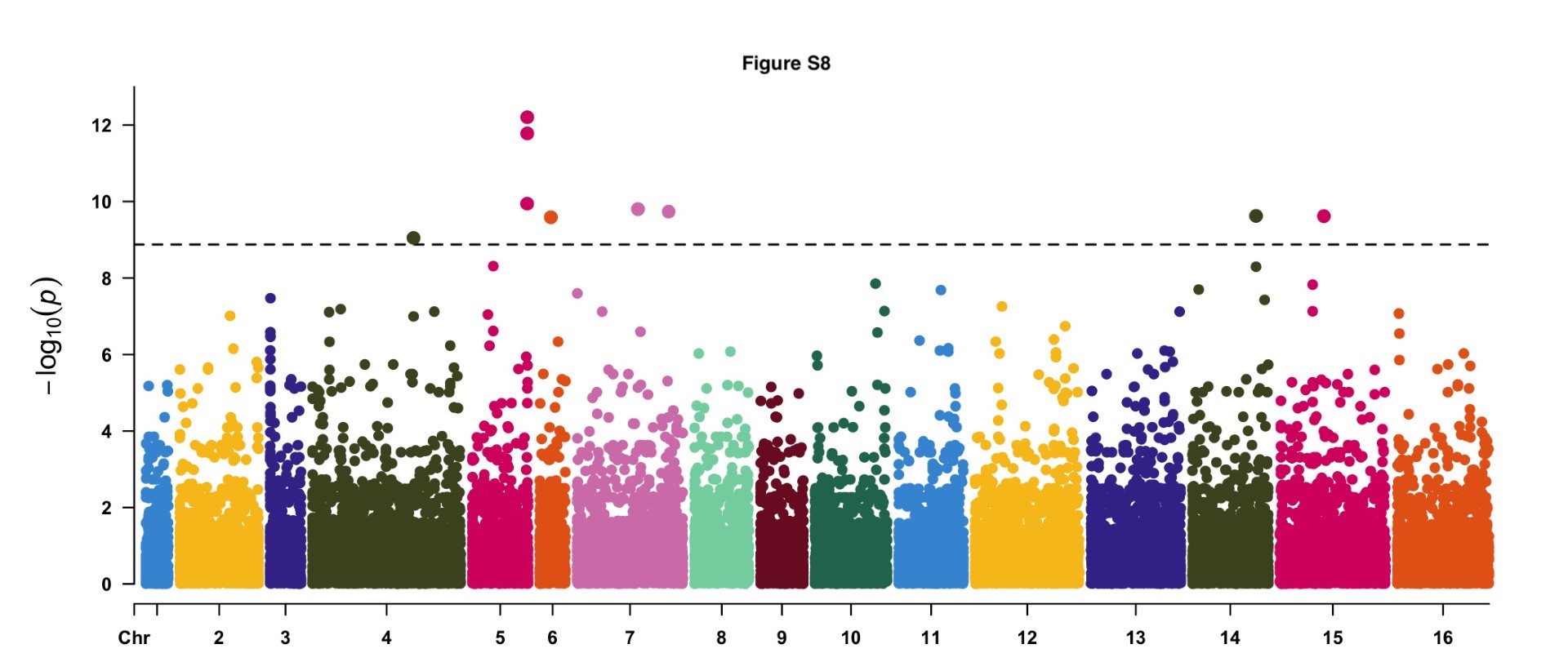

Supplement: jkad167_Supplementary_Data [file jkad167_supplementary_data.zip › Figure_S8_G3-2023-404116.jpg]
